# Developing prediction models for electrolyte abnormalities in patients indicated for antihypertensive therapy: evidence-based treatment and monitoring recommendations

**DOI:** 10.1097/HJH.0000000000004032

**Published:** 2025-05-07

**Authors:** Ariel Wang, Constantinos Koshiaris, Lucinda Archer, Richard D. Riley, Kym I.E. Snell, Richard Stevens, Amitava Banerjee, Juliet A. Usher-Smith, Subhashisa Swain, Andrew Clegg, Christopher E. Clark, Rupert A. Payne, F.D. Richard Hobbs, Richard J. McManus, James P. Sheppard

**Affiliations:** a Nuffield Department of Primary Care Health Sciences, University of Oxford, Oxford, UK; b Department of Primary Care and Population Health, University of Nicosia Medical School, Nicosia, Cyprus; c National Institute for Health and Care Research (NIHR), Birmingham Biomedical Research Centre; d Department of Applied Health Sciences, School of Health Sciences, College of Medicine and Health, University of Birmingham, Birmingham; e Institute of Health Informatics, University College London, London; f Primary Care Unit, Department of Public Health and Primary Care, University of Cambridge, Cambridge; g Academic Unit for Ageing and Stroke Research, Bradford Institute for Health Research, University of Leeds, Leeds; h Exeter Collaboration for Academic Primary Care, University of Exeter Medical School, St Luke's Campus, Exeter, UK

**Keywords:** antihypertensive therapy, clinical decision-making, drug-related adverse effects, serum electrolytes monitoring

## Abstract

**Objectives:**

Evidence from clinical trials suggests that antihypertensive treatment is associated with an increased risk of common electrolyte abnormalities. We aimed to develop and validate two clinical prediction models to estimate the risk of hyperkalaemia and hyponatraemia, respectively, to facilitate targeted treatment and monitoring strategies for individuals indicated for antihypertensive therapy.

**Design and methods:**

Participants aged at least 40 years, registered to an English primary care practice within the Clinical Practice Research Datalink (CPRD), with a systolic blood pressure reading between 130 and 179 mmHg were included the study. The primary outcomes were first hyperkalaemia or hyponatraemia event recorded in primary or secondary care. Model development used a Fine-Gray approach with death from other causes as competing event. Model performance was assessed using C-statistic, D-statistic, and Observed/Expected (O/E) ratio upon external validation.

**Results:**

The development cohort included 1 773 224 patients (mean age 59 years, median follow-up 6 years). The hyperkalaemia model contained 23 predictors and the hyponatraemia model contained 29 predictors, with all antihypertensive medications associated with the outcomes. Upon external validation in a cohort of 3 805 366 patients, both models calibrated well (O/E ratio: hyperkalaemia 1.16, 95% CI 1.13–1.19; hyponatraemia 1.00, 95% CI 0.98–1.02) and showed good discrimination at 10 years (C-statistic: 0.69, 95% CI 0.69–0.69; 0.80, 95% CI 0.80–0.80, respectively).

**Conclusion:**

Current clinical guidelines recommend monitoring serum electrolytes after initiating antihypertensive treatment. These clinical prediction models predicted individuals’ risk of electrolyte abnormalities associated with antihypertensive treatment and could be used to target closer monitoring for individuals at a higher risk, where resources are limited.

## INTRODUCTION

Hypertension is the most prevalent chronic health condition and the leading risk factor for cardiovascular disease [1]. Although antihypertensive treatment has been shown to reduce the risk of cardiovascular events and death in all ages up to 85 years [2], it is also associated with potential harms, such as hypotension, syncope, falls, acute kidney injury, and electrolyte abnormalities [3,4]. Previous studies have been shown that antihypertensive medication prescription is associated with common electrolyte abnormalities, including hyponatraemia, hypokalaemia, hyperkalaemia, and hypercalcaemia [5–7]. In particular, thiazide-type diuretics, beta-blockers, and blockers of the renin-angiotensin-aldosterone system (RAAS) are thought to be more likely to cause these complications [5–7].

Although these electrolyte abnormalities are often asymptomatic, they are relatively common, particularly in older people and patients with comorbidities such as renal disease and diabetes [6,8–10]. If left undetected, they can lead to serious complications. For example, hyperkalaemia, defined as a serum potassium concentration exceeding 5.5 mmol/l [8], is associated with prescription of RAAS medications [3] and has been shown to increase the risk of life-threatening cardiac arrhythmias or cardiac arrest [8,9]. Hyponatraemia, defined as a serum sodium concentration below 135 mmol/l [11], is the most frequently observed electrolyte abnormality in clinical practice, affecting 5–35% of the adult population [11–14]. Mild chronic hyponatraemia is associated with an increased rate of syncope, falls, and fractures, while severe acute hyponatraemia is associated with increased mortality, morbidity, risk of hospitalization, and length of hospital stay [7,12,14]. Certain medications, especially thiazide and thiazide-like diuretics, are more likely to cause hyponatraemia [7,15,16].

Strategies to prevent these drug-induced electrolyte abnormalities involve careful consideration of risk factors along with clinical and laboratory evaluation before initiation and during treatment. For individuals at a high risk of hyperkalaemia or hyponatraemia, it may not be appropriate to prescribe specific antihypertensive medications. For those already undergoing treatment, transitioning to an alternative class may be considered. To enable optimal treatment choice, clinicians must understand an individual's underlying risk of these electrolyte abnormalities.

Therefore, the present study aimed to develop and externally validate two new prediction models for the risk of hyperkalaemia and hyponatraemia, taking into account the competing risk of death from other causes.

## MATERIALS AND METHODS

Extended methods for this study are described in detail in Supplementary appendix 1.

### Design

This study used a retrospective observational cohort design using routine primary care electronic health records (EHRs) data from the Clinical Practice Research Datalink (CPRD) in the UK. Patient-level data were linked to Office for National Statistics (ONS) Death Registration Data, Hospital Episode Statistics (HES), and Index of Multiple Deprivation (IMD) data. The two prediction models were developed using the CPRD GOLD data (patient EHR from general practice surgeries using the Vision software system), with external validation conducted using the CPRD Aurum data (patient EHR from GP surgeries using EMIS software system) [17,18]. These two distinctive primary care databases are representative of the UK population in terms of age, sex, and ethnicity [17,18]. CPRD GOLD comprises 11.3 million patients (4.4 million currently alive) from 674 practices, while CPRD Aurum comprises 19 million patients (7 million currently alive) from 738 practices [17,18]. The protocol for this study was approved by the CPRD Independent Scientific Advisory Committee (ISAC) (protocol number 19_042, see appendix 5).

### Population

Participants aged at least 40 years registered to a CPRD practice between 1 January 1998 and 31 December 2018 with at least one systolic blood pressure (SBP) measurement between 130 and 179 mmHg were eligible for inclusion. The index date was defined at 12 months after cohort entry (first high blood pressure measure) and the study follow-up period was up to 10 years. All patient characteristics and predictors for the models were determined at the index date. Patients exited the cohort on the study end date (31 December 2018), or upon transferring out of a registered CPRD practice, death, or after experiencing the specific outcome of interest. The same eligibility criteria and methods were applied to both the development and validation cohorts.

### Outcomes

The primary outcomes were first hyperkalaemia or hyponatraemia event within 10 years of index date. Hyperkalaemia was defined using a combination of test result (serum potassium >5.5 mmol/l) or diagnosis codes of hyperkalaemia in CPRD, HES and ONS within 10 years of the index date. Similarly, hyponatraemia was also defined using a combination of test result (serum sodium <135 mEq/l) or diagnosis codes of hyponatraemia in CPRD, HES and ONS within the same time frame (Clinical codes for outcomes, see Supplementary appendix three Table S1). Prespecified secondary outcomes were hyperkalaemia or hyponatraemia (defined in the same way) within 1 and 5 years of the index date. The definition of outcomes was consistent with consensus clinical practice and guidelines [8,11].

### Model covariates

Predictors of hyperkalaemia and hyponatraemia were prespecified and defined according to previous literature and expert clinical opinion (List of predictors, see Supplementary appendix 3 Table S2). A total of 25 predictors were considered for the hyperkalaemia model and 29 for the hyponatraemia model. These included patient demographics, clinical characteristics, comorbidities and prescribed medications including antihypertensives. Covariates were defined as the most recent relevant clinical code before the index date, with the exception of blood test results (serum creatinine, potassium and sodium) and a previous history of hyperkalaemia and hyponatraemia, which were captured in the 2 years prior to the index date. Medication prescriptions, including antihypertensives, were defined as any prescription within 1 year prior to the index date.

### Sample size

A sample size of approximately 16 778 patients was estimated to be required for the development of the risk equations. The sample size calculation was based on an event rate of between 14.6 and 36 per 1000 patient years of follow-up [6,9], an expected median follow-up of 7 years [19], an estimate of Nagelkerke's *R*^2^ statistic of 0.15, a global shrinkage factor of 0.9 and a maximum number of 40 parameters in the model [20]. For external validation, a prognostic model requires ideally 200 or more events [21]. The actual sample sizes in both development and validation cohorts far exceeded these estimates.

### Statistical analysis

The study was conducted in accordance with the TRIPOD (Transparent Reporting of a multivariable prediction model for Individual Prognosis or Diagnosis) guidelines [22] for prediction model studies (See Supplementary appendix 2. TRIPOD Checklist). Baseline characteristics were summarised using descriptive statistics in the development and validation cohorts separately.

### Model development

Each model was developed and internally validated by researchers at the University of Oxford (A.W., C.K., J.P.S.). Multivariable prediction models were fitted in each imputed dataset using a Fine-Gray sub-distribution hazard model, which accounted for the competing risk of death by other causes [23]. Sub-distribution hazard ratios (SHRs) with 95% confidence intervals were reported, and the postestimation baseline cumulative incidence for each event was estimated using a Breslow-type estimator as defined in the Fine-Gray paper [23]. Analyses were conducted using the *fastcmprsk* package in RStudio [24]. Fractional polynomials were used to examine the linearity assumption of all continuous variables (age, SBP and electronic frailty index [eFI]) and identify the best fitting transformation [25]. Automated variable selection methods were not used since all predictors were prespecified; instead, a post hoc decision was used.

### Apparent validation using development data

Apparent validation was assessed using calibration plots comparing the observed to predicted probabilities at 1, 5 and 10 years. Observed outcome probabilities were estimated using pseudo-values: jack-knife estimators representing an individual's contribution to the cumulative incidence function for each event accounting for the competing risk of death and calculated by the Aalen–Johansen method [26]. Calibration plots were produced using the pseudo-values and generated using a loess smoother calibration curve with 95% confidence intervals. Where miscalibration was present upon assessment of apparent performance, recalibration in the development dataset was considered.

### External validation

The external validation of each prediction model was conducted by researchers at The University of Birmingham (LA, KIES, RDR), independent of the model development team. The prediction model algorithms (Supplementary appendix 4 equations) were applied to each individual in the external validation cohort to give the predicted probabilities of experiencing a hyperkalaemia or hyponatraemia event within 1, 5 and 10 years, taking account of the competing risk of death by other causes. Model calibration was assessed using the same method as used in the apparent validation. Model performance was assessed using the Observed to Expected ratio (O/E), Harrell's C-statistic and Royston's D-statistic with its associated *R*^2^ statistic [27], applied to the same pseudo-values as above, along with calibration plots. Heterogeneity in model performance across different GP practices was assessed.

### Clinical utility analysis

Further analyses compared the 10-year risk of hyperkalaemia or hyponatraemia event against the risk of cardiovascular disease, calculated using the QRisk2 algorithm using a 10% threshold [28]. Clinical utility was assessed using net benefit analysis to examine the benefits of using the STRATIFY prediction models for clinical decision making on regular serum electrolytes monitoring [29]. The STRATIFY prediction models were compared with model blind methods of no regular monitoring (which may involve remove current guidelines on regular serum electrolytes checking) for all patients, or regular monitoring (starting or continuing) for all patients, regardless of risk. Venn diagrams were used to visualised the overlap of patients at high-risk (≥10%) hyperkalaemia, hyponatraemia and cardiovascular disease in the CPRD Gold cohort.

### Missing data

Multiple imputation with chained equations was used to impute missing data in both the derivation and validation dataset. Ten imputations were generated for each cohort and imputation models included all covariates within each dataset, along with the Nelson-Aalen estimator, and outcomes of interest (hyperkalaemia or hyponatraemia, and the competing event of death in each model) [30,31]. Predictor variables requiring imputation were ethnicity, BMI groups, deprivation score (validation cohort only), smoking status, alcohol consumption and estimate glomerular filtration rate (eGFR) categories (calculated using The 2021 CKD-EPI creatinine equation) [32].

## RESULTS

### Population characteristics

A total of 1 773 224 patients were included in the model development cohort (CPRD GOLD) with a mean age of 59 years (SD 13.2), and median follow up of 6 years (IQR 2.6–10) (Table 1). The mean blood pressure at study entry was 144/84 mmHg (SD 12/10 mmHg). The 10-year prevalence of hyperkalaemia was 6.9% (*n* = 122 775), with 14.7% (*n* = 261 264) of patients experiencing the competing event of death from other causes. The 10-year prevalence of hyponatraemia was 10.7% (*n* = 190 116), with 12.6% (*n* = 223 575) of patients experiencing the competing event of death from other causes.

**TABLE 1:** Baseline characteristics of patients in the development dataset (CPRD Gold)

Variable	Total (*N* = 1 773 224)	Hyperkalaemia (*n* = 122 775)	Competing risk - hyperkalaemia (*n* = 261 264)	Hyponatraemia (*n* = 190 116)	Competing risk - hyponatraemia (*n* = 223 575)
Age, years – mean (SD)	59.4 (13.2)	65 (12 5)	75 (12.2)	67.9 (12.8)	74.9 (12.3)
SBP, mmHg – mean (SD)	143.5 (12.0)	145.9 (12 6)	146.7 (12.7)	147.1 (12.9)	146.5 (12.7)
DBP, mmHg – mean (SD)	83.8 (9.6)	83 (10)	81.6 (10.1)	83 (10)	81.5 (10.1)
Follow-up, years – median (IQR)	6.2 (2.6–10)	4.0 (1.8–6.5)	4.3 (2–7)	3.5 (1.4–6.2)	4.1 (1.9–6.8)
Electronic frailty index^a^ – mean (SD)	0.06 (0.08)	0.08 (0.08)	0.1 (0.08)	0.09 (0.11)	0.1 (0.08)
Sex					
Male	851 058 (48%)	63 069 (51.4%)	122 322 (46.8%)	83 630 (44%)	107 409 (48%)
Female	922 166 (52%)	59 706 (48.6%)	138 942 (53.2%)	106 486 (56%)	116 166 (52%)
Ethnicity					
White	734 401 (41.4%)	79 770 (65%)	167 832 (64.2%)	138 154 (72.7%)	140 483 (62.8%)
Black	10 802 (0.6%)	1073 (0.9%)	1242 (0.5%)	1101 (0.6%)	1,173 (0.5%)
South Asian	14 805 (0.8%)	2555 (2.1%)	1359 (0.5%)	2913 (1.5%)	1,184 (0.5%)
Other	15 737 (0.9%)	1761 (1.4%)	1961 (0.8%)	2110 (1.1%)	1,760 (0.8%)
Missing	997 479 (56.3%)	37 616 (30.6%)	88 870 (34%)	45 838 (24.1%)	78,975 (35.3%)
Deprivation Score					
IMD 1	419 468 (23.7%)	27 019 (22%)	51 353 (19.7%)	40 455 (21.3%)	43,374 (19.4%)
IMD 2	406 916 (22.9%)	27 973 (22.8%)	57 398 (22%)	42 346 (22.3%)	48,917 (21.9%)
IMD 3	376 903 (21.3%)	25 703 (20.9%)	56 578 (21.7%)	40 497 (21.3%)	48,571 (21.7%)
IMD 4	313 707 (17.7%)	22 874 (18.6%)	50 150 (19.2%)	35 662 (18.8%)	43,238 (19.3%)
IMD 5	254 800 (14.4%)	19 128 (15.6%)	45 494 (17.4%)	30 986 (16.3%)	39,227 (17.5%)
Missing	1430 (0.1%)	78 (0.06%)	291 (0.1%)	170 (0.09%)	248 (0.1%)
BMI					
Underweight	20 635 (1.2%)	1479 (1.2%)	7924 (3%)	3798 (2%)	6,471 (2.9%)
Normal	519 524 (29.3%)	32 952 (26.8%)	80 161 (30.7%)	60 535 (31.8%)	66,072 (29.6%)
Overweight	586 531 (33.1%)	40 996 (33.4%)	69 964 (26.8%)	58 256 (30.6%)	60,169 (26.9%)
Obese	340 357 (19.2%)	25 972 (21.2%)	33 883 (13%)	32 442 (17.1%)	30,334 (13.6%)
Morbidly obese	39 853 (2.2%)	3553 (2.9%)	3599 (1.4%)	4012 (2.1%)	3,400 (1.5%)
Missing	266 324 (15%)	17 823 (14.5%)	65 733 (25.2%)	31 073 (16.3%)	57,129 (25.6%)
Smoking status					
Non smoker	847 473 (47.8%)	12 107 (42.6%)	79 576 (41.5%)	18 843 (47.2%)	78,886 (41.2%)
Ex-smoker	471 193 (26.6%)	8907 (31.3%)	53 345 (27.8%)	10 817 (27.1%)	53,716 (28.1%)
Smoker	363 579 (20.5%)	5537 (19.5%)	39 653 (20.7%)	7236 (18.1%)	39,852 (20.8%)
Missing	90,979 (5.1%)	1,899 (6.7%)	19,191 (10.0%)	3,002 (7.5%)	18,980 (9.9%)
Alcohol					
Non drinker	289,581 (16.3%)	24,706 (20.1%)	56,299 (21.5%)	37,729 (19.8%)	48,713 (21.8%)
Trivial drinker	488,448 (27.5%)	32,918 (26.8%)	59,723 (22.9%)	49,249 (25.9%)	50,909 (22.8%)
Light drinker	239,799 (13.5%)	14,709 (12%)	26,483 (10.1%)	22,618 (11.9%)	22,400 (10%)
Moderate drinker	179,162 (10.1%)	10,781 (8.8%)	17,657 (6.8%)	16,598 (8.7%)	14,544 (6.5%)
Heavy drinker	22,772 (1.3%)	1,536 (1.3%)	3,489 (1.3%)	3,348 (1.8%)	2,613 (1.2%)
Unknown amount	291,767 (16.5%)	19,418 (15.8%)	38,923 (14.9%)	29,819 (15.7%)	33,275 (14.9%)
Missing	261,695 (14.8%)	18,707 (15.2%)	58,690 (22.5%)	30,755 (16.2%)	51,121 (22.9%)
Frailty index groups^a^					
Fit	1,551,140 (87.5%)	93,603 (76.2%)	171,732 (65.7%)	140,219 (73.8%)	146,834 (65.7%)
Mildly frail	192,855 (10.9%)	24,986 (20.4%)	72,154 (27.6%)	42,619 (22.4%)	61,697 (27.6%)
Moderately frail	26,437 (1.5%)	3,766 (3.1%)	15,459 (5.9%)	6,518 (3.4%)	13,414 (6%)
Severely frail	2,792 (0.2%)	420 (0.3%)	1,919 (0.7%)	760 (0.4%)	1,630 (0.7%)
eGFR (CKD stages)					
Stage 1 (G1) – normal	283,294 (16%)	14,723 (12%)	18,634 (7.1%)	24,912 (13.1%)	14,696 (6.6%)
Stage 2 (G2) – mild reduction, normal if young	433,111 (24.4%)	35,084 (28.6%)	65,239 (25%)	55,416 (29.1%)	53,589 (24%)
Stage 3a (G3a) – mild-moderate reduction	87,522 (4.9%)	13,271 (10.8%)	32,966 (12.6%)	19,054 (10%)	28,614 (12.8%)
Stage 3b (G3b) – moderate-severe reduction	28,303 (1.6%)	6,357 (5.2%)	15,437 (5.9%)	7,068 (3.7%)	14,376 (6.4%)
Stage 4 (G4) – severe reduction	6,620 (0.4%)	2,086 (1.7%)	3,660 (1.4%)	1,515 (0.8%)	3,929 (1.8%)
Stage 5 (G5) – kidney failure	1,229 (0.1%)	395 (0.3%)	610 (0.2%)	296 (0.2%)	656 (0.3%)
No test	933,145 (52.6%)	50,859 (41.4%)	124,718 (47.7%)	81,855 (43.1%)	107,715 (48.2%)
Previous Hyperkalaemia within 2 years	13,918 (0.8%)	4,502 (3.7%)	2,842 (1.1%)	··	··
Previous Hyponatraemia within 2 years	30,301 (1.7%)	··	··	17,268 (9.1%)	6,083 (2.7%)
Chronic diseases					
Heart Failure	31,338 (1.8%)	5,359 (4.4%)	18,402 (7%)	7,611 (4%)	16,387 (7.3%)
Diabetes	137,781 (7.8%)	26,475 (21.6%)	31,480 (12%)	35,040 (18.4%)	27,705 (12.4%)
Cerebrovascular disease	64,469 (3.6%)	7,515 (6.1%)	31,203 (11.9%)	13,250 (7%)	27,282 (12.2%)
Coronary artery disease	143,286 (8.1%)	21,053 (17.1%)	50,789 (19.4%)	··	··
Peripheral vascular disease	30,120 (1.7%)	4,863 (4%)	13,795 (5.3%)	··	··
Chronic liver disease	6,546 (0.4%)	··	··	1,482 (0.8%)	1,388 (0.6%)
Antihypertensive drugs					
ACE inhibitors	219,588 (12.4%)	30,754 (25%)	50,604 (19.4%)	43,687 (23%)	43,592 (19.5%)
Angiotensin II receptor antagonists	59,103 (3.3%)	7,435 (6.1%)	11,256 (4.3%)	11,476 (6%)	9,384 (4.2%)
Alpha blockers	34,349 (1.9%)	4,652 (3.8%)	9,095 (3.5%)	7,329 (3.9%)	7,783 (3.5%)
Beta blockers	216,202 (12.2%)	24,190 (19.7%)	43,685 (16.7%)	37,681 (19.8%)	36,873 (16.5%)
Calcium channel blockers	193,221 (10.9%)	21,740 (17.7%)	50,191 (19.2%)	35,614 (18.7%)	42,681 (19.1%)
Loop diuretics	107,018 (6%)	15,688 (12.8%)	54,589 (20.9%)	23,721 (12.5%)	48,249 (21.6%)
Potassium sparing diuretics	41,993 (2.4%)	5,733 (4.7%)	19,711 (7.5%)	10,934 (5.8%)	16,315 (7.3%)
Thiazides and thiazide-like diuretics	180,115 (10.2%)	15,433 (12.6%)	43,526 (16.7%)	40,269 (21.2%)	33,651 (15.1%)
Other antihypertensives	10,884 (0.6%)	2,794 (2.3%)	5,775 (2.2%)	4,211 (2.2%)	4,797 (2.1%)
Antidepressant	189,758 (10.7%)	··	··	25,485 (13.4%)	32,205 (14.4%)
Antipsychotic	36,333 (2%)	··	··	5,671 (3%)	12,707 (5.7%)
Anticonvulsants	34,367 (1.9%)	··	··	7,690 (4%)	7,562 (3.4%)
Proton pump inhibitors	259,410 (14.6%)	··	··	36,969 (19.4%)	43,034 (19.2%)
NSAIDs	343,941 (19.4%)	··	··	41,387 (21.8%)	41,116 (18.4%)

ACE, Angiotensin converting enzyme; BMI, Body mass index; CKD, Chronic kidney disease; eGFR, estimated glomerular filtration rate; IMD, Indicies of multiple deprivation; NSAIDS, Nonsteroidal anti-inflammatory drugs.

a The electronic frailty index (eFI) includes 36 items and is estimated from electronic health records. The index ranges from 0 to 1 (“fit” 0 ≤ eFI ≤ 0.12; “mild” 0.12<eFI ≤ 0.24; “moderate” 0.24 < eFI ≤ 0.36; “severe” 0.36 < eFI ≤ 1.0).

A total of 3 805 366 patients were included in the validation cohort (CPRD Aurum), with a mean age of 59 years (SD 13.3), and median follow up of 7 years (IQR 2.9–10) (Supplementary appendix 3 Table S3). The 10-year prevalence of hyperkalaemia was 7.3% (*n* = 277 982), with 9.4% (*n* = 356 193) of patients experiencing the competing event of death from other causes. The 10-year prevalence of hyponatraemia was 11.1% (*n* = 424 126), with 7.9% (*n* = 298 889) of patients experiencing the competing event of death from other causes. Ethnicity data were more complete in the validation cohort compared to the development cohort (81 vs. 44% complete data).

### Model development

#### STRATIFY-Hyperkalaemia model

A total of 23 predictors were included in the final STRATIFY-Hyperkalaemia model, after the exclusion of covariates with no association with hyperkalaemia (alcohol consumption and IMD). Previous hyperkalaemia (sub-hazard ratio [SHR] 3.64, 95% confidence interval [CI] 3.50–3.78), diabetes (SHR 2.37, 95% CI 2.33–2.42) and low eGFR (Reference eGFR ≥ 90, eGFR 45–59: SHR 2.05, 95% CI 2.00–2.10) were the strongest predictors of hyperkalaemia. High BMI, South Asian ethnic group and smoking were also associated with an increased risk of hyperkalaemia, while female sex was associated with a reduced risk (Table 2).

**TABLE 2:** STRATIFY prediction models for Hyperkalaemia and Hyponatraemia. Values are sub-distribution hazard ratios and 95% confidence intervals (CPRD Gold)

	STRATIFY-Hyperkalaemia model			STRATIFY-Hyponatraemia model		
	SHR	95% CI	Association^b^	SHR	95% CI	Association
Age (transformed)^a^	1·005	1·004 to 1·005	↑	1·022	1·022 to 1·023	↑
Sex (Female)	0·859	0·849 to 0·870	↓	1·078	1·065 to 1·092	↑
BMI (ref. Normal)						
Underweight	0·975	0·923 to 1·030	−	1·062	1·025 to 1·101	↑
Overweight	1·051	1·034 to 1·068	↑	0·864	0·852 to 0·876	↓
Obese	1·129	1·109 to 1·150	↑	0·806	0·793 to 0·819	↓
Morbidly obese	1·354	1·304 to 1·407	↑	0·851	0·821 to 0·881	↓
Deprivation (ref. IMD=1, least deprived)						
IMD = 2	··	··	··	1·025	1·009 to 1·042	↑
IMD = 3	··	··	··	1·033	1·017 to 1·048	↑
IMD = 4	··	··	··	1·078	1·061 to 1·095	↑
IMD = 5	··	··	··	1·116	1·098 to 1·134	↑
Ethnicity (ref. White)						
Black	0·936	0·790 to 1·108	−	0·556	0·444 to 0·696	↓
South Asian	1·752	1·612 to 1·904	↑↑	1·220	1·078 to 1·380	↑
Smoking status (ref. Nonsmoker)						
Ex-smoker	1·191	1·173 to 1·210	↑	1·073	1·059 to 1·087	↑
Smoker	1·392	1·370 to 1·414	↑	1·348	1·327 to 1·369	↑
Alcohol (ref. Nondrinker)						
Light drinker	··	··	··	1·042	1·023 to 1·061	↑
Moderate drinker	··	··	··	1·124	1·098 to 1·150	↑
Heavy drinker	··	··	··	1·639	1·580 to 1·700	↑↑
Drinker, units not reported	··	··	··	1·025	1·005 to 1·041	↑
eGFR (ref. Normal, CKD stage 1)						
eGFR 60–89 (CKD stage 2)	1·428	1·401 to 1·456	↑	0·989	0·974 to 1·003	−
eGFR 45–59 (CKD stage 3a)	2 051	2·000 to 2·103	↑↑↑	0·937	0·915 to 0·960	↓
eGFR 30–44 (CKD stage 3b)	2 513	2·419 to 2·610	↑↑↑	0·799	0·776 to 0·823	↓
eGFR 15–29 (CKD stage 4)	3·088	2·906 to 3·282	↑↑↑↑	0·685	0·646 to 0·727	↓
eGFR <15 (CKD stage 5)	2·662	2·369 to 2·990	↑↑↑	0·809	0·712 to 0·919	↓
Systolic BP	1·004	1·004 to 1·005	↑	1·009	1·009 to 1·009	↑
eFI (transformed)^a^	1·129	1·115 to 1·143	↑	1·176	1·167 to 1·186	↑
Previous hyperkalaemia	3·638	3·501 to 3·781	↑↑↑↑	··	··	··
Previous hyponatraemia	··	··	··	4·945	4·844 to 5·049	↑↑↑↑↑
Heart failure	0·912	0·881 to 0·944	↓	0·900	0·872 to 0·929	↓
Diabetes	2·373	2·329 to 2·418	↑↑↑	2·084	2·048 to 2·120	↑↑↑
Cerebrovascular disease	0·891	0·868 to 0·916	↓	0·846	0·828 to 0·864	↓
Coronary artery disease	1·124	1·104 to 1·144	↑	··	··	··
Peripheral vascular disease	1·106	1·072 to 1·141	↑	··	··	··
Chronic liver disease	··	··	··	1·813	1·711 to 1·921	↑↑
Antihypertensive drugs						
ACE inhibitors	1·450	1·419 to 1·482	↑	1·354	1·337 to 1·372	↑
Beta blockers	1·106	1·080 to 1·133	↑	1·210	1·195 to 1·226	↑
Calcium channel blockers	1·005	0·981 to 1·029	−	1·057	1·042 to 1·072	↑
Thiazides and thiazide-like diuretics	0·751	0·730 to 0·773	↓	1·470	1·449 to 1·492	↑
Angiotensin II receptor antagonists	1·272	1·131 to 1·315	↑	1·301	1·271 to 1·332	↑
Alpha blockers	1·025	0·984 to 1·067	−	1·079	1·051 to 1·109	↑
Other antihypertensives	1·153	1·084 to 1·227	↑	1·140	1·097 to 1·184	↑
Loop diuretics	0·913	0·884 to 0·944	↓	0·893	0·876 to 0·911	↓
Potassium sparing diuretics	1·061	1·016 to 1·107	↑	1·264	1·233 to 1·296	↑
Other drugs						
Antidepressant	··	··	··	1·066	1·048 to 1·084	↑
Anticonvulsants	··	··	··	1·868	1·818 to 1·920	↑↑
Antipsychotics	··	··	··	0·951	0·918 to 0·985	↓
Proton pump inhibitors	··	··	··	1·170	1·155 to 1·185	↑
NSAIDS	··	··	··	1·118	1·104 to 1·131	↑

ACE, Angiotensin converting enzyme; BMI, Body mass index; BP, blood pressure; CKD, Chronic kidney disease; eFI, electronic frailty index; eGFR, estimated glomerular filtration rate; IMD, Indicies of multiple deprivation; NSAIDS, Nonsteroidal anti-inflammatory drugs.

a Variable transformed to account for nonlinear association with the outcome: age_transformed = (age/10) ^2– 37·03; eFI_transformed = eFI/0·1.

b Arrows to visualise the strength and direction of the association: ↑: 1<SHR<1·5 or 0·5<SHR<1; ↑↑: 1·5<SHR<2 or 0<SHR<0·5; ↑↑↑: 2 <SHR <3; ↑↑↑↑: 3 <SHR <4; ↑↑↑↑↑: SHR>4.

Prescription of RAAS medications was associated with an increased risk of hyperkalaemia: ACE inhibitors (SHR 1.45, 95% CI 1.42–1.48) and ARBs (SHR 1.27, 95% CI 1.13–1.32). Conversely, thiazides and thiazide-like diuretics (SHR 0.75, 95% CI 0.73–0.77) and loop diuretics (SHR 0.91, 95% CI 0.88–0.94) were all associated with a reduced risk of hyperkalaemia. Internal validation suggested minor miscalibration, however, recalibration was not necessary (Supplementary appendix 3 Figure S2).

#### STRATIFY-Hyponatraemia model

All 29 predictors considered in the model development were included in the final STRATIFY-Hyponatraemia model. Previous hyponatraemia (SHR 4.95, 95% CI 4.84–5.05), diabetes (SHR 2.08, 95% CI 2.05–2.12) and chronic liver disease (SHR 1.81, 95% CI 1.71–1.92) were the strongest predictors of hyponatraemia. High deprivation, South Asian ethnic group, drinking and smoking were associated with an increased risk of hyponatraemia. On the contrary, low eGFR, high BMI and black ethnic group were associated with a reduced risk of hyponatraemia (Table 2).

All types of antihypertensive medications, with the exception of loop diuretics, were associated with an increased the risk of hyponatraemia, with thiazides and thiazide-like diuretics (SHR 1.47, 95% CI 1.45–1.49), ACE inhibitors (SHR 1.35, 95% CI 1.34–1.37) and ARBs (SHR: 1.30, 95% CI 1.27–1.33) conferring the highest risks (Table 2). Anticonvulsants was also a strong predictor of hyponatraemia that associated with an increased risk (SHR 1.87, 95% CI 1.82–1.92). Internal validation also suggested minor miscalibration, again, recalibration was not necessary.

### External model validation

#### Predictive performance

The STRATIFY-Hyperkalaemia model exhibited very good calibration at all timepoints, with minor miscalibration in very few patients (O/E ratio at 1 year 1.04, 95% CI 1.01–1.08; 5 years 1.10, 95% CI 1.07–1.13; 10 years 1.16, 95% CI 1.13–1.19; Table 3, Fig. 1 ). Discrimination was also good at all timepoints (C-statistic at 1 year 0.73, 95% CI 0.73–0.74; 5 years 0.70, 95% CI 0.70–0.70; 10 years 0.69, 95% CI 0.69–0.69; Table 3). The STRATIFY-Hyponatraemia model also showed very good calibration at all timepoints, with minor over-prediction in very few patients at the highest risk (O/E ratio at 1 year 0.86, 95% CI 0.84–0.88; 5 years 0.93, 95% CI 0.91–0.95; 10 years 1.00, 95% CI 0.98–1.02 Table 3, Fig. 1 ). Overall, the STRATIFY-Hyponatraemia model showed very good discrimination (C-statistic at 1 year 0.85, 95% CI 0.84–0.85; 5 years 0.81, 95% CI 0.81–0.81; 10 years 0.80, 95% CI 0·80–0.80; Table 3). The performance of each model varied more among smaller practices, with more consistent performance seen as practice size increased (Supplementary appendix 3 Figures S3-S5).

**TABLE 3:** Predictive performance statistics at 1, 5, and 10 years for the Hyperkalaemia and Hyponatraemia prediction models upon external validation in CPRD Aurum

	STRATIFY-Hyperkalaemia model			STRATIFY-Hyponatraemia model		
	1 year	5 years	10 years	1 year	5 years	10 years
Observed/Expected^a^ – Fine-Gray model						
Pooled effect size (95% CI)	1·04 (1.006 to 1·075)	1·095 (1.065 to 1·127)	1·159 (1.128 to 1·19)	0·862 (0.841 to 0·884)	0·927 (0.909 to 0·946)	1·000 (0.983 to 1·017)
Prediction interval	0·427 to 2·533	0·516 to 2·324	0·564 to 2·379	0·445 to 1·669	0·549 to 1·566	0·638 to 1·566
Tau^2^	0·205 (0.185 to 0·228)	0·147 (0.132 to 0·163)	0·134 (0.121 to 0·149)	0·113 (0.102 to 0·126)	0·071 (0.064 to 0·079)	0·052 (0.047 to 0.058)
C statistic^b^						
Pooled effect size (95% CI)	0·732 (0.729 to 0·735)	0·701 (0.698 to 0·703)	0·687 (0.685 to 0·689)	0·846 (0.844 to 0·847)	0·813 (0.811 to 0·814)	0·799 (0.798 to 0·801)
Prediction interval	0·665 to 0·789	0·64 to 0·755	0·63 to 0·739	0·804 to 0·88	0·77 to 0·849	0·757 to 0·836
Tau^2^ (95% CI)	0·026 (0.022 to 0·031)	0·020 (0.017 to 0·022)	0·017 (0.015 to 0·019)	0·022 (0.019 to 0·026)	0·018 (0.015 to 0·020)	0·016 (0.014 to 0·018)
D statistic						
Pooled effect size (95% CI)	1·837 (1.811 to 1·863)	1·357 (1.339 to 1·374)	1·117 (1.101 to 1·133)	2·287 (2.263 to 2·31)	1·622 (1.602 to 1·641)	1·376 (1.358 to 1·395)
Prediction interval	1·34 to 2·334	0·971 to 1·742	0·749 to 1·486	1·79 to 2·783	1·162 to 2·081	0·915 to 1·838
Tau^2^ (95% CI)	0·064 (0.052 to 0·077)	0·039 (0.033 to 0·045)	0·035 (0.031 to 0·040)	0·064 (0.055 to 0·075)	0·055 (0.048 to 0·062)	0·055 (0.049 to 0·062)
Royston and Sauerbrei's *R*^2^_*D*_						
Median (LQ to UQ)	0·45 (0.381 to 0·513)	0·304 (0.257 to 0·357)	0·228 (0.186 to 0·274)	0·558 (0.512 to 0·607)	0·376 (0.339 to 0·426)	0·298 (0.263 to 0·345)
Mean (SD)	0·443 (0.107)	0·31 (0.081)	0·235 (0.076)	0·556 (0.076)	0·388 (0.075)	0·313 (0.078)

a Pooled on natural log scale.

b Pooled on logit scale.

**FIGURE 1:**
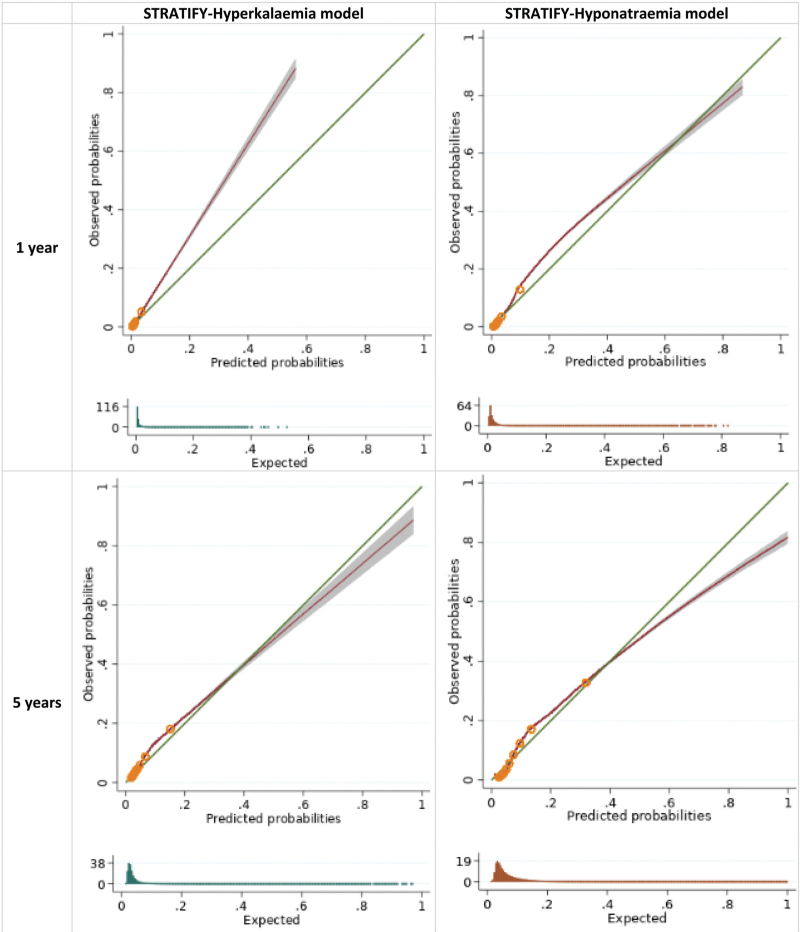
Calibration curves for the STRATIFY models upon external validation in CPRD Aurum. Groups represent tenths of the linear predictor, as created between deciles. Histograms show the distribution of predicted probabilities.

**FIGURE 1 (Continued):**
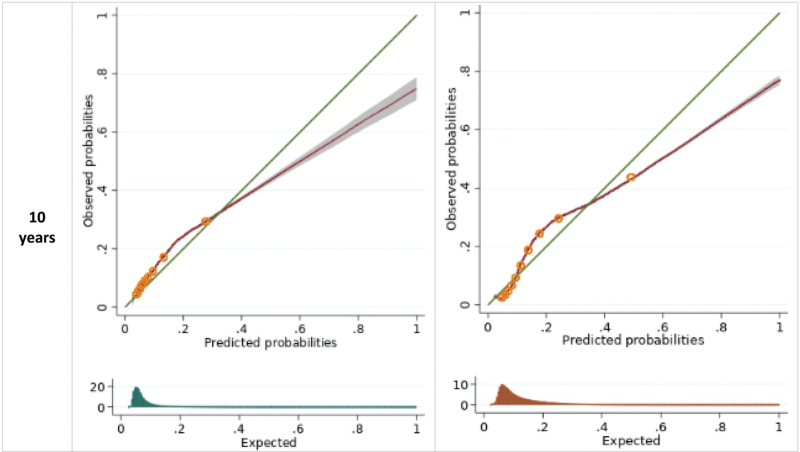
Calibration curves for the STRATIFY models upon external validation in CPRD Aurum. Groups represent tenths of the linear predictor, as created between deciles. Histograms show the distribution of predicted probabilities.

#### Clinical utility

**FIGURE 2:**
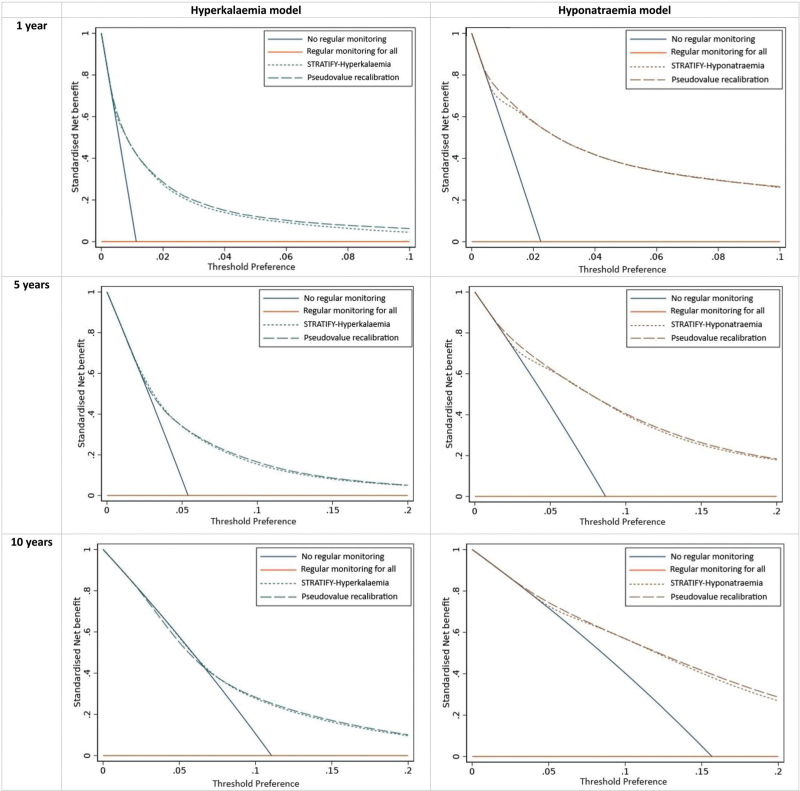
Decision curves, showing the standardized net benefit of using STRAFIFY prediction models across different threshold probabilities for assigning regular serum electrolytes monitoring.

Decision curve analysis indicated that both models had clinical utility across all three time points (Fig. 2). Using both models to guide serum electrolytes monitoring strategies would result in a higher net benefit compared to a “regular monitoring for all” approach. When compared to a “no regular monitoring” approach, both models are preferable for a wide range of risk thresholds.

Comparing the risks of electrolyte abnormalities with the risk of cardiovascular disease in CPRD GOLD using a 10% risk threshold for both (Supplementary appendix 3 Figure S6), the majority of patients with a low risk of cardiovascular disease also had a low risk of hyperkalaemia (46%) or hyponatraemia (40.2%). A small percentage of patients with a low risk of cardiovascular disease exhibited a high risk of adverse events (hyperkalaemia: 2%; hyponatraemia: 7.8%). In addition, most patients with a high risk of cardiovascular disease also had a high risk of hyponatraemia (44.1%), while a relative smaller proportion (24.2%) for hyperkalaemia. There was notable overlap across models, with nearly a quarter of patients being at high risk of hyperkalaemia, hyponatraemia and cardiovascular disease. Nonetheless, almost 40% of patients were not at a high risk for any of these three risks (Supplementary appendix 3 Figure S7).

## DISCUSSION

### Summary of main findings

This study developed two clinical prediction models for the risk of hyperkalaemia and hyponatraemia, in patients indicated for antihypertensive treatment. Both models calibrated well, showed good discrimination upon external validation, and exhibited clinical utility at almost any chosen risk threshold. Over 44 and 24% of patients were found to be at a high risk of both cardiovascular disease and hyponatraemia or hyperkalaemia, respectively, while 40% of patients were not at a high risk of either electrolyte abnormalities or CVD. Therefore, these models may be most useful in assisting clinicians when prescribing antihypertensive medications, helping to either avoid starting/continuing medications that are known to cause specific electrolyte abnormalities, ensure closer regular monitoring after initiating medications for those at a higher risk and reduce unnecessary testing in those with low predicted risks.

### Strengths and limitations

In this study, the modelling approach took into account the competing risk of death from other causes not related to the outcomes of interest. This consideration is particularly important when using the model in older patients with multiple health conditions or risk factors, as failure to account for competing risks can lead to overestimating an individual's benefit from antihypertensive treatment [33].

The prevalence of hyperkalaemia and hyponatraemia varies significantly depending on the definition of event and the healthcare setting [13]. We defined the outcomes of interest as a combination of diagnostic codes and blood test results within 2 years of the index date, using the diagnostic criteria set out in NICE guidelines; potassium threshold of more than 5.5 mmol/l for hyperkalaemia and sodium threshold of less than 135 mEq/l for hyponatraemia [8,11]. However, laboratories analysing these samples adopt different reference standards such as normal potassium between 3.5–5 and 3.5–5.3 mmol/l, reflecting changes in guidelines over the years. Using these reference ranges would have been advantageous, as they were used by clinicians to interpret the test results, but would have also resulted in a much less consistent definition.

We encountered a large amount of missing data for some predictor variables, especially ethnicity and eGFR, but this was addressed through the use of multiple imputation [26]. In addition, this study included predefined variables based on the literature and expert opinion, but it is possible that some important predictors were not included, which could impact the model performance. In our study, serum electrolytes were measured in over 95% of individuals in CPRD GOLD and 80% in CPRD Aurum during a 10-year follow-up. A minor limitation is the potential impact of missed measurements over time, and more frequent testing (i.e. every 3–6 months for all, if available) would have on the results.

### Comparison with previous literature

There are very few existing prediction models developed for estimating the risks of electrolyte abnormalities. We found no prediction model for hyponatraemia for use in a primary care setting. Of the few prediction models developed for hyperkalaemia, almost all were designed specifically for haemodialysis patients and patients with advanced chronic kidney disease. Only one relevant model for hyperkalaemia was identified. This study was designed for new users of ACE inhibitors/angiotensin II receptor blockers, and used logistic regression to predict hyperkalaemia events within the first year, and demonstrated a c-index of 0.818 (95% CI 0.794–0.841) in external validation [34]. However, this study only included patients with potassium tested at baseline and excluded patients (24%) who died or who did not have their potassium checked in the first year. This sample selection and attrition bias may have led to overfitting the prediction model.

In the present study, we developed clinical prediction models for hyperkalaemia and hyponatraemia events within 1, 5 and 10 years, accounting for the competing risk of death from other causes. Notably, these are the first survival prediction models we are aware of to examine an individual's overall risk of these two electrolyte abnormalities within both short and long periods using time-to-event analysis and competing risks modelling. Previously, we have developed clinical prediction models for other adverse events associated with antihypertensive treatment, including hypotension, syncope, falls, fracture and acute kidney injury [35–38]. The present models showed similar predictive performance, with better calibration, particularly in those with higher predicted risks.

### Implications for clinical practice

Various classes of antihypertensive medications are used for blood pressure management, with ACE inhibitors and angiotensin II receptor blockers, thiazide and thiazide-like diuretics, and calcium channel blockers being the most commonly recommended options [39–41]. Our risk prediction models provide individualized estimates of the risk of developing hyperkalaemia and hyponatraemia, adverse events which are commonly associated with specific antihypertensive drug classes. These models could therefore be useful in clinical decision making regarding which antihypertensive medication class to prescribe. For example, for patients at a high risk of hyperkalaemia but low-average risk of hyponatremia initiating or switching to a thiazide-type diuretic rather than ACE inhibitors/angiotensin II receptor blockers is recommended. Moreover, clinical guidelines for the management of hypertension often recommend combination therapy where monotherapy and lifestyle modifications fail to achieve adequate blood pressure control [40]. In such cases, to reduce potential harm, prescribing combination therapy, such as adding loop diuretics to current prescription in patients with advanced chronic kidney disease (avoiding RAAS medications) may also be effective [42,43].

Current clinical guidelines recommend monitoring serum electrolytes 1–2 weeks after initiating an RAAS medication, after each increase in dose, and regularly throughout treatment. Similarly, it is advised to measure serum electrolytes before starting a thiazide-type diuretics treatment and regular throughout treatment [44]. In our study, over 44 and 24% of patients with a high risk of CVD also had a high risk of hyponatraemia or hyperkalaemia, respectively. Nearly 40% of patients were not at a high risk of CVD or electrolyte abnormalities. The present clinical prediction models could help target closer monitoring of serum electrolytes for individuals at a higher risk of electrolyte abnormalities after initiating treatment, where resources are limited.

## CONCLUSION

The present study used two large datasets of electronic health records from the UK to derive and externally validate two clinical prediction models for common electrolyte abnormalities associated with antihypertensive therapy. These models demonstrated good performance upon external validation and could be used to support decision making to identify individuals for whom closer monitoring is recommended and which antihypertensive drug class to avoid.

## ACKNOWLEDGEMENTS

The authors thank Lucy Curtin for administrative support throughout the project. The Hospital Episode Statistics data used in this analysis are re-used with permission from NHS Digital who retain the copyright for those data. They also thank the Office for National Statistics for providing data on mortality. The Office for National Statistics, and NHS Digital bear no responsibility for the analysis or interpretation of the data. Finally, the authors are very grateful to all those patients who permit their anonymized routine NHS data to be used for this approved research.

J.P.S. conceived the project and wrote the protocol with F.D.R.H., R.J.M., R.S., R.D.R. A.W. and C.K. extracted data for analysis. A.W. developed the model under supervision of J.S. and C.K.; F.D.R.H., C.E.C., R.J.M. and R.P. advised on model development. L.A. validated the model under supervision of R.D.R. and K.I.E.S. A.W. wrote the first draft of the manuscript. All authors revised the manuscript and approved the final version. J.P.S. is the guarantor for this work and accepts full responsibility for the conduct of the study, had access to the data and controlled the decision to publish. The corresponding author (J.P.S.) attests that all listed authors meet authorship criteria and that no others meeting the criteria have been omitted.

This research, A.W., J.P.S. and C.K. were funded in whole, or in part, by the Wellcome Trust/Royal Society via a Sir Henry Dale Fellowship held by J.P.S. (ref: 211182/Z/18/Z) and the National Institute for Health Research (NIHR) School for Primary Care (project 430). L.A., K.I.E.S. and R.R. are supported by funding from the NIHR Birmingham Biomedical Research Centre at the University Hospitals Birmingham NHS Foundation Trust and the University of Birmingham. C.E.C. is part supported by a NIHR School for Primary Care grant (project 580). R.JMcM. is supported by an NIHR Senior Investigator award and by NIHR ARC Oxford Thames Valley. F.D.R.H. acknowledges part support from the NIHR ARC Oxford Thames Valley, and the NIHR Oxford OUH BRC. KIES is funded by an NIHR SPCR Launching Fellowship. A.B. has received research funding from Astra-Zeneca, NIHR, BMA Medical Research Foundation and UKRI. R.A.P. receives funding from the NIHR. A.C. is part funded by NIHR ARC Yorkshire & Humber and Health Data Research UK, an initiative funded by UK Research and Innovation Councils, National Institute for Health Research and the UK devolved administrations, and leading medical research charities. The views expressed are those of the author(s) and not necessarily those of the NHS, the NIHR or the Department of Health and Social Care. For the purpose of Open Access, the author has applied a CC BY public copyright licence to any Author Accepted Manuscript version arising from this submission.

The sponsor and funders had no role in the design and conduct of the study; collection, management, analysis and interpretation of the data; preparation, review or approval of the manuscript; and decision to submit the manuscript for publication.

The study protocol was approved by CPRD's Independent Scientific Advisory Committee in February 2019 before obtaining the data relevant to the project (protocol given in the Appendix in the Supplement). All data are fully anonymized so consent was not required. A project summary is published on the CPRD website (https://www.cprd.com/isac).

Data were obtained via a CPRD institutional licence. Requests for data sharing should be made directly to the CPRD. The algorithm is freely available for research use and can be downloaded from https://process.innovation.ox.ac.uk/software. Code lists used to define variables included in the dataset are available at https://github.com/jamessheppard48/STRATIFY-BP.

### Conflicts of interest

All authors have completed the ICMJE uniform disclosure form at www.icmje.org/coi_disclosure.pdf and declare: authors had financial support from the Wellcome Trust, Royal Society and National Institute for Health Research for the submitted work; no financial relationships with any organizations that might have an interest in the submitted work in the previous 3 years; no other relationships or activities that could appear to have influenced the submitted work.

The STRATIFY project is supported by the Wellcome Trust/Royal Society via a Sir Henry Dale Fellowship held by J.P.S. (ref: 211182/Z/18/Z) and the National Institute for Health Research (NIHR) School for Primary Care (project 430)

## Supplementary Material

Supplemental Digital Content
