# Supplementary material for: Developing prediction models for electrolyte abnormalities in patients indicated for antihypertensive therapy: evidence-based treatment and monitoring recommendations
Source: J Hypertens. 2025 May 7;43(8):1348–59. doi: 10.1097/HJH.0000000000004032 (PMC12237109; doi:10.1097/HJH.0000000000004032)
Supplement: Supplemental Digital Content [file jhype-43-1348-s001.docx]

**Supplementary Appendixes**

**Developing prediction models for electrolyte abnormalities in patients indicated for antihypertensive therapy: evidence-based treatment and monitoring recommendations**

Ariel Wang, *PhD,*^1^ Constantinos Koshiaris*, PhD,* ^1, 2^ Lucinda Archer, *PhD,*^3^ Richard D Riley, *PhD*,^3^ Kym IE Snell, *PhD,*^3^ Richard Stevens, *PhD,*^1^ Amitava Banerjee, *PhD*,^4^ Juliet A Usher-Smith, *PhD,*^5^ Subhashisa Swain, *PhD,*^1^ Andrew Clegg, *PhD,*^6^ Christopher E Clark, *PhD*,^7^ Rupert A Payne, *MRCGP*,^7^ FD Richard Hobbs, *FMedSci,*^1^ Richard J McManus*, PhD,*^1^ James P Sheppard, *PhD*,^1^ on behalf of the STRATIFY investigators*

*The STRAtifying Treatments In the multi-morbid Frail elderlY (STRATIFY) investigators include the authors and the following:

Simon Griffin, professor of primary care, Department of Public Health and Primary Care, Primary Care Unit, University of Cambridge; and Margaret Ogden, Patient and Public Involvement advisor.

^1^ Nuffield Department of Primary Care Health Sciences, University of Oxford, Oxford, UK

^2^ Department of Primary Care and Population Health, University of Nicosia Medical School, Engomi, CY-2414, Nicosia, Cyprus

^3^ National Institute for Health and Care Research (NIHR) Birmingham Biomedical Research Centre, Birmingham, UK

^4^ Institute of Health Informatics, University College London, London, UK

^5^ Primary Care Unit, Department of Public Health and Primary Care, University of Cambridge, UK

^6^ Academic Unit for Ageing and Stroke Research, Bradford Institute for Health Research, University of Leeds, UK

^7^ Exeter Collaboration for Academic Primary Care, University of Exeter Medical School, St Luke’s Campus, Magdalen Rd, Exeter, EX1 2LU, UK

**Corresponding author:** James P Sheppard

**Email:** james.sheppard@phc.ox.ac.uk

**Telephone:** +44 1865 617192

**Address:** Nuffield Department of Primary Care Health Sciences, Radcliffe Primary Care Building, Radcliffe Observatory Quarter, University of Oxford, Oxford, OX2 6GG, UK

[Appendix 1. Extended methods 5](#_Toc179961239)

[*Population* 5](#_Toc179961240)

[*Outcomes* 5](#_Toc179961241)

[*Model covariates* 6](#_Toc179961242)

[*Sample size* 6](#_Toc179961243)

[*Statistical analysis* 6](#_Toc179961244)

[Missing data 6](#_Toc179961245)

[Model development 7](#_Toc179961246)

[Apparent validation using development data 7](#_Toc179961247)

[External validation 8](#_Toc179961248)

[Clinical utility analysis 8](#_Toc179961249)

[*Patient and Public Involvement* 8](#_Toc179961250)

[Appendix 2. TRIPOD Checklist 12](#_Toc179961251)

[Appendix 3. Supplementary Tables & Figures 14](#_Toc179961252)

[Figure S1. Flow of study participants 14](#_Toc179961253)

[Table S1. Clinical codes used to define outcomes in each model 15](#_Toc179961254)

[Table S2. List of pre-specified predictors in each model 15](#_Toc179961255)

[Table S3. Baseline characteristics – External validation data (CPRD Aurum) 18](#_Toc179961256)

[Figure S2: Calibration curves (recalibration) for the apparent performance of the final STRATIFY models in CPRD GOLD. Groups represent tenths of the linear predictor, as created between deciles. Histograms show the distribution of predicted probabilities. 20](#_Toc179961257)

[Figure S3: Performance variability of the STRATIFY Hyperkalaemia model on external validation across GP practices, with panels (A) Observed/Expected, (B) $\boldsymbol{RD}\mathbf{2}$, (C) D-statistic, and (D) C-statistic 21](#_Toc179961258)

[Figure S4: Performance variability of the STRATIFY Hyponatraemia model on external validation across GP practices, with panels (A) Observed/Expected, (B) $\boldsymbol{RD}\mathbf{2}$, (C) D-statistic, and (D) C-statistic 22](#_Toc179961259)

[Figure S5. Scatter plot showing the number of events (by outcome time point), against total practice size for practices in the external validation data. 23](#_Toc179961260)

[Figure S6. Comparison of 10-year cardiovascular risk (QRisk2) and final STRATIFY-Hyperkalaemia and STRATIFY-Hyponatraemia risk in the CPRD GOLD dataset, using 10% risk threshold 24](#_Toc179961261)

[Figure S7. Venn diagrams showing the overlap of high-risk patients (≥10%) as classified using the final STRATIFY-Hyperkalaemia and STRATIFY-Hyponatraemia, as compared with Qrisk2 CVD risks at 10 years in the CPRD Gold cohort (derivation) 25](#_Toc179961262)

[Figure S8. Distribution of linear predictor, grouped by outcome type at 10 years 26](#_Toc179961263)

[Appendix 4. Supplementary Equations 27](#_Toc179961264)

[Appendix 5. Protocol 29](#_Toc179961265)

#

# Appendix 1. Extended methods

*Design*

This study used a retrospective observational cohort design using routine primary care data from the Clinical Practice Research Datalink (CPRD) in the UK. Patient-level linkage to Office for National Statistics (ONS) Death Registration Data, Hospital Episode Statistics (HES), and Index of Multiple Deprivation (IMD) data was conducted. CPRD includes anonymised patient electronic health records from general practice (GP) surgeries using the Vision (Cegedim Healthcare Solutions, London, England) or EMIS (EMIS, Leeds, England) software systems. These two systems provide two separate primary care databases: CPRD GOLD (using the Vision software) and CPRD Aurum (using EMIS software). In this study, we used CPRD GOLD to derive the two prediction models and CPRD Aurum to externally validate them. CPRD GOLD comprises 11·3 million patients (4·4 million currently alive) from 674 practices, while CPRD Aurum comprises 19 million patients (7 million currently alive) from 738 practices.^1,2^ Both datasets are representative of the UK population in terms of age, sex, and ethnicity. The protocol for this study was approved by the CPRD Independent Scientific Advisory Committee (ISAC) (protocol number 19_042, see appendix 5).

## *Population*

Participants aged≥40 years registered to a CPRD practice between 1^st^ January 1998 and 31^st^ December 2018 with at least a blood pressure measurement between 130-179 mmHg were included in the study. The threshold of 130 mmHg was selected to accommodate various treatment initiation thresholds outlined in international hypertension guidelines.^3,4^ Patients with a baseline systolic blood pressure reading ≥180 mmHg were excluded from the cohort as antihypertensive treatment would be indicated for them regardless of the risk of adverse events, as per guidelines. All patient characteristics and predictors for the models were determined at the index date, defined as 12 months after cohort entry. The same eligibility criteria and methods for determining characteristics were applied to both the development and validation cohorts.

Patients exited the cohort on the study end date (31^st^ December 2018), or upon transferring out of a registered CPRD practice, died, or experienced the specific outcomes of interest. The study follow-up period was up to 10 years. To prevent patient duplications, records from the CPRD Aurum (validation cohort) were excluded if they were also present in the CPRD GOLD (development cohort) dataset due to switching electronic health record systems during the study period.

## *Outcomes*

The primary outcomes were first hyperkalaemia or hyponatraemia event within 10 years of index date. Hyperkalaemia was defined using a combination of test result (serum potassium>5·5 mmol/L) or diagnosis codes of hyperkalaemia in CPRD (READ code), HES and ONS (ICD-10 code) within 10 years of the index date. Similarly, hyponatraemia was also defined using a combination of test result (serum sodium <135 mEq/L) or diagnosis codes of hyponatraemia in CPRD, HES and ONS within the same time frame. READ codes are a coded thesaurus of clinical terms. They have been used in the National Health Service (NHS) since 1985 to provide a standard vocabulary for clinicians to record patient findings and procedures in both primary and secondary care.^5^ Pre-specified secondary outcomes were hyperkalaemia or hyponatraemia (defined in the same way) within 1 and 5 years of the index date. (Clinical codes for outcomes, see appendix 3 Table S1).

## *Model covariates*

Clinically relevant predictors of hyperkalaemia and hyponatraemia were identified through literature searches, researcher consultation and expert clinical opinion. These predictors are summarised in appendix 3 Table S2. A total of 25 predictors were considered for the hyperkalaemia model and 29 for the hyponatraemia model. Predictors included patient demographics (age, sex, body mass index [BMI], smoking status, alcohol consumption, and index of multiple deprivation [IMD]), baseline systolic blood pressure (SBP), frailty (electronic frailty index [eFI]), estimate glomerular filtration rate (eGFR), comorbidities (previous history of hyperkalaemia or hyponatraemia, diabetes, heart failure, cerebrovascular disease, coronary artery disease, peripheral vascular disease, chronic liver disease), and prescribed medications (including antihypertensives, antidepressants, anticonvulsants, antipsychotics, proton pump inhibitors [PPIs] and Non-steroidal anti-inflammatory drugs [NSAIDS]). Covariates were defined by any occurrence of relevant READ codes at any time point before the index date, with the exception of blood test results (serum creatinine, potassium and sodium) and previous history of hyperkalaemia and hyponatraemia which were captured in 2 years prior to the index date; and medication prescriptions including antihypertensives, which were defined as any prescription within 1 year prior to the index date.

The most recent serum creatinine measured within 2 years before the index date was used to calculate the eGFR, with implausible serum creatinine values (<2 and >1000 μmol/l) excluded. The 2021 CKD-EPI creatinine equation, which includes age, sex and creatinine was used to calculate eGFR, and eGFR categories were based on the NICE guideline [NG203].^6,7^

## *Sample size*

A sample size of approximately 16,778 patients was estimated to be required for the development of the risk equations. The sample size calculation for model development was based on an event rate of between 14·6 and 36 per 1,000 patient years of follow-up,^8,9^ an expected median follow up of 7 years,^10^ an estimate of Nagelkerke’s R^2^ statistic of 0·15, a global shrinkage factor of 0·9 and a maximum number of 40 parameters in the model.^11^ For external validation, a prognostic model requires ideally 200 or more events.^12^ The actual sample sizes in both development and validation cohorts far exceeded these estimates.

## *Statistical analysis*

Baseline characteristics were summarised using descriptive statistics in the development and validation cohorts separately. Mean and standard deviations (SD) or median and interquartile range (IQR) were reported for continuous variables, while frequencies and percentages were reported for binary/categorical variables.

### Missing data

Multiple imputation with chained equations was used to impute missing data. Ten imputations were generated for the development and validation datasets separately. The imputation models included all covariates within each dataset, along with the Nelson-Aalen estimator for the cumulative baseline cause-specific hazards for hyperkalaemia or hyponatraemia, and for the competing event of death, and binary event indicators for each of these possible event types.^13,14^ Where information was missing on the diagnosis of comorbidities or prescribed medications, it was assumed that no diagnosis or prescription was present. Predictor variables requiring imputation were ethnicity, BMI groups, deprivation score (validation cohort only), smoking status, alcohol consumption and eGFR categories.

Imputations were assessed for consistency by comparing density plots, histograms, and summary statistics across imputations. The model coefficients and predictive performance measures were then estimated in each imputed dataset separately, before being combined across imputations using Rubin’s Rules.^15^

### Model development

Each model was developed and internally validated by researchers at the University of Oxford (AW, CK, JPS). Multivariable prediction models were fitted in each imputed dataset using a Fine-Gray sub-distribution hazard model, taking into account the competing risk of death by other causes.^16^ These accounted for the competing risk to avoid overestimation of the predicted probabilities of each adverse event.^17^ Predictor effects in the model are reported as sub-distribution hazard ratios (SHR) with 95% confidence intervals, and the post-estimation baseline cumulative incidence for each event was estimated using a Breslow-type estimator as defined in the Fine-Gray paper.^16^ Analyses were undertaken using the *fastcmprsk* package in RStudio.^18^ Automated variable selection methods were not used, since all covariates were all predetermined based on the literature and expert opinion, and given the large sample size would result in nearly all predictors having a statistically significant association with the outcome. To ensure a parsimonious model, variables with no association in multivariable analysis were excluded before fitting the final models. Shrinkage or penalisation methods to adjust for overfitting were not necessary due to the very large sample size.

Fractional polynomials were used to examine the linearity assumption of all continuous variables (age, systolic blood pressure and frailty index) and identify the best fitting transformation.^19^ Fractional polynomials (FPs) were identified separately within each imputed dataset and the most consistent transformation across the imputations was selected, choosing lower order FP terms wherever possible for the sake of parsimony. The selected FP format for each continuous variable was then forced into each model for all imputations to ensure consistency in coefficient estimation.

### Apparent validation using development data

The model’s apparent validation was assessed using calibration plots comparing the observed to predicted risks at 1, 5 and 10 years. Observed outcome probabilities were defined using pseudo-values: jack-knife estimators representing an individual’s contribution to the cumulative incidence function for each event accounting for the competing risk of death and calculated by the Aalen–Johansen method.^20^ Pseudo-values were generated separately in 50 groups by linear predictor value, for stability, and accounted for the competing risk of death and non-informative right censoring.^21,22^ Calibration plots were produced using these pseudo-values and generated using a loess smoother calibration curve to show apparent calibration across the spectrum of predicted risks, with 95% confidence intervals. Plots were generated in each imputed dataset separately and were checked for consistency across imputations. Both models appeared to be well calibrated in the development cohort, without the need for any recalibration. Nonetheless, for completeness, recalibrations of each original model separately at 1, 5 and 10 years were also undertaken. Linear recalibration was undertaken at 1 year where non-linear recalibration using fractional polynomials was used for 5 and 10 years.

### External validation

The external validation of each prediction model was conducted by researchers at The University of Birmingham (LA, KIES, RDR), independent of the model development team. The prediction model algorithms (Appendix 4. Equations) were applied to each individual in the external validation cohort to give the predicted probabilities of experiencing a hyperkalaemia or hyponatraemia event within 1, 5 and 10 years, taking account of the competing risk of death by other causes.^23^ Model calibration was assessed through comparison of predicted probabilities to observed pseudo-values estimated using jack-knife estimators representing an individual’s contribution to the cumulative incidence function for adverse events, accounting for competing risks, and calculated by the Aalen–Johansen method, in the external validation cohort.^20^

Predictive performance was quantified by calculating the Observed to Expected ratio (O/E), Harrell’s C-statistic, and Royston’s D-statistic with its associated R^2^ statistic,^24^ each applied to the same pseudo-values as above, and using calibration plots. Heterogeneity in model performance across different GP practices was assessed using a random effects meta-analysis, using restricted maximum likelihood estimation (REML), given that the case mix and incidence of outcomes were expected to vary between practices.^25^ The O/E ratio was pooled across practices on the natural log scale, the C-statistic on the logit scale (with the standard errors of logit-C calculated using the delta method), and the D-statistic on its original scale.^26,27^ Pooled estimates are reported with prediction intervals (PI) to give an indication of expected model performance in a new GP practice.

### Clinical utility analysis

Clinical utility was assessed by plotting the 1, 5 and 10-year risk of hyperkalaemia or hyponatraemia against the 10-year risk of CVD, calculated using the QRisk2 algorithm using a 10% threshold.^28^ We used net benefit analysis to examine the benefits of using the STRATIFY prediction models for clinical decision making on serum electrolytes monitoring.^29^ The STRATIFY prediction models were compared with model blind methods of no regular monitoring (which may involve remove current guidelines on regular serum electrolytes checking) for all patients, or regular monitoring (starting or continuing) for all patients, regardless of risk. Venn diagrams were used to visualise the overlap between models in those with high-predicted risks (≥10%) as classified using the final STRATIFY-Hyperkalaemia and STRATIFY-Hyponatraemia models, compared with Qrisk2 CVD risks.

## *Patient and Public Involvement*

This study was developed and conducted with the help of our patient and public advisor Margaret Ogden. As a member of our study advisory group, she commented on the study protocol and was present in all team meetings discussing results and reporting. We also held a focus group with several older adults during the study to discuss broader issues related to drugs for cardiovascular disease prevention and adverse events, which informed the interpretation of this work.

**Reference:**

1. Herrett E, Gallagher AM, Bhaskaran K, et al. Data Resource Profile: Clinical Practice Research Datalink (CPRD). *International journal of epidemiology* 2015;44(3):827-36. doi: 10.1093/ije/dyv098 [published Online First: 2015/06/08]

2. Wolf A, Dedman D, Campbell J, et al. Data resource profile: Clinical Practice Research Datalink (CPRD) Aurum. *International journal of epidemiology* 2019;48(6):1740-40g. doi: 10.1093/ije/dyz034 [published Online First: 2019/03/13]

3. Mancia G, Kreutz R, Brunström M, et al. 2023 ESH Guidelines for the management of arterial hypertension The Task Force for the management of arterial hypertension of the European Society of Hypertension Endorsed by the European Renal Association (ERA) and the International Society of Hypertension (ISH). *J Hypertension* 2023 doi: 10.1097/hjh.0000000000003480 [published Online First: 2023/06/22]

4. Whelton PK, Carey RM, Aronow WS, et al. 2017 ACC/AHA/AAPA/ABC/ACPM/AGS/APhA/ASH/ASPC/NMA/PCNA Guideline for the Prevention, Detection, Evaluation, and Management of High Blood Pressure in Adults: A Report of the American College of Cardiology/American Heart Association Task Force on Clinical Practice Guidelines. *Hypertension (Dallas, Tex : 1979)* 2018;71(6):e13-e115. doi: 10.1161/hyp.0000000000000065 [published Online First: 2017/11/15]

5. NHS digital, https://digital.nhs.uk/services/terminology-and-classifications/read-codes

6. Inker LA, Eneanya ND, Coresh J, Tighiouart H, Wang D, Sang Y, et al. Chronic Kidney Disease Epidemiology Collaboration. New Creatinine- and Cystatin C-Based Equations to Estimate GFR without Race. *N Engl J Med*. 2021 Nov 4;385(19):1737-1749. doi: 10.1056/NEJMoa2102953. Epub 2021 Sep 23. PMID: 34554658; PMCID: PMC8822996.

7. NICE guideline [NG203]Chronic kidney disease: assessment and management, Published: 25 August 2021, Last updated: 24 November 2021, https://www.nice.org.uk/guidance/ng203

8. Nilsson E, Gasparini A, Ärnlöv J, et al. Incidence and determinants of hyperkalemia and hypokalemia in a large healthcare system. *Int J Cardiol.* 2017;245:277-284

9. Hawkins, R. C. (2003). Age and gender as risk factors for hyponatremia and hypernatremia. *Clinica chimica acta*, 337(1-2), 169-172.

10. Stevens SL, McManus RJ, Stevens RJ. The utility of long-term blood pressure variability for cardiovascular risk prediction in primary care. *J Hypertension* 2019;37(3):522-29. doi: 10.1097/hjh.0000000000001923 [published Online First: 2018/09/21]

11. Riley RD, Snell KI, Ensor J, et al. Minimum sample size for developing a multivariable prediction model: PART II - binary and time-to-event outcomes. *Stat Med* 2019;38(7):1276-96. doi: 10.1002/sim.7992 [published Online First: 2018/10/26]

12. Collins GS, Ogundimu EO, Altman DG. Sample size considerations for the external validation of a multivariable prognostic model: a resampling study. *Stat Med* 2016;35(2):214-26. doi: 10.1002/sim.6787 [published Online First: 2015/11/11]

13. White IR, Royston P. Imputing missing covariate values for the Cox model. *Statistics in Medicine* 2009;28(15):1982-98. doi: 10.1002/sim.3618 [doi]

14. Lau B, Leskoa C. Missingness in the Setting of Competing Risks: from missing values to missing potential outcomes. *Curr Epidemiol Rep* 2018;5(2):153–59.

15. Rubin DB. Multiple Imputation for Nonresponse in Surveys. New York: John Wiley & Sons 1987.

16. Fine JP, Gray RJ. A Proportional Hazards Model for the Subdistribution of a Competing Risk. *Journal of the American Statistical Association* 1999;94(446):496-509. doi: 10.1080/01621459.1999.10474144

17. Feakins BG, McFadden EC, Farmer AJ, et al. Standard and competing risk analysis of the effect of albuminuria on cardiovascular and cancer mortality in patients with type 2 diabetes mellitus. *Diagn Progn Res* 2018;2:13. doi: 10.1186/s41512-018-0035-4 [published Online First: 2019/05/17]

18. Kawaguchi ES, Shen J, Li G, et al. A Fast and Scalable Implementation Method for Competing Risks Data with the R Package fastcmprsk. *arXiv: Computation* 2019

19. Royston P, Ambler G, Sauerbrei W. The use of fractional polynomials to model continuous risk variables in epidemiology. *International journal of epidemiology* 1999;28(5):964-74. doi: 10.1093/ije/28.5.964 [published Online First: 1999/12/22]

20. Aalen OO, Johansen S. An Empirical Transition Matrix for Non-Homogeneous Markov Chains Based on Censored Observations. *Scandinavian Journal of Statistics* 1978;5(3):141-50.

21. Graw F, Gerds T, Schumacher M. On pseudo-values for regression analysis in competing risks models. *Lifetime Data Anal* 2009;15(2):241-55. doi: 10.1007/s10985-008-9107-z

22. Andersen P, Perme M. Pseudo-observations in survival analysis. *Stat Methods Med Res* 2010;19(1):71-99. doi: 10.1177/0962280209105020

23. Royston P. Tools for Checking Calibration of a Cox Model in External Validation: Approach Based on Individual Event Probabilities. *The Stata Journal* 2014;14(4):738-55. doi: 10.1177/1536867x1401400403

24. Rahman MS, Ambler G, Choodari-Oskooei B, et al. Review and evaluation of performance measures for survival prediction models in external validation settings. *BMC Med Res Methodol* 2017;17(1):60. doi: 10.1186/s12874-017-0336-2

25. Riley RD, Ensor J, Snell KI, et al. External validation of clinical prediction models using big datasets from e-health records or IPD meta-analysis: opportunities and challenges. *BMJ (Clinical research ed)* 2016;353:i3140. doi: 10.1136/bmj.i3140 [published Online First: 2016/06/24]

26. Snell KI, Ensor J, Debray TP, et al. Meta-analysis of prediction model performance across multiple studies: Which scale helps ensure between-study normality for the C-statistic and calibration measures? *Stat Methods Med Res* 2017:962280217705678. doi: 10.1177/0962280217705678

27. Debray TP, Damen JA, Riley RD, et al. A framework for meta-analysis of prediction model studies with binary and time-to-event outcomes. 2018;28(9):2768-86.

28. Hippisley-Cox J, Coupland C, Vinogradova Y, et al. Predicting cardiovascular risk in England and Wales: prospective derivation and validation of QRISK2. *BMJ (Clinical research ed)* 2008;336(7659):1475-82. doi: 10.1136/bmj.39609.449676.25 [published Online First: 2008/06/25]

29. Vickers AJ, Van Calster B, Steyerberg EW. Net benefit approaches to the evaluation of prediction models, molecular markers, and diagnostic tests. *BMJ (Clinical research ed)* 2016;352:i6 doi: <https://doi.org/10.1136/bmj.i6>

# Appendix 2. TRIPOD Checklist

| **Section/Topic** | **Item** |  | **Checklist Item** | **Page** |
| --- | --- | --- | --- | --- |
| **Title and abstract** | | | | |
| Title | 1 | D;V | Identify the study as developing and/or validating a multivariable prediction model, the target population, and the outcome to be predicted. | Page 1 |
| Abstract | 2 | D;V | Provide a summary of objectives, study design, setting, participants, sample size, predictors, outcome, statistical analysis, results, and conclusions. | Page 3-4 |
| **Introduction** | | | | |
| Background and objectives | 3a | D;V | Explain the medical context (including whether diagnostic or prognostic) and rationale for developing or validating the multivariable prediction model, including references to existing models. | Page 5 |
|  | 3b | D;V | Specify the objectives, including whether the study describes the development or validation of the model or both. | Page 6 |
| **Methods** | | | | |
| Source of data | 4a | D;V | Describe the study design or source of data (e.g., randomized trial, cohort, or registry data), separately for the development and validation data sets, if applicable. | Page 6-7, Appendix 1 extended method |
|  | 4b | D;V | Specify the key study dates, including start of accrual; end of accrual; and, if applicable, end of follow-up. | Page 7 |
| Participants | 5a | D;V | Specify key elements of the study setting (e.g., primary care, secondary care, general population) including number and location of centres. | Page 6-7 |
|  | 5b | D;V | Describe eligibility criteria for participants. | Page 7 |
|  | 5c | D;V | Give details of treatments received, if relevant. | Page 7 |
| Outcome | 6a | D;V | Clearly define the outcome that is predicted by the prediction model, including how and when assessed. | Page 7-8 |
|  | 6b | D;V | Report any actions to blind assessment of the outcome to be predicted. | n/a |
| Predictors | 7a | D;V | Clearly define all predictors used in developing or validating the multivariable prediction model, including how and when they were measured. | Pages 8, Appendix 1 extended method |
|  | 7b | D;V | Report any actions to blind assessment of predictors for the outcome and other predictors. | n/a |
| Sample size | 8 | D;V | Explain how the study size was arrived at. | Page 8 |
| Missing data | 9 | D;V | Describe how missing data were handled (e.g., complete-case analysis, single imputation, multiple imputation) with details of any imputation method. | Page 11 |
| Statistical analysis methods | 10a | D | Describe how predictors were handled in the analyses. | Page 9, Appendix 1 extended method |
|  | 10b | D | Specify type of model, all model-building procedures (including any predictor selection), and method for internal validation. | Page 9 |
|  | 10c | V | For validation, describe how the predictions were calculated. | Pages 9-10 |
|  | 10d | D;V | Specify all measures used to assess model performance and, if relevant, to compare multiple models. | Pages 9-10, Appendix 1 extended method |
|  | 10e | V | Describe any model updating (e.g., recalibration) arising from the validation, if done. | Page 10 |
| Risk groups | 11 | D;V | Provide details on how risk groups were created, if done. | Page 10-11 |
| Development vs. validation | 12 | V | For validation, identify any differences from the development data in setting, eligibility criteria, outcome, and predictors. | Pages 6-7, 10, Appendix 1 extended method |
| **Results** | | | | |
| Participants | 13a | D;V | Describe the flow of participants through the study, including the number of participants with and without the outcome and, if applicable, a summary of the follow-up time. A diagram may be helpful. | Pages 11-12, Table 1, Supplementary Figure S1 & Table S3 |
|  | 13b | D;V | Describe the characteristics of the participants (basic demographics, clinical features, available predictors), including the number of participants with missing data for predictors and outcome. | Pages 11-12, Table 1, Supplementary Table S3 |
|  | 13c | V | For validation, show a comparison with the development data of the distribution of important variables (demographics, predictors and outcome). | Pages 12, Table 1, Supplementary Table S3 |
| Model development | 14a | D | Specify the number of participants and outcome events in each analysis. | Pages 11-12, Table 1, Supplementary Table S3 |
|  | 14b | D | If done, report the unadjusted association between each candidate predictor and outcome. | n/a |
| Model specification | 15a | D | Present the full prediction model to allow predictions for individuals (i.e., all regression coefficients, and model intercept or baseline survival at a given time point). | Pages 12-13, Table 2 |
|  | 15b | D | Explain how to the use the prediction model. | Appendix 4. Supplementary Equations |
| Model performance | 16 | D;V | Report performance measures (with CIs) for the prediction model. | Page 14, Table 3 |
| Model-updating | 17 | V | If done, report the results from any model updating (i.e., model specification, model performance). | Figure2, Supplementary Figure S2 |
| **Discussion** | | | | |
| Limitations | 18 | D;V | Discuss any limitations of the study (such as nonrepresentative sample, few events per predictor, missing data). | Pape 16-17 |
| Interpretation | 19a | V | For validation, discuss the results with reference to performance in the development data, and any other validation data. | Pages 15, 17 |
|  | 19b | D;V | Give an overall interpretation of the results, considering objectives, limitations, results from similar studies, and other relevant evidence. | Pages 15-18 |
| Implications | 20 | D;V | Discuss the potential clinical use of the model and implications for future research. | Page 18-19 |
| **Other information** | | | | |
| Supplementary information | 21 | D;V | Provide information about the availability of supplementary resources, such as study protocol, Web calculator, and data sets. | Appendix |
| Funding | 22 | D;V | Give the source of funding and the role of the funders for the present study. | Page 20-21 |

*Items relevant only to the development of a prediction model are denoted by D, items relating solely to a validation of a prediction model are denoted by V, and items relating to both are denoted D;V. We recommend using the TRIPOD Checklist in conjunction with the TRIPOD Explanation and Elaboration document.

# Appendix 3. Supplementary Tables & Figures

## **Figure S1.** Flow of study participants


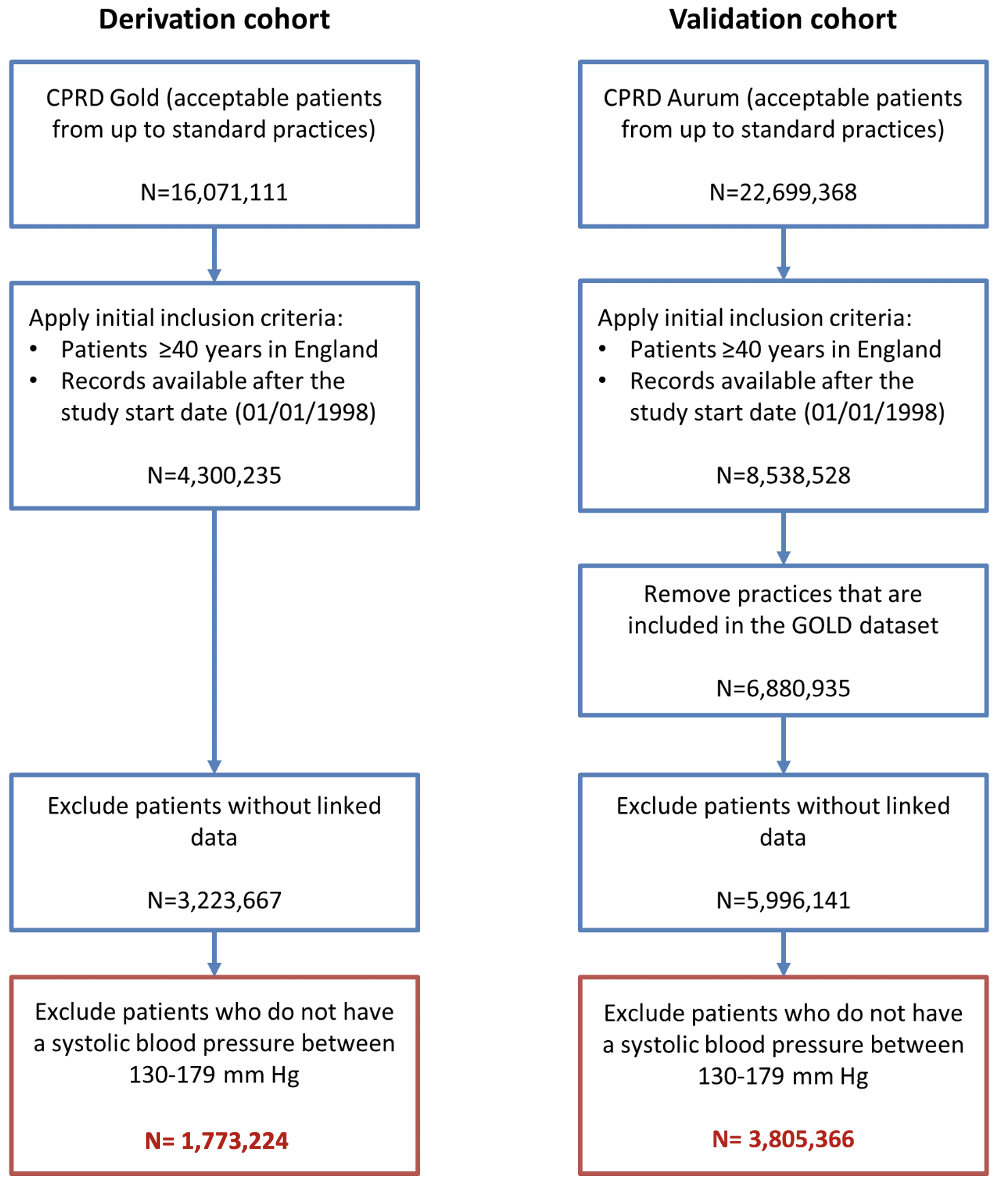


## **Table S1.** Clinical codes used to define outcomes in each model

| **Outcome** | **ICD10/READ code** | **Description** |
| --- | --- | --- |
| **Hyperkalaemia** | E87.5 | Hyperkalaemia |
|  | 44I4100 | Raised serum potassium level |
|  | C367.00 | Hyperkalaemia |
| **Hyponatraemia** | E87.1 | Hypo-osmolality and hyponatraemia |
|  | C361.00 | Hyposmolality and or hyponatraemia |
|  | C361.11 | Hyponatraemia |

## **Table S2.** List of pre-specified predictors in each model

| **Predictor** | **Model 1: Hyperkalaemia model** | **Model 2: Hyponatraemia model** | **Details** |
| --- | --- | --- | --- |
| Age | x^1,2^ | x^3-8^ |  |
| Sex | x^1,2^ | x^5,8^ |  |
| Body mass index | x | x^6^ |  |
| Ethnicity | x | x^4^ |  |
| Alcohol consumption | x | x | Included in previous models |
| Smoking status | x | x | Included in previous models |
| Index of Multiple Deprivation (IMD) | x | x | Included in previous models |
| Systolic blood pressure | x | x | Included in previous models |
| Electronic frailty score | x | x^7^ |  |
| eGFR in 2 years of the index date (calculated by creatinine test results) | x^1^ | x^4^ | Formula to calculate eGFR:  2021 CKD-EPI Creatinine^18^ |
| Previous Hyperkalaemia – assessed by potassium test result or a clinical diagnosis of hyperkalaemia (CPRD+HES) | x^11^ |  | serum potassium>5·5 mmol/L |
| Previous Hyponatraemia – assessed by sodium test result or a clinical diagnosis of hyponatraemia (CPRD+HES) |  | x | serum sodium <135 mEq/L |
| Heart Failure | x^1^ | x |  |
| Chronic Liver disease |  | x ^12,13^ |  |
| Diabetes | x^1,2,9^ | x^8^ |  |
| Coronary artery disease | x^9^ |  |  |
| Peripheral vascular disease | x^1,9^ |  |  |
| Cerebrovascular disease | x^11^ | x |  |
| ACE inhibitors | x^1,2^ | x |  |
| Angiotensin II receptor blockers | x^1^ | x |  |
| Alpha blockers |  |  | Included in previous models |
| Beta blockers | x^1^ |  |  |
| Calcium channel blockers |  |  | Included in previous models |
| Thiazide and thiazide-like Diuretics | x^1,2,11^ | x^3,5^ |  |
| Other antihypertensives (include centrally acting, vasodilators, renin inhibitors and aldosterone diuretics) | x^1^ | x | Included in previous models |
| Loop diuretics | x | x | Expert opinion |
| Potassium sparing diuretics | x ^1,10,11^ | x | Expert opinion |
| Antidepressants |  | x^3^ |  |
| Antipsychotics |  | x ^15^ |  |
| Anti-epileptic (Anticonvulsants) |  | x ^14,15,16^ |  |
| PPI |  | x | Expert opinion |
| NSAIDs |  | x | Expert opinion |

**References**

1. Nilsson E, Gasparini A, Ärnlöv J, et al. Incidence and determinants of hyperkalemia and hypokalemia in a large healthcare system. Int J Cardiol. 2017;245:277-284.

2. Desai AS, Swedberg K, McMurray JJ, et al. Incidence and predictors of hyperkalemia in patients with heart failure: an analysis of the CHARM Program. J Am Coll Cardiol. 2007;50(20):1959-1966.

3. Nwulu U, Chuchu M, McDowell S, Hodson J, Coleman JJ. Investigating risk factors associated with hyponatraemia in hospitalised patients using an electronic prescribing system with health records. E-Journal of the British Pharmacological Society. 2012;10(4):abst159P.

4. Matanes F, Siddiqui M, Dudenbostel T, Calhoun D, Oparil S. Abstract P187: Incidence and Predictors of Diuretic-Induced Hyponatremia. Hypertension (Dallas, Tex : 1979). 2019;74(Suppl_1):AP187-AP187.

5. Ravioli S, Bahmad S, Funk GC, Schwarz C, Exadaktylos A, Lindner G. Risk of Electrolyte Disorders, Syncope, and Falls in Patients Taking Thiazide Diuretics: Results of a Cross-Sectional Study. Am J Med. 2021;134(9):1148-1154.

6. Chow KM, Szeto CC, Wong TY, Leung CB, Li PK. Risk factors for thiazide-induced hyponatraemia. Qjm. 2003;96(12):911-917.

7. Makam AN, Boscardin WJ, Miao Y, Steinman MA. Risk of thiazide-induced metabolic adverse events in older adults. J Am Geriatr Soc. 2014;62(6):1039-1045.

8. Al Qahtani M, Alshahrani A, Alskaini A, et al. Prevalence of hyponatremia among patients who used indapamide and hydrochlorothiazide: a single center retrospective study. Saudi J Kidney Dis Transpl. 2013;24(2):281-285.

9. Jain N, Kotla S, Little BB, et al. Predictors of hyperkalemia and death in patients with cardiac and renal disease. Am J Cardiol. 2012;109(10):1510-1513.

10. Hunter RW, Bailey MA. Hyperkalemia: pathophysiology, risk factors and consequences. Nephrol Dial Transplant. 2019 Dec 1;34(Suppl 3):iii2-iii11. doi: 10.1093/ndt/gfz206. PMID: 31800080; PMCID: PMC6892421.

11. Bandak G, Sang Y, Gasparini A, Chang AR, Ballew SH, Evans M, Arnlov J, et al. Hyperkalemia After Initiating Renin-Angiotensin System Blockade: The Stockholm Creatinine Measurements (SCREAM) Project. J Am Heart Assoc. 2017 Jul 19;6(7):e005428. doi: 10.1161/JAHA.116.005428. PMID: 28724651; PMCID: PMC5586281.

12. Upadhyay, A., Jaber, B.L. and Madias, N.E. (2006) Incidence and prevalence of hyponatremia. American Journal of Medicine 119(7 Suppl 1), S30-S35.

13. Spasovski, G., Vanholder, R., Allolio, B., et al. (2014) Clinical practice guideline on diagnosis and treatment of hyponatraemia. European Journal of Endocrinology 170(3), G1-G47.

14. Liamis, G., Milionis, H. and Elisaf, M. (2008) A review of drug-induced hyponatremia. *American Journal of Kidney Diseases* **52**(1), 144-153.

15. Yamamoto, Y., Takahashi, Y., Imai, K. et al. (2019) Prevalence and risk factors for hyponatremia in adult epilepsy patients: Large-scale cross-sectional cohort study. Seizure 73, 26-30.

16. Jacob, P., Dow, C., Lasker, S.S. et al. (2019) Hyponatraemia in primary care. BMJ 365, 1774.

17. Palmer, B.F., Carrero, J.J., Clegg, D.J., Colbert, G.B., Emmett, M., Fishbane, S., Hain, D.J., et al., 2021. Clinical Management of Hyperkalemia. Mayo Clinic Proceedings 96, 744–762.. doi:10.1016/j.mayocp.2020.06.014

18. Inker LA, Eneanya ND, Coresh J, Tighiouart H, Wang D, Sang Y, et al., Chronic Kidney Disease Epidemiology Collaboration. New Creatinine- and Cystatin C-Based Equations to Estimate GFR without Race. N Engl J Med. 2021 Nov 4;385(19):1737-1749. doi: 10.1056/NEJMoa2102953. Epub 2021 Sep 23. PMID: 34554658; PMCID: PMC8822996.

19. NATIONAL INSTITUTE FOR HEALTH AND CARE EXCELLENCE, Final appraisal document, Patiromer for treating hyperkalaemia, https://www.nice.org.uk/guidance/ta623/documents/final-appraisal-determination-document. Published Dec 2019. Assessed Oct 2023

20. European Resuscitation Council Guidelines 2021: Cardiac arrest in special circumstances. https://cprguidelines.eu/. Accessed Oct 2023.

##

## **Table S3.** Baseline characteristics – External validation data (CPRD Aurum)

| **Variable** | **Total**  **(N = 3,805,366)** | **Hyperkalaemia**  **(n = 277,982)** | **Competing - Hyperkalaemia**  **(n = 356,193)** | **Hyponatraemia**  **(n = 424,126)** | **Competing - Hyponatraemia**  **(n = 298,889)** |
| --- | --- | --- | --- | --- | --- |
| **Age, years – mean (SD)** | 58.6 (13.3) | 63.7 (12.7) | 74.1 (12.3) | 67 (12.9) | 74.2 (12.4) |
| **BMI** | 27.6 (5.4) | 28 (5.7) | 26.3 (5.4) | 27 (5.5) | 26.5 (5.5) |
| **Follow up, years – median (IQR)** | 7.0 (2.9-10.0) | 9.8 (5.9-10.0) | 3.8 (1.7-6.5) | 8.9 (5.0-10.0) | 3.5 (1.5-6.3) |
| **Systolic blood pressure, mmHg – mean (SD)** | 143.8 (12.3) | 146.5 (13.1) | 147.7 (13.2) | 148.1 (13.4) | 147.4 (13.2) |
| **Diastolic blood pressure, mmHg – mean (SD)** | 83.9 (9.8) | 83.5 (10.3) | 81.9 (10.3) | 83.3 (10.3) | 81.8 (10.3) |
| **Electronic Frailty index (eFI) – mean (SD)** | 0.06 (0.07) | 0.09 (0.07) | 0.11 (0.09) | 0.09 (0.08) | 0.11 (0.09) |
| **Sex** |  |  |  |  |  |
| Male | 1845877 (48.5%) | 143174 (51.5%) | 169963 (47.7%) | 187354 (44.2%) | 146015 (48.9%) |
| Female | 1959489 (51.5%) | 134808 (48.5%) | 186230 (52.3%) | 236772 (55.8%) | 152874 (51.1%) |
| **BMI** |  |  |  |  |  |
| Underweight | 42804 (1.1%) | 3229 (1.2%) | 10381 (2.9%) | 8106 (1.9%) | 8350 (2.8%) |
| Normal | 1030725 (27.1%) | 69214 (24.9%) | 97580 (27.4%) | 124254 (29.3%) | 79255 (26.5%) |
| Overweight | 1231169 (32.4%) | 89364 (32.1%) | 89411 (25.1%) | 125191 (29.5%) | 75070 (25.1%) |
| Obese | 757117 (19.9%) | 59864 (21.5%) | 45940 (12.9%) | 72917 (17.2%) | 39934 (13.4%) |
| Morbidly obese | 95008 (2.5%) | 8651 (3.1%) | 5113 (1.4%) | 9381 (2.2%) | 4742 (1.6%) |
| Missing | 648543 (17%) | 47660 (17.1%) | 107768 (30.3%) | 84277 (19.9%) | 91538 (30.6%) |
| **Deprivation Score** |  |  |  |  |  |
| IMD 1 | 790311 (20.8%) | 51218 (18.4%) | 70926 (19.9%) | 84922 (20%) | 58451 (19.6%) |
| IMD 2 | 732246 (19.2%) | 51878 (18.7%) | 72522 (20.4%) | 80694 (19%) | 60515 (20.2%) |
| IMD 3 | 684288 (18%) | 52062 (18.7%) | 70855 (19.9%) | 77634 (18.3%) | 59427 (19.9%) |
| IMD 4 | 630482 (16.6%) | 49969 (18%) | 69770 (19.6%) | 72228 (17%) | 58889 (19.7%) |
| IMD 5 | 597180 (15.7%) | 49947 (18%) | 71799 (20.2%) | 70675 (16.7%) | 61328 (20.5%) |
| Missing | 370859 (9.7%) | 22908 (8.2%) | 321 (0.1%) | 37973 (9%) | 279 (0.1%) |
| **Ethnicity** |  |  |  |  |  |
| White | 2041505 (53.6%) | 188486 (67.8%) | 233219 (65.5%) | 307872 (72.6%) | 190829 (63.8%) |
| Black | 115279 (3%) | 8099 (2.9%) | 3768 (1.1%) | 5953 (1.4%) | 3706 (1.2%) |
| South Asian | 94485 (2.5%) | 11506 (4.1%) | 3224 (0.9%) | 10380 (2.4%) | 2953 (1%) |
| Other | 832614 (21.9%) | 43145 (15.5%) | 19967 (5.6%) | 52199 (12.3%) | 17152 (5.7%) |
| Missing | 721483 (19%) | 26746 (9.6%) | 96015 (27%) | 47722 (11.3%) | 84249 (28.2%) |
| **Smoking status** |  |  |  |  |  |
| Non-smoker | 1475708 (38.8%) | 94017 (33.8%) | 119706 (33.6%) | 146980 (34.7%) | 100468 (33.6%) |
| Ex-smoker | 1236061 (32.5%) | 99154 (35.7%) | 109992 (30.9%) | 143988 (33.9%) | 92522 (31%) |
| Smoker | 838404 (22%) | 64658 (23.3%) | 76817 (21.6%) | 95868 (22.6%) | 63509 (21.2%) |
| Missing | 255193 (6.7%) | 20153 (7.2%) | 49678 (13.9%) | 37290 (8.8%) | 42390 (14.2%) |
| **Alcohol** |  |  |  |  |  |
| Non drinker | 864865 (22.7%) | 77010 (27.7%) | 95906 (26.9%) | 109911 (25.9%) | 81706 (27.3%) |
| Trivial drinker | 998948 (26.3%) | 70063 (25.2%) | 74759 (21%) | 105112 (24.8%) | 62454 (20.9%) |
| Light drinker | 696369 (18.3%) | 45603 (16.4%) | 46110 (12.9%) | 67984 (16%) | 37825 (12.7%) |
| Moderate drinker | 246468 (6.5%) | 17883 (6.4%) | 17614 (4.9%) | 28116 (6.6%) | 13783 (4.6%) |
| Heavy drinker | 74005 (1.9%) | 5266 (1.9%) | 7483 (2.1%) | 11350 (2.7%) | 5492 (1.8%) |
| Unknown amount | 237464 (6.2%) | 12265 (4.4%) | 13365 (3.8%) | 17465 (4.1%) | 11519 (3.9%) |
| Missing | 687247 (18.1%) | 49892 (17.9%) | 100956 (28.3%) | 84188 (19.8%) | 86110 (28.8%) |
| **Previous Hyperkalaemia within 2 years** | 30037 (0.8%) | 9751 (3.5%) | 3672 (1%) | 5916 (1.4%) | 4116 (1.4%) |
| **Previous Hyponatraemia within 2 years** | 107763 (2.8%) | 16251 (5.8%) | 25670 (7.2%) | 56970 (13.4%) | 13404 (4.5%) |
| **Frailty index groups** |  |  |  |  |  |
| Fit | 3256425 (85.6%) | 208512 (75%) | 227397 (63.8%) | 305550 (72%) | 190178 (63.6%) |
| Mildly frail | 447950 (11.8%) | 56302 (20.3%) | 95848 (26.9%) | 95926 (22.6%) | 80303 (26.9%) |
| Moderately frail | 82883 (2.2%) | 11127 (4%) | 26791 (7.5%) | 19034 (4.5%) | 23005 (7.7%) |
| Severely frail | 18108 (0.5%) | 2041 (0.7%) | 6157 (1.7%) | 3616 (0.9%) | 5403 (1.8%) |
| **eGFR (CKD stages)** |  |  |  |  |  |
| Stage 1 (G1) – normal | 691650 (18.2%) | 37879 (13.6%) | 25381 (7.1%) | 60390 (14.2%) | 19223 (6.4%) |
| Stage 2 (G2) – mild reduction, normal if young | 918677 (24.1%) | 78782 (28.3%) | 83189 (23.4%) | 121597 (28.7%) | 66417 (22.2%) |
| Stage 3a (G3a) – mild-moderate reduction | 179481 (4.7%) | 28019 (10.1%) | 41878 (11.8%) | 41336 (9.7%) | 35239 (11.8%) |
| Stage 3b (G3b) – moderate-severe reduction | 58885 (1.5%) | 13214 (4.8%) | 20281 (5.7%) | 14607 (3.4%) | 18771 (6.3%) |
| Stage 4 (G4) – severe reduction | 13622 (0.4%) | 4210 (1.5%) | 4993 (1.4%) | 3172 (0.7%) | 5291 (1.8%) |
| Stage 5 (G5) – kidney failure | 2888 (0.1%) | 970 (0.3%) | 909 (0.3%) | 662 (0.2%) | 1034 (0.3%) |
| No test | 1940163 (51%) | 114908 (41.3%) | 179562 (50.4%) | 182362 (43%) | 152914 (51.2%) |
| **Comorbidities** |  |  |  |  |  |
| Heart Failure | 71448 (1.9%) | 12554 (4.5%) | 26712 (7.5%) | 17913 (4.2%) | 23336 (7.8%) |
| Diabetes | 324175 (8.5%) | 60715 (21.8%) | 44250 (12.4%) | 79259 (18.7%) | 38390 (12.8%) |
| Coronary artery disease | 280526 (7.4%) | 42871 (15.4%) | 66171 (18.6%) | 60302 (14.2%) | 57093 (19.1%) |
| Peripheral vascular disease | 57723 (1.5%) | 9793 (3.5%) | 16954 (4.8%) | 14465 (3.4%) | 14453 (4.8%) |
| Cerebrovascular disease | 132928 (3.5%) | 15944 (5.7%) | 38721 (10.9%) | 28388 (6.7%) | 33224 (11.1%) |
| Chronic liver disease | 17124 (0.5%) | 1796 (0.6%) | 3051 (0.9%) | 3668 (0.9%) | 2186 (0.7%) |
| **Medications** |  |  |  |  |  |
| Loop diuretics | 221156 (5.8%) | 32876 (11.8) | 74895 (21%) | 49772 (11.7%) | 65448 (21.9%) |
| Potassium sparing diuretics | 84586 (2.2%) | 11800 (4.2%) | 26540 (7.5%) | 21895 (5.2%) | 21863 (7.3%) |
| ACE inhibitors | 478778 (12.6%) | 67069 (24.1%) | 67316 (18.9%) | 95828 (22.6%) | 56864 (19%) |
| Beta blockers | 461329 (12.1%) | 52819 (19%) | 61024 (17.1%) | 84280 (19.9%) | 50451 (16.9%) |
| Calcium channel blockers | 426151 (11.2%) | 47992 (17.3%) | 66955 (18.8%) | 77401 (18.2%) | 56403 (18.9%) |
| Thiazides and thiazide-like diuretics | 397980 (10.5%) | 35404 (12.7%) | 61188 (17.2%) | 92538 (21.8%) | 45974 (15.4%) |
| Alpha blockers | 68131 (1.8%) | 9162 (3.3%) | 11473 (3.2%) | 14269 (3.4%) | 9865 (3.3%) |
| Angiotensin II receptor antagonists | 136926 (3.6%) | 17224 (6.2%) | 14587 (4.1%) | 26676 (6.3%) | 11922 (4%) |
| Other antihypertensives | 44986 (1.2%) | 6211 (2.2%) | 9929 (2.8%) | 9717 (2.3%) | 8311 (2.8%) |

IMD – Indicies of multiple deprivation; BMI – Body mass index; eGFR – estimated glomerular filtration rate; CKD – Chronic kidney disease; ACE – Angiotensin converting enzyme; NSAIDS – Non-steroidal anti-inflammatory drugs

## **Figure S2:** Calibration curves (recalibration) for the apparent performance of the final STRATIFY models in CPRD GOLD. Groups represent tenths of the linear predictor, as created between deciles. Histograms show the distribution of predicted probabilities.

|  | **Hyperkalaemia model** | **Hyponatraemia model** |
| --- | --- | --- |
| **1 year** | **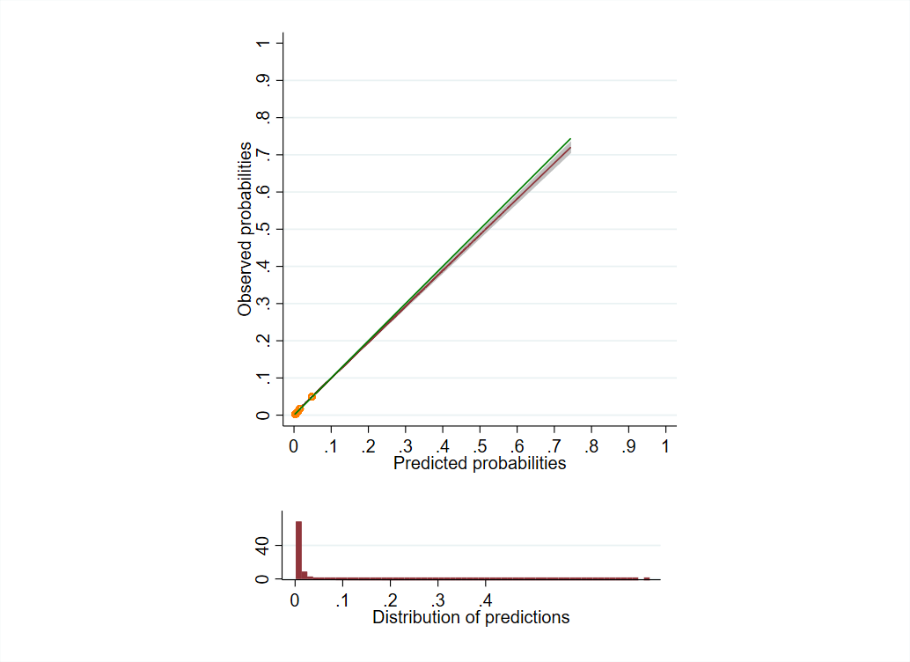** | **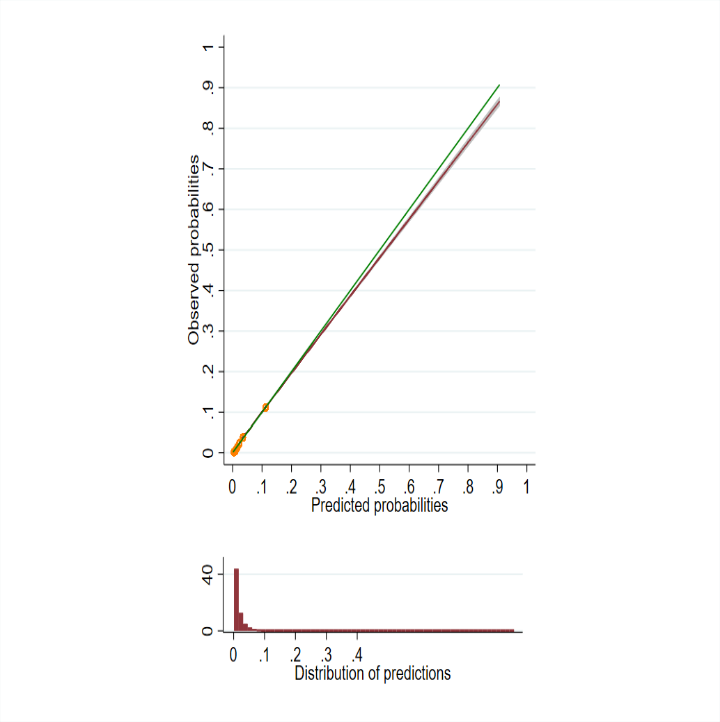** |
| **5 years** | 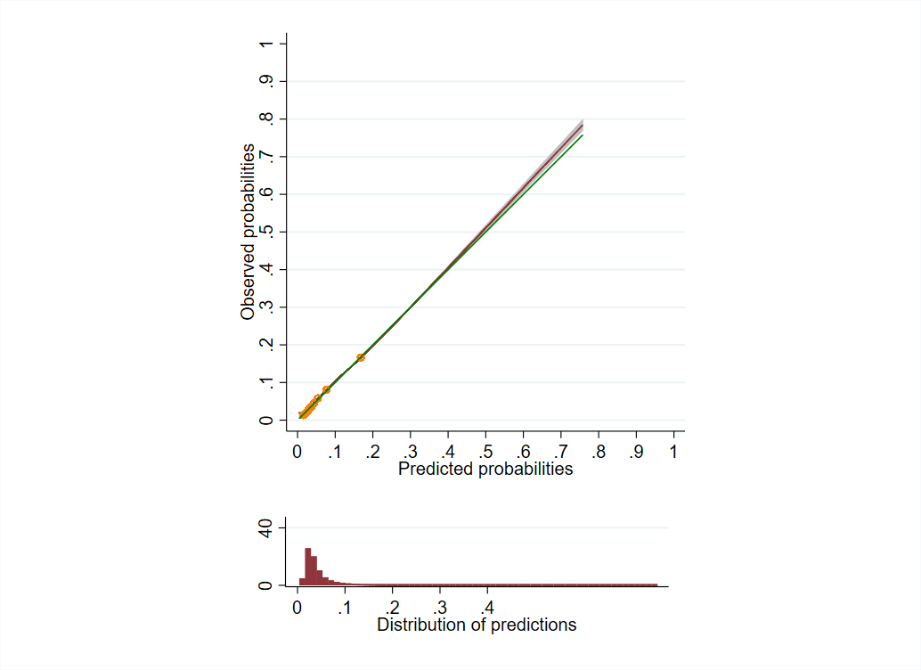 | 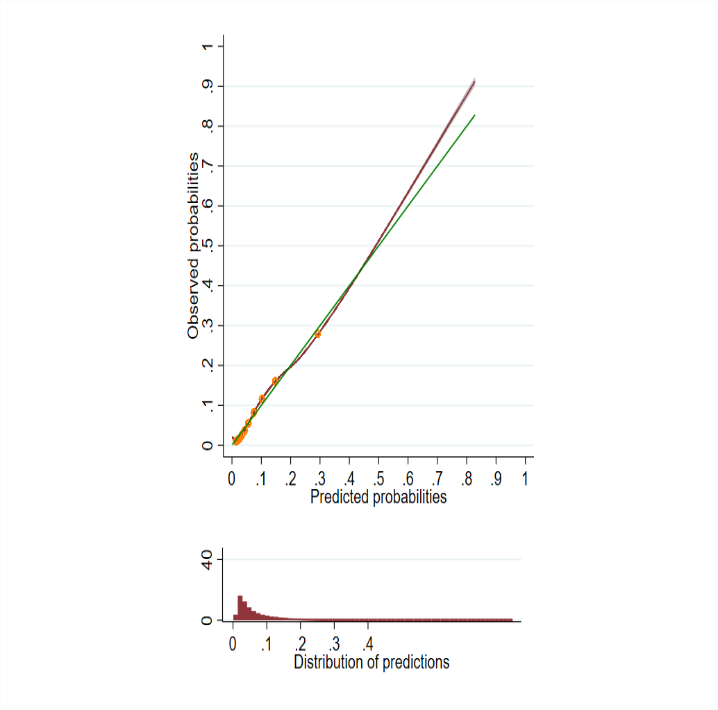 |
| **10 years** | 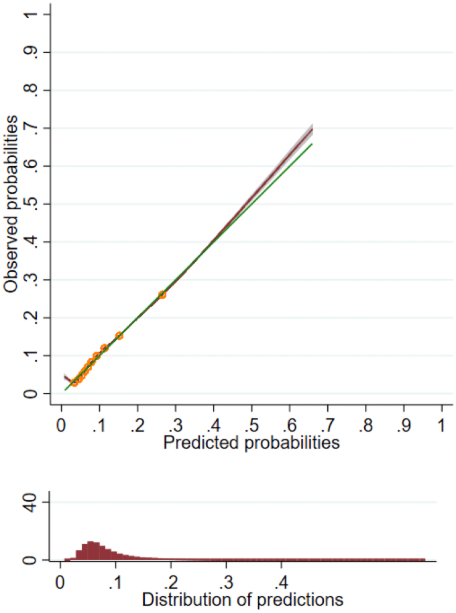 | **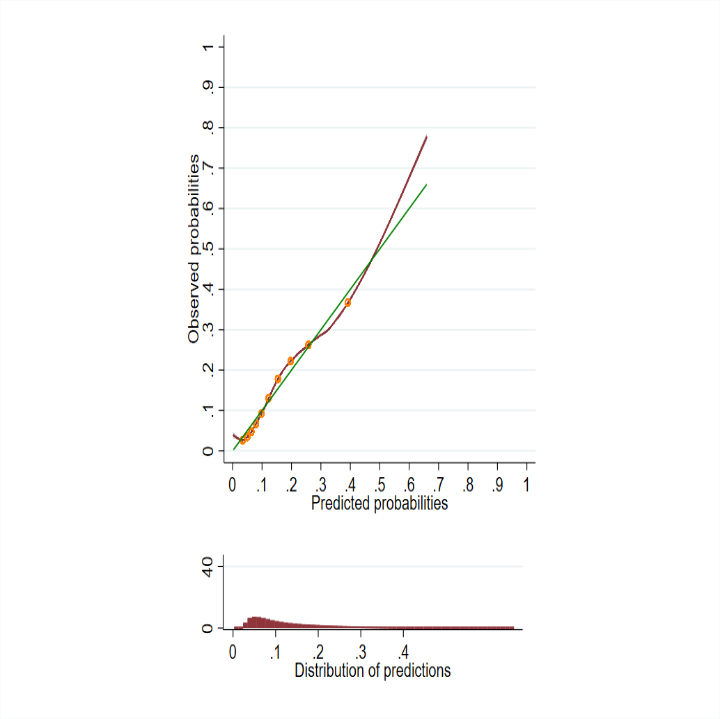** |

## **Figure S3:** Performance variability of the STRATIFY Hyperkalaemia model on external validation across GP practices, with panels (A) Observed/Expected, (B) $R_{D}^{2}$, (C) D-statistic, and (D) C-statistic

| A  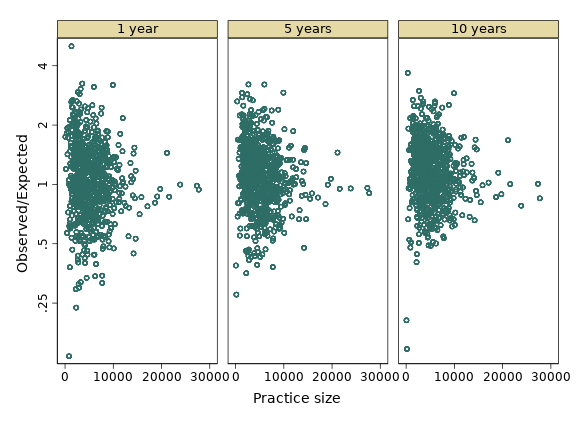 | B  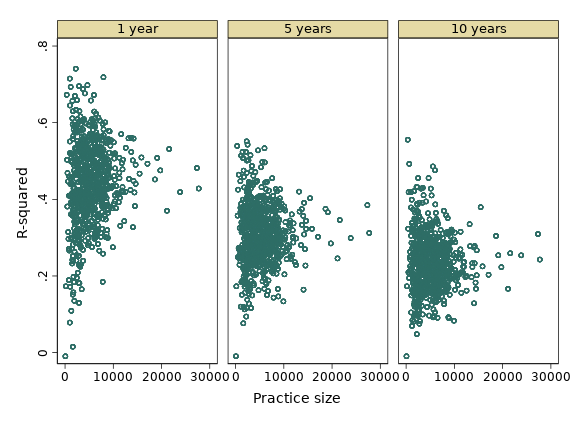 |
| --- | --- |
| C  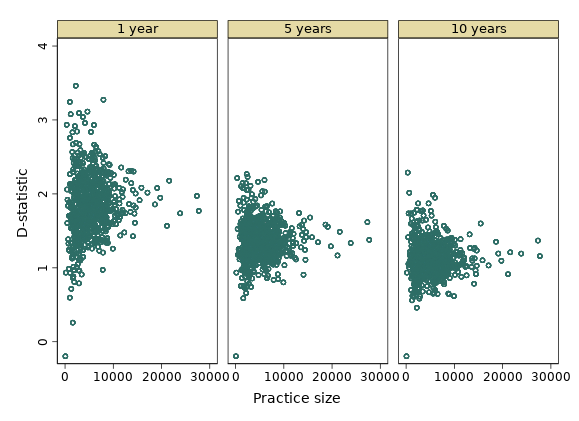 | D  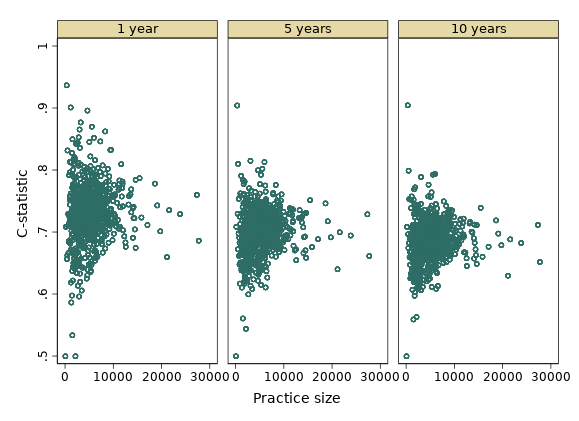 |

## **Figure S4:** Performance variability of the STRATIFY Hyponatraemia model on external validation across GP practices, with panels (A) Observed/Expected, (B) $R_{D}^{2}$, (C) D-statistic, and (D) C-statistic

| A  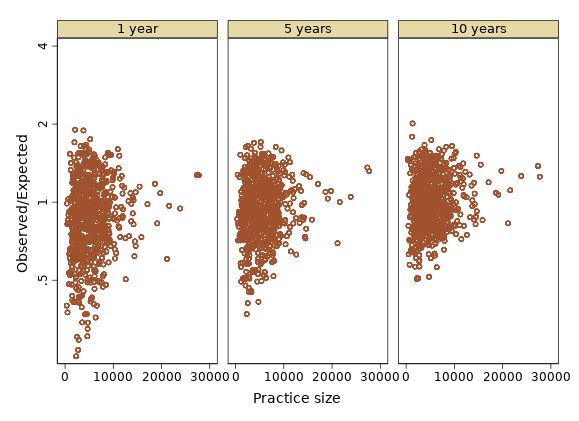 | B  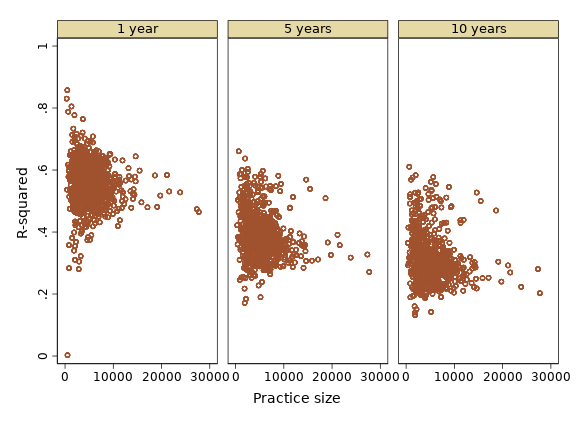 |
| --- | --- |
| C  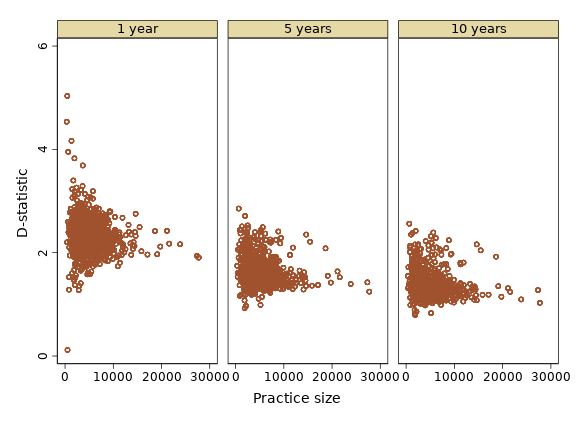 | D  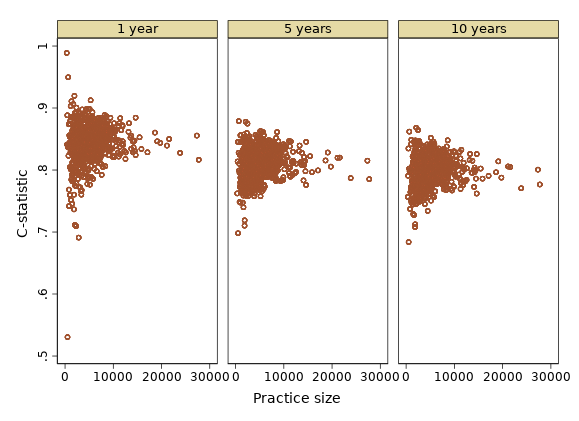 |

## **Figure S5.** Scatter plot showing the number of events (by outcome time point), against total practice size for practices in the external validation data.

| **Hyperkalaemia model** |
| --- |
| 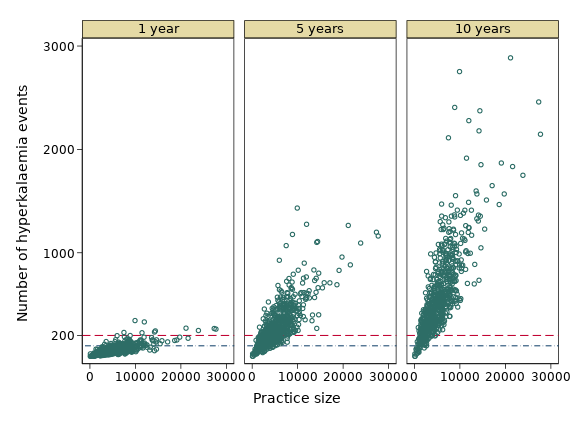 |
| **Hyponatraemia model** |
| 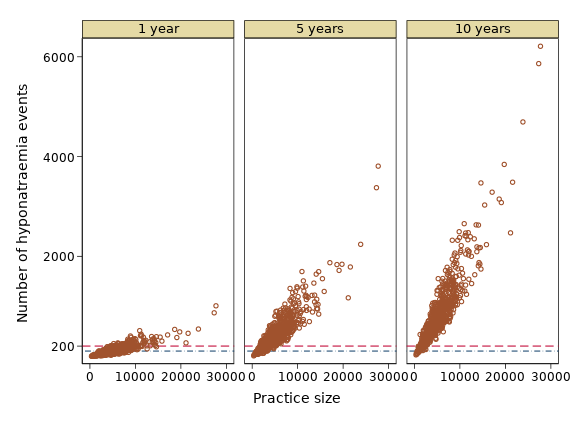 |

## **Figure S6.** Comparison of 10-year cardiovascular risk (QRisk2) and final STRATIFY-Hyperkalaemia and STRATIFY-Hyponatraemia risk in the CPRD GOLD dataset, using 10% risk threshold


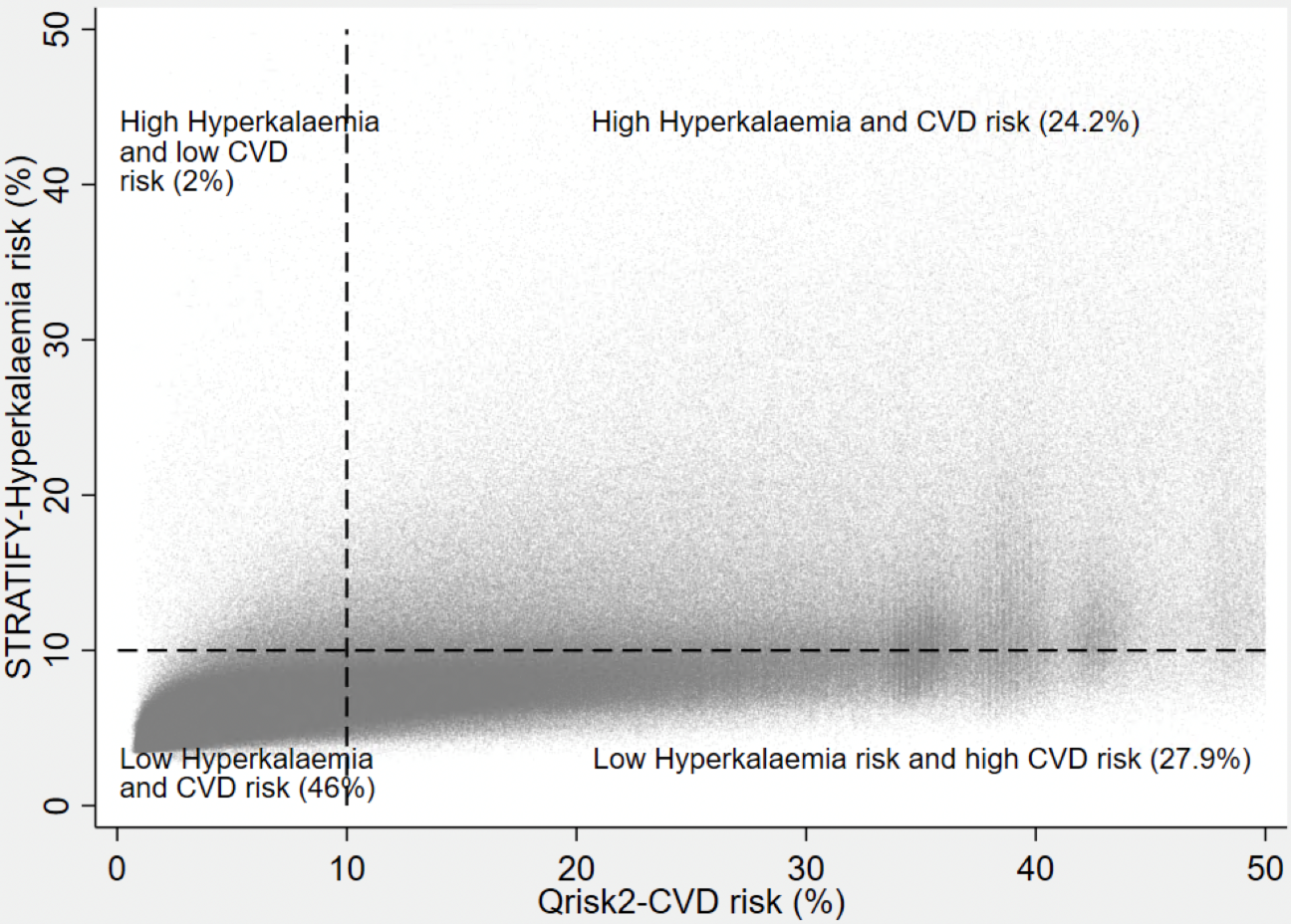


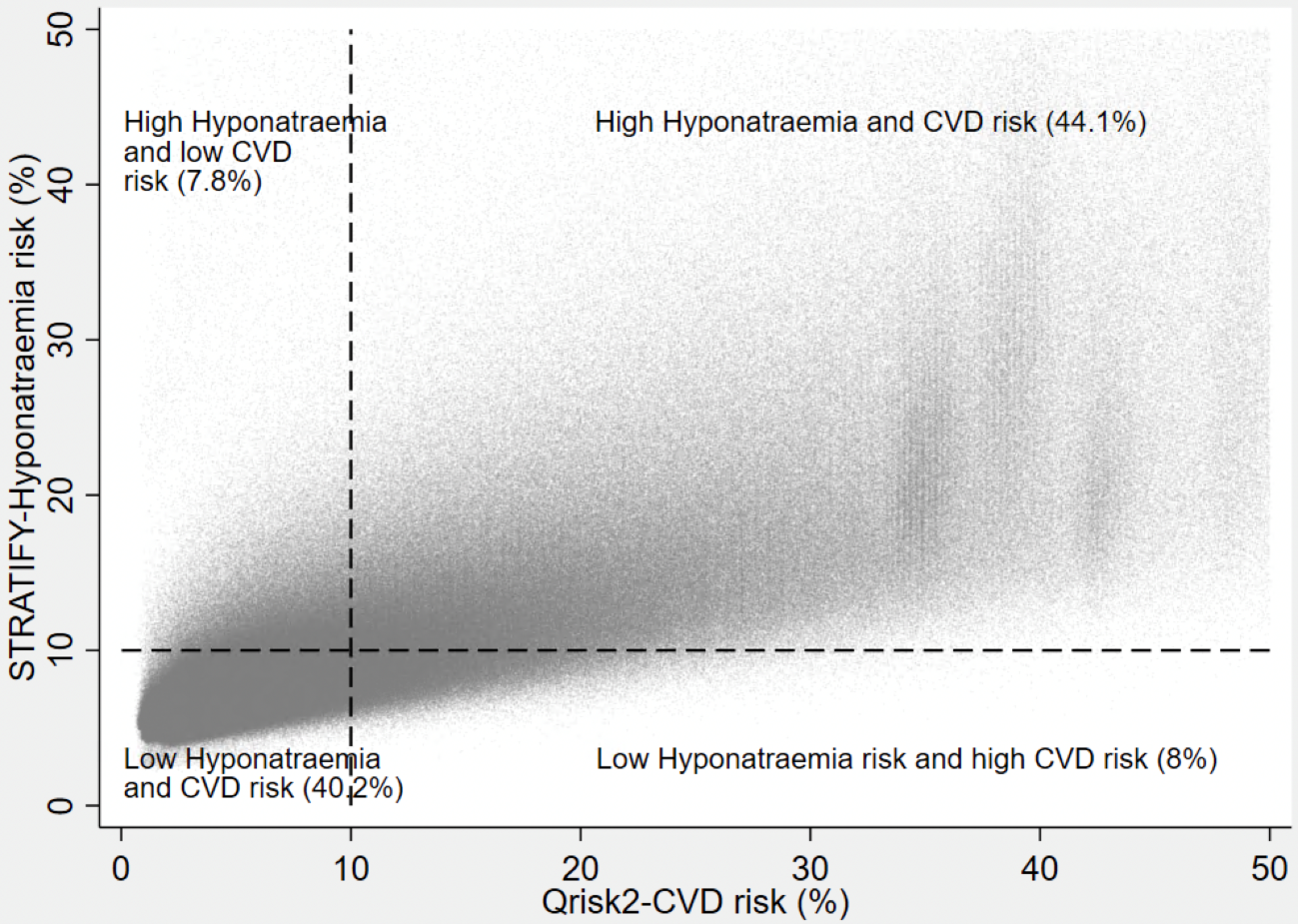


## **Figure S7.** Venn diagrams showing the overlap of high-risk patients (≥10%) as classified using the final STRATIFY-Hyperkalaemia and STRATIFY-Hyponatraemia, as compared with Qrisk2 CVD risks at 10 years in the CPRD Gold cohort (derivation)


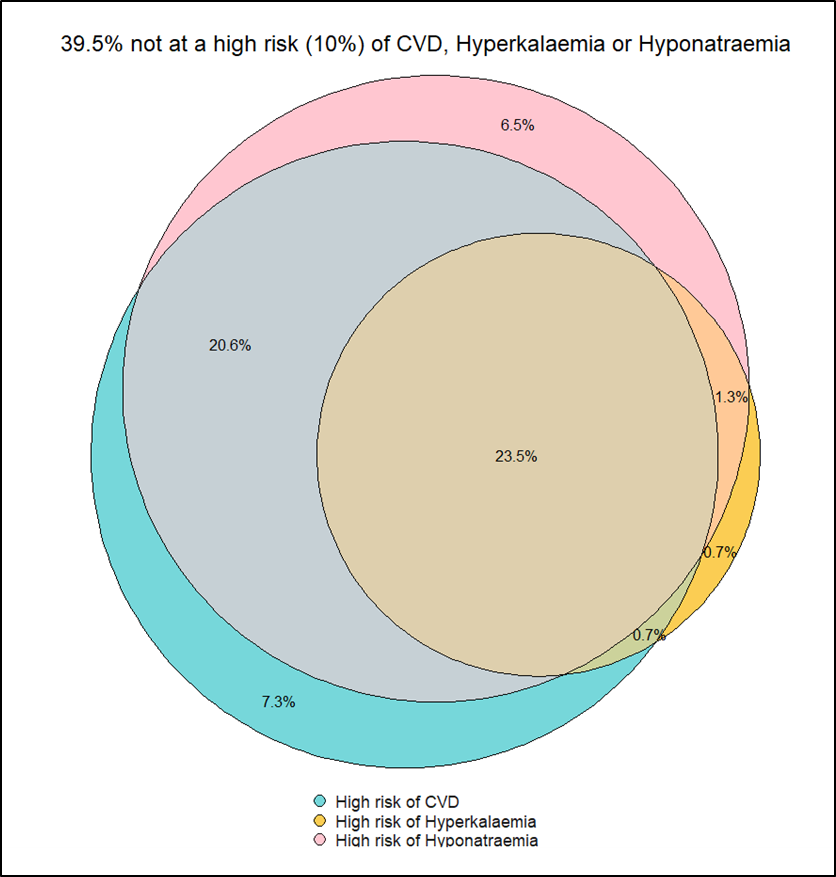


## **Figure S8.** Distribution of linear predictor, grouped by outcome type at 10 years

| **Hyperkalaemia model** | | | |
| --- | --- | --- | --- |
| Hyperkalaemia event | Competing event (death) | Censored at or before 10 years | All |
| 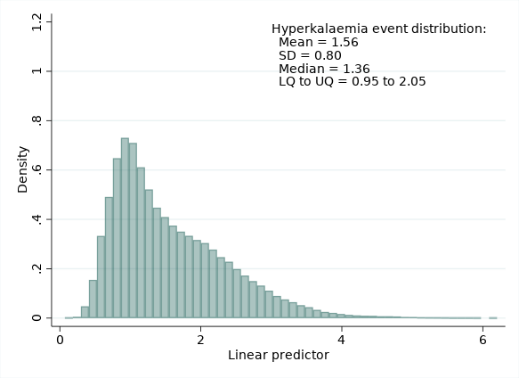 | 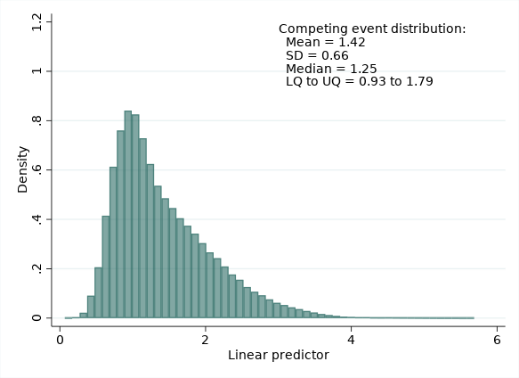 | 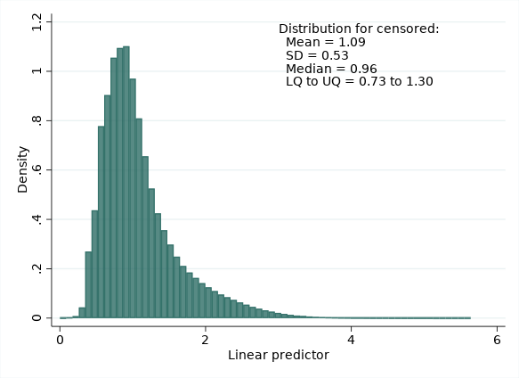 | 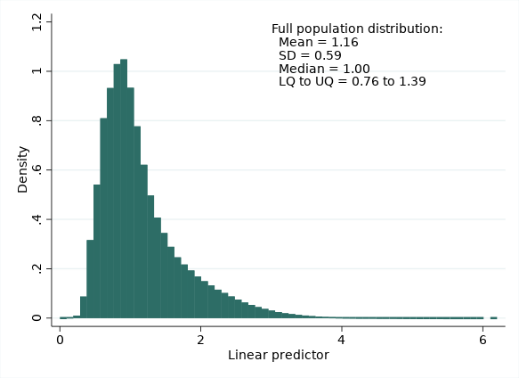 |
| **Hyponatraemia model** | | | |
| Hyponatraemia event | Competing event (death) | Censored at or before 10 years | All |
| 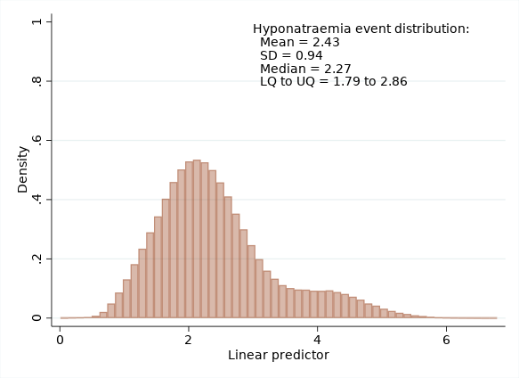 | 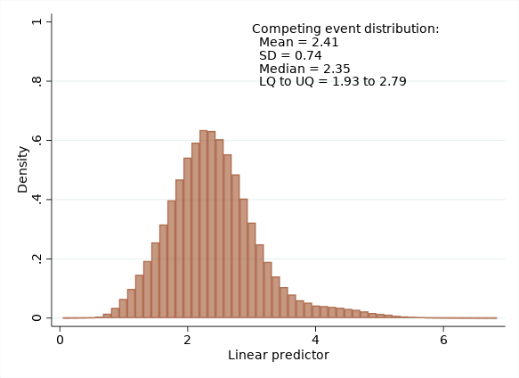 | 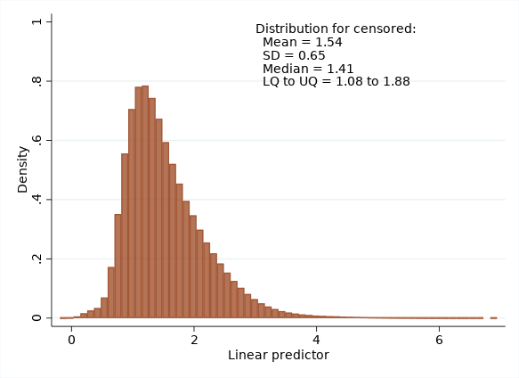 | 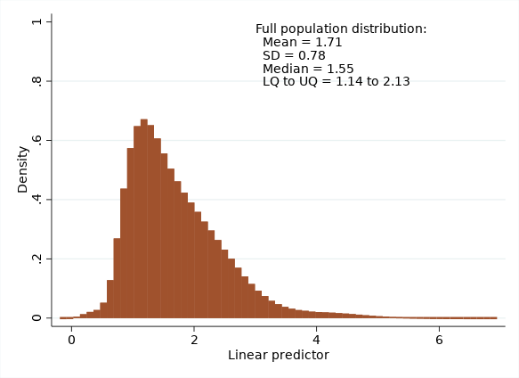 |

# Appendix 4. Supplementary Equations

| **Hyperkalaemia:**  Final model equation for 1, 5, and 10 years:  $t year {risk}_{i}=1-(((1-{CIF}^{*}))^exp({LP}_{i}))$  Where,  ${LP}_{i}=\left( \left( \left( \frac{age}{10} \right)^{2} \right)- 37.03 \right)*0.0047+(SBP*0.0045)$  $+ (FI*10)*0.1213$  $+\left\{ \begin{aligned} -0.1516 if female \\ 0 if male \end{aligned} \right. +\left\{ \begin{aligned} 0.3563 if CKD stage 2 \\ 0.7184 if CKD stage 3a \\ 0.9214 if CKD stage 3b \\ 1.1276 if CKD stage 4 \\ 0.9789 if CKD stage 5 \end{aligned} \right.$  $+\left\{ \begin{aligned} -0.0251 if underweight \\ 0.0498 if overweight \\ 0.1214 if obese \\ 0.3032 if morbidly obese \end{aligned} \right.+\left\{ \begin{aligned} -0.0667 if black ethnicity \\ 0.5609 if south asian ethnicity \\ 0.1913 if other ethnicity \end{aligned} \right.$  $+\left\{ \begin{aligned} 0.1752 if ex smoker \\ 0.3306 if current smoker \end{aligned} \right.$  $+ \left( 1.2915 if Previous hyperkalaemia \right)+\left( -0.0922 if Heart failure \right)+\left( 0.8640 if Diabetes \right)+\left( -0.1150 if Cerebrovascular disease \right)+\left( 0.1165 if Coronary artery disease \right)+\left( 0.1007 if Peripheral vascular disease \right) +\left( 0.4439 if Ace inhibitors \right)+\left( 0.3123 if Angiotensin \right)+\left( -0.0020 if Alpha blockers \right)+\left( 0.1891 if Beta blockers \right)+\left( 0.0406 if Calcium channel blockers \right)+\left( 0.1860 if Other antihyprtensive \right)+\left( -0.2689 if Thiazides and thiazide-like diuretics \right)+\left( -0.1206 if Loop diuretics \right)+\left( 0.0703 if Potassium sparing diuretics \right)$   - $\exp$ is the exponential function, $\ln$ is the natural logarithm - ${CIF}^{*}$ is the baseline CIF value for 1, 5 and 10 years - CKD stages refer to chronic kidney disease stages measured by eGFR - age is measured in years and SBP is measured in mm Hg |
| --- |

#

| **Hyponatraemia:**  Final model equation for 1, 5 and 10 years:  $t year {risk}_{i}=1-(((1-{CIF}^{*}))^exp({LP}_{i}))$  Where,  ${LP}_{i}=\left( \left( \left( \frac{age}{10} \right)^{2} \right)- 37.03 \right)*0.0219+(SBP*0.0090)$  $+ (FI*10)*0.1625$ $+\left\{ \begin{aligned} 0.0702 if ex smoker \\ 0.2985 if current smoker \end{aligned} \right.$  $+\left\{ \begin{aligned} 0.0755 if female \\ 0 if male \end{aligned} \right. +\left\{ \begin{aligned} -0.0114 if CKD stage 2 \\ -0.0648 if CKD stage 3a \\ -0.2245 if CKD stage 3b \\ -0.3784 if CKD stage 4 \\ -0.2123if CKD stage 5 \end{aligned} \right.$  $+\left\{ \begin{aligned} 0.0606 if underweight \\ -0.1463 if overweight \\ -0.2158 if obese \\ -0.1615 if morbidly obese \end{aligned} \right.+\left\{ \begin{aligned} -0.5870 if black ethnicity \\ 0.1986 if south asian ethnicity \\ -0.1486 if other ethnicity \end{aligned} \right.$  $+\left\{ \begin{aligned} 0.0251 if IMD2 \\ 0.0321 if IMD3 \\ 0.0749 if IMD4 \\ 0.1099 if IMD5 \end{aligned} \right.+\left\{ \begin{aligned} -0.0014 if trivial drinker \\ 0.0409 if light drinker \\ 0.1169if moderate drinker \\ 0.4941 if heavy drinker \\ 0.0228 if drinker \left( unkown quantity \right) \end{aligned} \right.$  $+ (1.5985 if Previous hyponatraemia)+\left( -0.1057 if Heart failure \right)+\left( 0.7341 if Diabetes \right)+\left( -0.1678 if Cerebrovascular disease \right)+\left( 0.5952 if Chronic liver disease \right)+\left( 0.3033 if Ace inhibitors \right)+\left( 0.2630 if Angiotensin \right)+\left( 0.0763 if Alpha blockers \right)+\left( 0.1909 if Beta blockers \right)+\left( 0.0552 if Calcium channel blockers \right)+\left( 0.3854 if Thiazides and thiazide-like diuretics \right)+\left( 0.1309 if Other antihyprtensive \right)+\left( -0.1129 if Loop diuretics \right)+\left( 0.2344 if Potassium sparing diuretics \right)+\left( 0.0639 if Antidepressants \right)+\left( 0.6250 ifAnticonvulsants \right) +\left( -0.0502 if Antipsychotics \right) +\left( 0.1112 if NSAIDS \right) +\left( 0.1571 if Proton pump inhibitors \right)$   - $\exp$ is the exponential function, $\ln$ is the natural logarithm - ${CIF}^{*}$ is the baseline CIF value for 1, 5 and 10 years - CKD stages refer to chronic kidney disease stages measured by eGFR - age is measured in years and SBP is measured in mm Hg - IMD2-IMD5 refer to indices of multiple deprivation - NSAIDS refer to Non-steroidal anti-inflammatory drugs |
| --- |

# Appendix 5. Protocol

**INDEPENDENT SCIENTIFIC ADVISORY COMMITTEE (ISAC) PROTOCOL APPLICATION FORM**

**PART 1: APPLICATION FORM**

***IMPORTANT***

**Both parts of this application must be completed in accordance with the guidance note ‘Completion of the ISAC Protocol Application Form’, which can be found on the CPRD website** [**cprd.com/research-applications**](https://cprd.com/research-applications)

| **FOR ISAC USE ONLY** | |
| --- | --- |
| **Protocol No. -** | **Submission date -** |

| **GENERAL INFORMATION ABOUT THE PROPOSED RESEARCH STUDY** |
| --- |
| 1. **Study Title (Max. 255 characters)**   Understanding the harms of antihypertensives, statins and antiplatelets for prevention of cardiovascular disease: A prognostic modelling and casual inference study |
| 1. **Research Area** (place ‘**X**’ in all boxes that apply) |
| \| Drug Safety \| x \| Economics \|  \| \| --- \| --- \| --- \| --- \| \| Drug Utilisation \|  \| Pharmacoeconomics \|  \| \| Drug Effectiveness \|  \| Pharmacoepidemiology \|  \| \| Disease Epidemiology \|  \| Methodological \|  \| \| Health Services Delivery \| x \|  \|  \| |
| 1. **Chief Investigator**  \| Title: \| Dr \| \| --- \| --- \| \| Full name: \| James Sheppard \| \| Job title: \| University Research Lecturer \| \| Affiliation/organisation: \| University of Oxford \| \| Email address: \| [James.sheppard@phc.ox.ac.uk](mailto:James.sheppard@phc.ox.ac.uk) \| \| CV Number (if applicable): \| 034_16 \| |
| 1. **Corresponding Applicant**  \| Title: \| Dr \|  \| \| --- \| --- \| --- \| \| Full name: \| James Sheppard \|  \| \| Job title: \| University Research Lecturer \|  \| \| Affiliation/organisation: \| University of Oxford \|  \| \| Email address: \| [James.sheppard@phc.ox.ac.uk](mailto:James.sheppard@phc.ox.ac.uk) \|  \| \| CV Number (if applicable): \| 034_16 \|  \| |
| 1. **List of all investigators/collaborators**  \| Title: \| Prof \| \| --- \| --- \| \| Full name: \| Richard Stevens \| \| Job title: \| Associate Professor \| \| Affiliation/organisation: \| University of Oxford \| \| Email address: \| [Richard.stevens@phc.ox.ac.uk](mailto:Richard.stevens@phc.ox.ac.uk) \| \| CV Number (if applicable): \| 037_15CESL \| \| Will this person be analysing the data? (Y/N) \| N \|  \| Title: \| Prof \| \| --- \| --- \| \| Full name: \| Richard McManus \| \| Job title: \| Professor \| \| Affiliation/organisation: \| University of Oxford \| \| Email address: \| [Richard.mcmanus@phc.ox.ac.uk](mailto:Richard.mcmanus@phc.ox.ac.uk) \| \| CV Number (if applicable): \| 036_15CP \| \| Will this person be analysing the data? (Y/N) \| N \|  \| Title: \| Prof \| \| --- \| --- \| \| Full name: \| Richard Hobbs \| \| Job title: \| Professor \| \| Affiliation/organisation: \| University of Oxford \| \| Email address: \| [Richard.hobbs@phc.ox.ac.uk](mailto:Richard.hobbs@phc.ox.ac.uk) \| \| CV Number (if applicable): \| 035_16P \| \| Will this person be analysing the data? (Y/N) \| N \|  \| Title: \| Dr \| \| --- \| --- \| \| Full name: \| Sarah Lay-Flurrie \| \| Job title: \| Senior Statistician \| \| Affiliation/organisation: \| University of Oxford \| \| Email address: \| [Sarah.lay-flurrie@phc.ox.ac.uk](mailto:Sarah.lay-flurrie@phc.ox.ac.uk) \| \| CV Number (if applicable): \| 042_15ESL \| \| Will this person be analysing the data? (Y/N) \| Y \|  \| Title: \| Mr \| \| --- \| --- \| \| Full name: \| Constantinos Koshiaris \| \| Job title: \| Statistician \| \| Affiliation/organisation: \| University of Oxford \| \| Email address: \| [constantinos.koshiaris@phc.ox.ac.uk](mailto:constantinos.koshiaris@phc.ox.ac.uk) \| \| CV Number (if applicable): \| 204_17 \| \| Will this person be analysing the data? (Y/N) \| Y \|  \| Title: \| Prof \| \| --- \| --- \| \| Full name: \| Richard Riley \| \| Job title: \| Professor \| \| Affiliation/organisation: \| Keele University \| \| Email address: \| [r.riley@keele.ac.uk](mailto:r.riley@keele.ac.uk) \| \| CV Number (if applicable): \| n/a \| \| Will this person be analysing the data? (Y/N) \| Y \|  \| Title \| Dr \| \| --- \| --- \| \| Full name: \| Kym Snell \| \| Job title: \| Research Associate \| \| Affiliation/organisation: \| Keele University \| \| Email address: \| [k.snell@keele.ac.uk](mailto:k.snell@keele.ac.uk) \| \| CV Number (if applicable): \| n/a \| \| Will this person be analysing the data? (Y/N) \| Y \|  \| Title: \| Dr \| \| --- \| --- \| \| Full name: \| Rupert Payne \| \| Job title: \| Consultant Senior Lecturer \| \| Affiliation/organisation: \| University of Bristol \| \| Email address: \| [r.payne@bristol.ac.uk](mailto:r.payne@bristol.ac.uk) \| \| CV Number (if applicable): \| 433_17 \| \| Will this person be analysing the data? (Y/N) \| N \|  \| Title: \| Ms \| \| --- \| --- \| \| Full name: \| Lucinda Archer \| \| Job title: \| Research Associate in Biostatistics \| \| Affiliation/organisation: \| Keele University \| \| Email address: \| [l.archer@keele.ac.uk](mailto:l.archer@keele.ac.uk) \| \| CV Number (if applicable): \| n/a \| \| Will this person be analysing the data? (Y/N) \| Y \|  \| Title: \| Mr \| \| --- \| --- \| \| Full name: \| Subhashisa Swain \| \| Job title: \| Quantitative researcher \| \| Affiliation/organisation: \| University of Oxford \| \| Email address: \| subhashisa.swain@phc.ox.ac.uk \| \| CV Number (if applicable): \| 067_19 \| \| Will this person be analysing the data? (Y/N) \| Y \|  \| Title: \| Dr \| \| --- \| --- \| \| Full name: \| Tingting Zhu \| \| Job title: \| Royal Academy of Engineering Research Fellow \| \| Affiliation/organisation: \| University of Oxford \| \| Email address: \| tingting.zhu@eng.ox.ac.uk \| \| CV Number (if applicable): \| n/a \| \| Will this person be analysing the data? (Y/N) \| N \|  \| Title: \| Ms \| \| --- \| --- \| \| Full name: \| Ghadeer Ghosheh \| \| Job title: \| DPhil Student \| \| Affiliation/organisation: \| University of Oxford \| \| Email address: \| ghadeer.ghosheh@eng.ox.ac.uk \| \| CV Number (if applicable): \| n/a \| \| Will this person be analysing the data? (Y/N) \| Y \|  \| Title: \| Dr \| \| --- \| --- \| \| Full name: \| Ariel Wang \| \| Job title: \| Statistician \| \| Affiliation/organisation: \| University of Oxford \| \| Email address: \| Ariel.wang@phc.ox.ac.uk \| \| CV Number (if applicable): \| n/a \| \| Will this person be analysing the data? (Y/N) \| Y \| |
| 1. **Experience/expertise available**   List below the member(s) of the research team who have experience with CPRD data.   \| **Name:** \| **Protocol Number/s:** \| \| --- \| --- \| \| James Sheppard \| 18_059; 17_239; 16_008R \| \| Richard Stevens \| 17_239; 16_008R \| \| Richard McManus \| 17_239; 16_008R \| \| Richard Hobbs \| 17_239; 16_008R \| \| Sarah Lay-Flurrie \| 17_239; 16_008R \| \| Constantinos Koshiaris \| 17_088; 14_105 \| \| Rupert Payne \| 14_224, 15_106, 15_110, 15_239, 16_029, 16_057, 16_111, 16_117, 16_170, 16_180, 16_242, 17_051, 17_140, 17_184, 17_225, 18_160, 18_197 \|   List below the member(s) of the research team who have statistical expertise.   \| **Name(s):** \|  \| \| --- \| --- \| \| James Sheppard \| \| \| Richard Stevens \| \| \| Sarah Lay-Flurrie \| \| \| Constantinos Koshiaris \| \| \| Richard Riley \| \| \| Kym Snell \| \| \| Lucinda Archer \| \| \| Tingting Zhu \| \| \| Ghadeer Ghosheh \| \| \| Ariel Wang \| \|   List below the member(s) of the research team who have experience of handling large datasets (greater than 1 million records).   \| **Name(s):** \|  \| \| --- \| --- \| \| Richard Stevens \| \| \| Sarah Lay-Flurrie \| \| \| Constantinos Koshiaris \| \| \| Rupert Payne \| \| \| Tingting Zhu \| \| \| Ghadeer Ghosheh \| \|   List below the member(s) of the research team, or supporting the research team, who have experience of practicing in UK primary care.   \| **Name(s):** \|  \| \| --- \| --- \| \| Richard McManus \| \| \| Richard Hobbs \| \| \| Rupert Payne \| \| |
| **ACCESS TO THE DATA** |
| 1. **Sponsor of the study**  \| Institution/Organisation: \| University of Oxford \| \| --- \| --- \| \| Address: \| Nuffield Department of Primary Care Health Sciences  University of Oxford Radcliffe Primary Care  Radcliffe Observatory Quarter  Woodstock Road  Oxford  OX2 6GG \| |
| 1. **Funding source for the study**  \| Same as Sponsor? \| Yes \|  \| No \| x \|  \| \| --- \| --- \| --- \| --- \| --- \| --- \| \| Institution/Organisation: \| Wellcome Trust \| \| \| \| \| \| Address: \| Gibbs Building  215 Euston Road  London NW1 2BE  UK \| \| \| \| \|      \| Same as Sponsor? \| Yes \|  \| No \| x \|  \| \| --- \| --- \| --- \| --- \| --- \| --- \| \| Institution/Organisation: \| NIHR School for Primary Care Research \| \| \| \| \| \| Address: \| Nuffield Department of Primary Care Health Sciences  University of Oxford Radcliffe Primary Care  Radcliffe Observatory Quarter  Woodstock Road  Oxford  OX2 6GG \| \| \| \| \| |
| 1. **Institution conducting the research**  \| Same as Sponsor? \| Yes \| x \| No \|  \|  \| \| --- \| --- \| --- \| --- \| --- \| --- \| \| Institution/Organisation: \| University of Oxford \| \| \| \| \| \| Address: \| Nuffield Department of Primary Care Health Sciences  University of Oxford Radcliffe Primary Care  Radcliffe Observatory Quarter  Woodstock Road  Oxford  OX2 6GG \| \| \| \| \| |
| 1. **Data Access Arrangements**   Indicate with an ‘**X**’ the method that will be used to access the data for this study:   \| Study-specific Dataset Agreement \|  \| \| --- \| --- \|  \| Institutional Multi-study Licence \| x \|  \| \| --- \| --- \| --- \| \| Institution Name \| University of Oxford \| \| \| Institution Address \| Nuffield Department of Primary Care Health Sciences  University of Oxford Radcliffe Primary Care  Radcliffe Observatory Quarter  Woodstock Road  Oxford  OX2 6GG \| \|   Will the dataset be extracted by CPRD?   \| Yes \| x \| No \|  \| \| --- \| --- \| --- \| --- \|   We will extract data from the CPRD Gold  We request that from CPRD Aurum are extracted by CPRD  If yes, provide the reference number:  This has been discussed with CPRD via email (ref: CPRD00033433) |
| 1. **Data Processor(s):**  \| Processing \| x \|  \| \| --- \| --- \| --- \| \| Accessing \| x \| \| Storing \| x \| \| Processing area (UK/EEA/Worldwide) \| \| UK \| \| Organisation name \| \| University of Oxford \| \| Organisation address \| \| Nuffield Department of Primary Care Health Sciences  University of Oxford Radcliffe Primary Care  Radcliffe Observatory Quarter  Woodstock Road  Oxford  OX2 6GG \|  \| Processing \| x \|  \| \| --- \| --- \| --- \| \| Accessing \| x \| \| Storing \| x \| \| Processing area (UK/EEA/Worldwide) \| \| UK \| \| Organisation name \| \| Keele University \| \| Organisation address \| \| Arthritis Research UK Primary Care Centre  Research Institute for Primary Care & Health Sciences  Primary Care Sciences  Keele University  Staffordshire  ST5 5BG \|  \| Processing \| x \|  \| \| --- \| --- \| --- \| \| Accessing \| x \| \| Storing \| x \| \| Processing area (UK/EEA/Worldwide) \| \| UK \| \| Organisation name \| \| University of Birmingham \| \| Organisation address \| \| Institute for Applied Digital Health  University of Birmingham  Edgbaston  Birmingham  B15 2TT \|   [Add more processors as necessary by copy and pasting a new table for each processor] |
| **INFORMATION ON DATA** |
| 1. **Primary care data** (place ‘**X**’ in all boxes that apply)  \| CPRD GOLD \| x \| CPRD Aurum \| x \| \| --- \| --- \| --- \| --- \|   **X** |
| 1. **Please select any linked data or data products being requested**   **Patient Level Data** (place ‘**X**’ in all boxes that apply) |
| \| ONS Death Registration Data \| x \| CPRD Mother Baby Link \|  \| \| --- \| --- \| --- \| --- \| \| HES Admitted Patient Care \| x \| Pregnancy Register \|  \| \| HES Outpatient \|  \| NCRAS (National Cancer Registration and Analysis Service) Cancer Registration Data \|  \| \| HES Accident and Emergency \|  \| NCRAS Cancer Patient Experience Survey (CPES) data \|  \| \| HES Diagnostic Imaging Dataset \|  \| NCRAS Systemic Anti-Cancer Treatment (SACT) data \|  \| \| HES PROMS (Patient Reported Outcomes Measure) \|  \| NCRAS National Radiotherapy Dataset (RTDS) data \|  \| \|  \|  \| Mental Health Services Data Set (MHDS) \|  \| |
| **Area Level Data** (place ‘**X**’ in all boxes that apply)   \| **Practice level (UK)** \|  \| **Patient level (England only)** \|  \| \| --- \| --- \| --- \| --- \| \| Practice Level Index of Multiple Deprivation (Standard) \|  \| Patient Level Index of Multiple Deprivation \| x \| \| Practice Level Index of Multiple Deprivation (Non-standard) \|  \| Patient Level Townsend Score \|  \| \| Practice Level Index of Multiple Deprivation Domains (Non-standard) \|  \|  \|  \| \| Practice Level Carstairs Index for 2011 Census (Excluding Northern Ireland) (Standard) \|  \|  \|  \| \| 2011 Rural-Urban Classification at LSOA level (Non-standard) \|  \|  \|  \|   Reference number (where applicable): |
| 1. **Are you requesting linkage to a dataset not listed above?**  \| Yes \|  \| No \| x \| \| --- \| --- \| --- \| --- \|   If yes, provide the reference number: |
| 1. **Does any person named in this application already have access to any of these data in a patient identifiable form, or associated with an identifiable patient index?**  \| Yes \|  \| No \| x \| \| --- \| --- \| --- \| --- \|   If yes, provide further details: |
| **VALIDATION/VERIFICATION** |
| 1. **Does this protocol describe an observational study using purely CPRD data?**  \| Yes \| x \| No \|  \| \| --- \| --- \| --- \| --- \| |
| 1. **Does this protocol involve requesting any additional information from GPs, or contact with patients?**  \| Yes \|  \| No \| x \| \| --- \| --- \| --- \| --- \|   If yes, provide the reference number: |

**PART 2: PROTOCOL INFORMATION**

| **Applicants must complete all sections listed below**  **Sections which do not apply should be completed as ‘*Not Applicable’* and justification provided** |
| --- |
| 1. **Study Title (Max. 255 characters)**   Understanding the harms of antihypertensives, statins and antiplatelets for prevention of cardiovascular disease: A prognostic modelling and casual inference study |
| 1. **Lay Summary (Max. 250 words)**   People are living for longer, with more long-term physical and mental conditions which worsen their health. One example is high blood pressure, where people can take 3-4 drugs to prevent stroke. However, dozens of people have to be treated for at least a year to prevent a stroke in one person. This is because these drugs only reduce the possibility of stroke, they do not remove it altogether. Some of these patients may be prone to side effects such as falls and kidney problems which may be more common than any benefits.  This proposal aims to use information from the medical records from hundreds of thousands of patients to establish the link between drugs used to prevent heart attack and stroke and side effects. We will focus on drugs that lower blood pressure (known as antihypertensives), lower cholesterol (known as statins) or prevent blood clots (known as antiplatelets). This information will be used to develop a calculator which predicts a person’s risk of experiencing side effects. This calculator will form part of a support tool which will help patients and doctors make better informed decisions about starting or continuing drugs. |
| 1. **Technical Summary (Max. 300 words)**   *Background*  The population is ageing and consequently, the number of people living with age-related chronic conditions is increasing. Polypharmacy (five or more prescribed medications) is common in older people and is associated with an increased risk of adverse drug reactions. Preventative medications, such as those used to manage blood pressure and cholesterol, are common in polypharmacy and often require large numbers of people to be treated to prevent a small number of cardiovascular disease (CVD) events. This leaves many individuals on drugs of little benefit, some of whom may be susceptible to side effects such as falls, kidney problems and muscle pain.  *Aims*  This proposal aims to quantify the harms of cardiovascular prevention medication, and the characteristics of those people most likely to suffer them. This research is one part of a larger research programme to develop a clinical decision tool which estimates an individual’s likelihood of benefiting or suffering harm from treatment.    *Methods*  Aim 1: Derive prognostic models for an individual’s risk of adverse events associated with cardiovascular prevention treatment (antihypertensives, statins and antiplatelets) using data from the CPRD GOLD. Adverse event outcomes will include falls (antihypertensives), myopathy (statins) and bleeding (antiplatelets).  Aim 2: Externally validate each model using data from the CPRD Aurum.  Aim 3: Use causal inference methods (propensity score matching, instrumental variable analysis) to examine whether modification of treatment could have an important impact on the risk of adverse events. |

| 1. **Outcomes to be measured**   **Primary outcomes** - Falls; Myopathy/muscle pain; Intracerebral haemorrhage  **Secondary outcomes** – Fracture; Syncope; Hypotension (symptomatic); Acute kidney injury; Electrolyte abnormalities; Gout; Diabetes mellitus; Intracerebral haemorrhage; Cataract; Liver dysfunction; Dementia; Memory problems (including dementia); Gastrointestinal haemorrhage; Dyspnoea; Any adverse event  Specific outcomes will be examined in relation to exposure to either antihypertensives, statins or antiplatelets (details given in section N). |
| --- |
| 1. **Objectives, Specific Aims and Rationale**   *Objective*  The overarching objective of this study is to better understand the harms of preventative treatments (antihypertensives, statins and antiplatelets) by quantifying an individual’s baseline risk of harm and the modifying effect of treatment.    *Specific aims*  Aim 1: Derive prognostic models for an individual’s risk of adverse events associated with cardiovascular prevention treatment (antihypertensives, statins and antiplatelets). Adverse event outcomes will include falls (antihypertensives), myopathy (statins) and bleeding (antiplatelets).  Aim 2: Externally validate each model.  Aim 3: Use causal inference methods (propensity score matching, instrumental variable analysis) to examine whether modification of treatment could have an important impact on the risk of adverse events.  *Rationale*  The proposed work will develop and validate new prediction tools for an individual’s risk of harms from cardiovascular prevention medication. This information will be combined with existing tools for the benefits of treatment and be used to form a new strategy that better targets preventative therapy at those with the most to gain. By empowering patients and clinicians to better understand the risks of preventative medications, this work will promote a more patient-centred, shared-decision making approach to cardiovascular disease prevention in primary care. |
| 1. **Study Background**   Cardiovascular disease (CVD) is the leading cause of mortality worldwide.^1^ As a result, much healthcare resource is assigned to preventing CVD through modification of risk factors, such as raised blood pressure and/or cholesterol.^2^ This can be achieved through prescription of medications such as antihypertensives, statins and antiplatelets, which have been shown to be effective in reducing the risk of cardiovascular disease.^3-5^ These medications are often started when patients are at low-to-moderate risk and continued for many years, despite a low likelihood of benefit.^6^ Some patients who take these medications may suffer side effects such as falls, acute kidney injury (AKI), muscle pain and bleeding which can significantly reduce an individual’s quality of life, particularly those who are old and frail.^7-9^ Despite changes in an individual’s risk/benefit profile, clinicians are often reluctant to stop prescribing them.^10^  The association between cardiovascular preventative therapy and adverse events is not fully understood. This is due in part, to the completeness of reporting of side effects and adverse events in previous trials.^11 12^ Observational studies do show an association between treatment and adverse outcomes such as falls,^9 13 14^ AKI,^15^ myopathy,^16^ diabetes,^17^ and intracranial bleeding,^18^ but are limited in some cases due to small sample sizes and bias caused by unmeasured confounding. Observational data can however be useful since it allows multi-morbid populations to be examined, which are more representative of the general population than those enrolled into clinical trials.^19^  *Predicting the benefits and harms of treatment*  A structured approach to weighing up the risks and benefits of treatment is common in atrial fibrillation, where combining risk scores from the CHA_2_DS_2_-VASc tool^20^ and HAS-BLED tool^21^ allows one to compare an individual’s risk of stroke with their risk of a bleed. There are many other risk prediction tools which can be used to identify individuals who may benefit from CVD prevention treatment (i.e. are at high risk of CVD),^22-28^ but very few which can be used to identify those at risk of adverse events in the community. Existing tools show moderate discrimination and focus on patients with specific conditions and/or use in an acute hospital setting.^29-34^ None have been externally validated.^35-37^ Recent studies have described prediction models for the benefits and harms of *intensive* blood pressure lowering treatment,^38 39^ but these were derived exclusively from patients in the SPRINT trial,^7^ limiting their generalisability.  Stratifying treatments for prevention of CVD is important for UK and international health. Such an approach could facilitate shared decision making between patients and doctors and allow better targeting of treatments in settings where resources and access to drugs may be limited.  *How this project fits in*  This is one project in a larger work programme aiming to personalise preventative treatments for cardiovascular disease. In this project, we will quantify risks due to cardiovascular preventive treatments. In other projects, including systematic reviews, we will quantify and combine estimates of the benefits and harms of preventive treatments. This will enable us to develop a clinical decision tool to assist patients and their clinicians in understanding the relative benefits and harms of proposed treatment changes. |
| 1. **Study Type**   Hypothesis testing |
| 1. **Study Design**   Longitudinal cohort study |
| 1. **Feasibility counts**   There are approximately 5.5 million patients in England, aged 40+ and with up to standard registration for a period between 1/1/1998 to 31/12/2017 in the CPRD GOLD. Restricting to English practices (linked to HES and ONS) and only including patients fulfilling the eligibility criteria listed below, the expected population is approximately 3.3 million and out of these we expect 216,000 fall events, 230,000 myopathy/muscle pain events and 1,200 intracranial haemorrhage events. |
| 1. **Sample size considerations**   We have based our sample size calculation on the least common of the primary outcomes listed above (intracranial haemorrhage), and used the method developed by Riley *et al.,*^40^ to minimise the potential for overfitting and ensure precise estimates of key parameters. A sample size of approximately 80,000 patients is required for the development of a clinical prediction rule for this outcome, assuming an event rate of 24.6 per 100,000 person years,^41^ a median follow up of 7 years,^42^ a conservative estimate of Nagelkerke’s R^2^ statistic of 0.15 and a maximum number of 40 parameters per model.  Feasibility counts showed that this sample size is adequate for the development of the prediction rules. Since Intracranial bleeding is the rarest of the primary outcomes to be studied, the sample size will be sufficient for all other outcomes of interest. For validation, Vergouwe *et al.,*^43^ consider at least 100 events per sample population to be “substantial” and we will comfortably exceed this in our validation cohorts for each outcome of interest.  For analyses using causal inference methods (propensity score matching and instrumental variable analysis), assuming clinically significant increased rate of each adverse event with treatment of 10%, and an event rate of at least 0.5% per year in the non-exposed group, approximately 88,380 patients (44,190 in each group) and 4,634 events will be required to accurately define the relationship between preventative treatment and adverse events, with 90% power and an alpha of 0.05. |
| 1. **Planned use of linked data (if applicable):**   Data linkage to the ONS and Basic HES are required to define the primary and secondary outcomes in the study. The ONS mortality register will be used to define any outcomes which result in death, and also censor follow-up at death. Specific linkages required will include data and ICD-10 coded cause of death (see attached code lists). Linkages to Basic Inpatient HES will be combined with data from the ONS to define all other outcomes in the study. HES data will also be used to define patients’ eligibility for the study (e.g. previous CVD), and define the study population (e.g. ethnicity where unavailable in Primary Care records). Data required from Basic Inpatient HES will include primary diagnosis, secondary diagnosis, patient characteristics (e.g. sex, ethnicity), date of admission and date of discharge. All deaths and hospital admissions occurring after a patient’s index date will be included. A linkage to the Index of Multiple Deprivation is required to acquire patient level quintiles of multiple deprivation, to better define the sample population and use as a covariate in the prognostic modelling. |
| 1. **Definition of the Study population**   Individual patient data will be extracted from the medical records of all patients registered at linked general practices contributing to the CPRD GOLD and Aurum in England.  Patients will be included if they meet the following criteria:   - Patients over the age of 40 years - Registered to a CPRD ‘up-to-standard’ practice - Records available after the study start date (01/01/1998) - Sufficient data to define the index date (see table below)   Patients will be excluded if they meet the following criteria:   - Previous prescription of the exposure variable (antihypertensive, statin or antiplatelet) - Blood pressure >180 mm Hg or total cholesterol >7.5 mmol/L (in patients with high blood pressure or cholesterol, treatment is indicated regardless of risk)   Study entry criteria – Patients will enter the cohort on the index date, defined as:   \| **Exposure** \| Antihypertensive therapy \| Statin therapy \| Antiplatelet therapy \| \| --- \| --- \| --- \| --- \| \| **Index date definition** \| 12 months after the first systolic blood pressure reading ≥130 mm Hg \| 12 months after the first coded or calculated CVD risk of ≥5% \| 3 months after first CVD event \|   Study exit criteria   - Last date at which the most recent linked data are available from the CPRD (study end date, July 2018) - Date of the most recent data upload from the practice to which a given patient is registered - Date at which a given patient transfers out of a registered CPRD practice - Date of death or specific outcome of interest |
| 1. **Selection of comparison group(s) or controls**   Aim 1: Not applicable – single cohort in CPRD GOLD.  Aim 2: Not applicable – single cohort in CPRD AURUM.  Aim 3 will use propensity score matching and instrumental variable analysis – see Methods section below. |
| 1. **Exposures, Outcomes and Covariates**   This study will examine the overall risk of harm from three types of cardiovascular medication: antihypertensives, statins and antiplatelets. ‘Harm’ will be defined by the outcomes listed in the table below, which will be examined both individually and as a composite outcome. The association between treatment and a positive and negative control outcome will also be examined to check the validity of the treatment effect estimates. Positive controls are outcomes know to be affected by treatment (e.g. cardiovascular disease) and negative outcomes are those known not to be affected by treatment.   \| **Variable** \| **Antihypertensive therapy** \| **Statin therapy** \| **Antiplatelet therapy** \| \| --- \| --- \| --- \| --- \| \| Exposure \| ACE inhibitors  Angiotensin II receptor antagonists  Calcium channel blockers  Thiazides and thiazide-like diuretics  Beta blockers  Alpha blockers  Centrally acting antihypertensives  Vasodilators  Renin inhibitors \| Statins \| Aspirin  Clopidogrel  Dipyridamole  Ticagrelor  Prasugrel \| \| Outcome (hospital admission or death due to) \| Falls  Fracture  Syncope  Hypotension (symptomatic)  Acute kidney injury  Electrolyte abnormalities*  Gout  Any adverse event \| Myopathy/muscle pain  Diabetes mellitus  Intracerebral haemorrhage  Cataract  Liver dysfunction  Dementia  Memory problems (including dementia)  Any adverse event \| Intracerebral haemorrhage  Gastrointestinal haemorrhage  Dyspnoea  Any adverse event \| \| Covariates (predictors of outcome and/or treatment) \| Age, sex, BMI, blood pressure, cholesterol, HbA1c, other prescribed medications, co-morbidities, social deprivation, previous adverse events, frailty and proximity to death. \| \| \|   *Defined as any (hyponatraemia, hypokalaemia, hyperkalaemia, hypomagnesaemia, hypophosphataemia hypercalcaemia or hyperuricemia)  All outcomes will be defined according to diagnostic and/or symptom codes, unless otherwise stated. Further sensitivity analyses will explore the impact of our definition of myopathy and muscle pain, focussing on formal diagnostic codes for myopathy only.  Our PPI engagement suggests some patients are concerned about side effects which will result in having to go to hospital, whilst others are more concerned about chronic conditions which affect their daily quality of life. The primary outcomes of this study will therefore be hospital attendance, defined according to ONS and Basic HES datasets. Sensitivity analyses will explore outcomes defined more broadly using primary care codes as well.  All published outputs from this work will be based on the outcomes as specified in this application. Further work with our patient and public involvement representatives will explore definitions of these outcomes and how they should be incorporated into the final calculators developed as part of the wider programme of work related to this project.  Each model will be derived pragmatically, using coded data routinely available in a primary care setting. Potential predictors will be selected based on previous literature and expert opinion.^30 31 44^ Predictors will be defined by codes in the primary care records, using code lists from previous studies (via [www.clinicalcodes.org](http://www.clinicalcodes.org)). Each model will also incorporate basic patient characteristics, blood pressure level, co-morbidities and frailty, estimated from the electronic frailty index. |
| 1. **Data/ Statistical Analysis**   **Prognostic modelling**  ***Aim 1 – prognostic model derivation (primary analysis)***  Where feasible and appropriate, flexible parametric survival models will be derived in the CPRD GOLD for each outcome separately. Baseline survival over time will be estimated using the Royston-Parmar approach of restricted cubic splines on the cumulative log hazard scale. In contrast to Cox regression, this allows a smoothed estimate of the baseline survival function to be derived, and the time period of prediction to be varied, facilitating individualised risk prediction. For the primary analysis, a 10-year period of prediction will be considered. Further sensitivity analyses will be conducted examining a 5-year period. Where insufficient data are available, or modelling procedures become prohibitively complex (computationally), simpler approaches such as Cox regression will be considered.  Each drug class (prescribed in the 12 months prior to the index date) will be entered into the model as a binary variable to allow for multiple treatments to be considered in each model. Where appropriate, models will be constructed with all other variables entered as continuous (not categorised) variables, and potential non-linear trends will be examined. Transformations or fractional polynomials will be used if non-linear trends are detected. Where hazard ratios are not proportional, interactions of effects with time will be included. Further interaction terms, identified in an accompanying systematic review, accounting for treatment effects which differ across specific populations may be included. Where appropriate, models will be reduced and candidate predictors selected for inclusion based on p values of association. Time since cohort entry will be used as the underlying time function in each survival model.  In a further analysis, we will extend the modelling process as described above to account for competing risk of death from other causes. This will produce a model that predicts the risk of adverse events from cardiovascular treatment over time, in the real world where individuals might die from other causes and thus prevent cardiovascular treatment adverse events from occurring. We will again use a flexible parametric modelling framework to estimate the sub-distribution hazard and cause-specific cumulative incidence functions.^45^  Missing data will be handled, under a missing at random assumption, using multiple imputation (see below) followed by Rubin’s rules to combine parameter estimates across studies.  Where feasible and appropriate, each model will be internally validated using the bootstrap method, to allow optimism-adjusted estimates of calibration and discrimination performance to be obtained. Adjustment for overfitting will be undertaken using a uniform shrinkage factor identified from bootstrapping, followed by re-estimation of the cumulative baseline sub-distribution hazard function (and thus baseline cumulative incidence function) to maintain overall calibration between observed and predicted risks. Given the large sample size, overfitting is likely to be small (see sample size calculation above). If bootstrapping is computationally infeasible due to the large sample size, then a smaller random sample of the population will be used or alternative internal validation methods employed such as cross validation.  ***Aim 2 – prognostic model validation***  External model validation will be undertaken using data from CPRD Aurum. The accuracy of risk predictions from each model will be assessed with calibration plots and 5 and 10 years, calibration measures (slope, E/O statistics), measures of overall fit (using pseudo-R^2^ estimates) and discrimination measures (C-statistics and D-statistics).  ***Aim 3 – causal inference analysis***  For most of the outcomes described in this proposal, the association and magnitude of effect with treatment is not well defined in randomised controlled trials. We will therefore undertake causal inference work to examine whether modification of treatment could have an important impact on the risk of adverse events using two causal inference methods: propensity score matching and instrumental variable analysis.  For the propensity score analysis, eligible patients will be matched 1:1 at the index date using propensity scores, which indicate the likelihood a patient will be prescribed treatment on the basis of their known (pre-treatment) characteristics and other known information which might influence the decision to treat. Predictors of preventative drug prescription will be explored in a logistic regression model. Matched patients will be compared using the Cox proportional hazards models, but hazard ratios will only be adjusted by factors unbalanced at baseline which are not already included in the propensity score model. Time since cohort entry will be used as the underlying time function in each model.  For the instrumental variable analysis, GP’s previous antihypertensive prescribing preferences will be used as an instrument to predict the likelihood of actual treatment. Risk of adverse events will be estimated in patients attending a GP considered a high prescriber of preventative medication and compared to those attending a GP considered a low prescriber. If the results from the propensity score matching suffer from unmeasured confounding, they will differ from those of the instrumental analysis. If this is the case, these models will be refined with additional factors to achieve better adjustment and matching. Where all approaches provide similar results, we can be more confident that the findings are accurate and reliable.^46^  A similar approach will be applied to secondary outcomes, for example, memory loss which has been hypothesised as being linked with statins^47^ and is a concern of many patients in practice, but there is no evidence to support this association from trials.^48^ We recognise that the evidence base may change for some outcomes over the next few years, and so the need to test the associations between treatment and certain outcomes may change.  *Subgroup and sensitivity analyses*  Where possible and appropriate, analyses of treatment associations will be examined in subgroups of the population. These will include age, sex, baseline blood pressure and cardiovascular risk, by drug type/dose/intensity and past medical history. Subgroup definitions will be agreed by expert opinion or taken from an accompanying systematic review of previous trials, examining the same topic.  Sensitivity analyses will be undertaken examining risk models for outcomes defined according to both hospital and primary care records. We will also explore the impact of our definition of myopathy and muscle pain, focussing on formal diagnostic codes for myopathy only. Models utilising a 5-year follow-up will be examined as a further sensitivity analysis. The primary analyses will examine the association between treatment and outcomes, assuming treatments are not altered or modified in the future. Where possible, sensitivity analyses will examine treatment entered as time varying covariates, and censor patients who have treatments removed or switched following the index date. |
| 1. **Plan for addressing confounding**   Two approaches will be taken to determine whether observed associations are potentially subject to residual confounding.^46^ Where all approaches provide similar results, we can be more confident that the findings are accurate and reliable. Further steps will be taken to examine the validity of treatment effect estimates using positive and negative controls: the impact of treatment on an outcome known to be affected by treatment (e.g. cardiovascular disease; positive control) and outcomes not known to be affected by treatment (negative control). Here, we will combine two common but unrelated outcomes to be examined as negative controls: bowel cancer and chronic obstructive pulmonary disease (COPD). If treatment has a significant impact on these negative controls, it suggests that there is something missing in the analysis (i.e. an unmeasurable factor confounding the treatment effect such as being generally unwell or an unhealthy lifestyle) causing an imbalance between the treatment and control groups, rather than a true treatment effect. |
| 1. **Plans for addressing missing data**   There is potential for missing data in this study, particularly with variables such as ethnicity which are recorded with varying degrees of accuracy in routine practice. Because this analysis is focused on treatment for prevention of cardiovascular disease, accurate selection of the sample population is important and therefore any patients with insufficient data available to define their blood pressure or cardiovascular risk status at baseline (index date) will be excluded. Where there is no record of blood pressure lowering, statin or antiplatelet treatment, it will be assumed the patients were not exposed to blood pressure lowering, statin or antiplatelet treatment.  Patient eligibility includes only using ‘acceptable patients’ in the analysis, and therefore there is no need to impute age and sex. Where there is no record of smoking history or alcohol consumption, patients will be assumed to be non-smokers and non-drinkers. Likewise, those with no record of co-morbidities will be assumed to have no history of these conditions. All other covariates (including BMI, ethnicity and IMD) used in the prognostic or casual inference modelling will be imputed using multiple imputation. BMI and will be treated as a continuous variable.  All analyses of treatment associations with outcomes will be conducted by intention-to-treat, and patients moving practice after the index date (and therefore being lost to follow-up) will be censored at the point at which they are no longer active in the database. |
| 1. **Patient or user group involvement (if applicable)**   This application has been discussed with patients (aged 67-86 years, taking 5-11 meds), a carer who also takes multiple meds takes 5 meds and the AgeUK Bakewell day centre (group discussion with 18 frail elderly persons).  Key issues raised   - Individuals vary widely on the amount of information they wish to know about their medications, and this is often influenced by the doctor-patient relationship. - Patients sometimes rely on unclear or unreliable information about the potential harms of their treatments from medication packets, the internet or friends. - Some patients are concerned about suffering events which will cause them to go to hospital, but most focus on the ‘here-and-now’; chronic issues which affect their quality of life (e.g. pain limiting mobility). - Some do not trust their GPs understanding of medications and prefer to see a specialist pharmacist with more time to consider their needs. Others who see the same GP regularly are happy to ‘do as they are told,’ but assume the GP fully understands the benefits and harms of the treatments they take. - Patients are happy to be involved in research, but location and access is very important, and a multifaceted approach to engagement is required. Some are more comfortable with technology than others. - Older individuals said they were happier having (potentially sensitive) PPI discussions with someone they knew and trusted.   Project areas directly influenced by PPI  Overarching aim: Some individuals wish to know about the benefits and harms of medications whilst others expect the GP will fully understand the risks. A clinical decision support tool is needed to improve the understanding of both patients and their GPs.  Model outcomes: Patient’s opinions vary in the outcomes that concern them the most and so this proposal focusses on both serious acute events and also chronic problems which can affect daily life. Advice will be sought as to how these outcomes should be defined in the final calculators.  PPI engagement: A variety of methods of PPI engagement are required to maximise access for patients. Engaging with relatives and familiar members of local churches and community groups will be important. |
| 1. **Plans for disseminating and communicating study results, including the presence or absence of any restrictions on the extent and timing of publication**   All findings from the proposed research project will be published in peer-reviewed scientific journals. Findings will be presented at national and international conferences in Primary Care (e.g. Society for Academic Primary Care [SAPC], North American Primary Care Research Group), Hypertension (British and Irish Hypertension Society [BIHS], European Society for Hypertension) and cardiovascular disease (European Society of Cardiology).  An article discussing the issues raised by the research will be written for the online newspaper ‘The Conversation’, and this will also be posted on the Nuffield Department of Primary Care Health Sciences website as an online blog. Where appropriate, results of the research will be press-released in combination with their publication in scientific journals. Social media (twitter) will be used to draw further attention to the work.  The results of this work will be used to develop a calculator which estimates an individual’s risk of suffering harm from preventative treatments for cardiovascular disease. The risk calculator will be made freely available online for use by patients and practitioners.  **Conflict of interest statement:** The authors declare no conflicts of interest |
| 1. **Limitations of the study design, data sources, and analytic methods**   This study focuses on potential harms from adverse events associated with preventative treatments for cardiovascular disease. Such outcomes may be less well recorded in routine electronic health records. For example, initial scoping work suggests the rate of falls documented in the CPRD is between 0.06-0.34% per 1000 person-months. These incidence rates may be less than those reported in smaller previous studies (0.15-6.40% per 1000 person-months) using different definitions, captured via detailed questionnaires and follow-up.^49-52^ Incidence rates are likely to be higher when linked data from HES and ONS are available.^53^ Missing data on certain outcomes may reduce the accuracy of the prognostic models, but should not bias the results provided the data are missing at random and not affected by whether patients are prescribed treatment. To minimise the risk of reporting bias, outcomes in the primary analysis will be defined according to hospital admissions documented in the HES or ONS.  The causal inference work will use an observational cohort design, and as such, there is an inherent selection bias of patients in both exposure and control groups. The impact of this bias will be limited by propensity score methods used to control for confounding, but this approach assumes that all confounding factors are measured and accounted for within the analysis. In the present study, we will examine the validity of this assumption by 1) using an instrumental variable (GP prescriber preference) and examining the association with the outcomes of interest and 2) by studying the impact of treatment on positive and negative controls: hospitalisation and/or death from cardiovascular disease and bowel cancer/COPD. We will conclude the assumption has been met if high GP prescribers have a similar association with outcomes and the treatment itself and treatment is shown to be associated with the positive control, but not the negative control.  *Strategy for dealing with potential errors resulting from multiple testing*  This study will examine multiple outcomes across 3 exposure variables. To avoid potential errors arising from multiple testing, a primary outcome has been clearly defined for each exposure variable. Secondary outcomes are also pre-specified. The primary outcome will be given priority in the final analysis write-up and any related reports and presentations.  Primary and secondary outcomes will be defined using data from Basic inpatient HES and ONS. Sensitivity analyses will explore analyses defining these outcomes using the above sources and read-coded primary care data. The accuracy of such outcome data has been examined previously in patients with acute myocardial infarction and whilst recording of risk factor and co-morbid information was consistent across primary care, hospital admissions and disease registry records, the crude incidence of acute myocardial infarction was underestimated by up to 50% if only one data source was used, compared with using all three sources.^53^ The use of linked CPRD data will ensure outcome data are ascertained accurately. |
| 1. **References**   1. Lozano R, Naghavi M, Foreman K, et al. Global and regional mortality from 235 causes of death for 20 age groups in 1990 and 2010: a systematic analysis for the Global Burden of Disease Study 2010. *Lancet* 2012;380(9859):2095-128. doi: 10.1016/s0140-6736(12)61728-0 [published Online First: 2012/12/19]  2. NatCen Social Research. Health Survey for England 2014. <http://digital.nhs.uk/catalogue/PUB19297>, 2015.  3. Chou R, Dana T, Blazina I, et al. Statins for Prevention of Cardiovascular Disease in Adults: Evidence Report and Systematic Review for the US Preventive Services Task Force. *Jama* 2016;316(19):2008-24. doi: 10.1001/jama.2015.15629 [published Online First: 2016/11/14]  4. Ettehad D, Emdin CA, Kiran A, et al. Blood pressure lowering for prevention of cardiovascular disease and death: a systematic review and meta-analysis. *Lancet* 2016;387(10022):957-67. doi: 10.1016/s0140-6736(15)01225-8 [published Online First: 2016/01/03]  5. Collaborative meta-analysis of randomised trials of antiplatelet therapy for prevention of death, myocardial infarction, and stroke in high risk patients. *BMJ (Clinical research ed)* 2002;324(7329):71-86. [published Online First: 2002/01/12]  6. Sheppard JP, Stevens S, Stevens R, et al. Benefits and Harms of Antihypertensive Treatment in Low-Risk Patients With Mild Hypertension. *JAMA internal medicine* 2018;178(12):1626-34. doi: 10.1001/jamainternmed.2018.4684 [published Online First: 2018/11/02]  7. Wright JT, Jr., Williamson JD, Whelton PK, et al. A Randomized Trial of Intensive versus Standard Blood-Pressure Control. *The New England journal of medicine* 2015;373(22):2103-16. doi: 10.1056/NEJMoa1511939 [published Online First: 2015/11/10]  8. Callisaya ML, Sharman JE, Close J, et al. Greater daily defined dose of antihypertensive medication increases the risk of falls in older people--a population-based study. *J Am Geriatr Soc* 2014;62(8):1527-33. doi: 10.1111/jgs.12925 [published Online First: 2014/06/18]  9. Tinetti ME, Han L, Lee DS, et al. Antihypertensive medications and serious fall injuries in a nationally representative sample of older adults. *JAMA internal medicine* 2014;174(4):588-95. doi: 10.1001/jamainternmed.2013.14764 [published Online First: 2014/02/26]  10. Sinnott C, Hugh SM, Boyce MB, et al. What to give the patient who has everything? A qualitative study of prescribing for multimorbidity in primary care. *The British journal of general practice : the journal of the Royal College of General Practitioners* 2015;65(632):e184-91. doi: 10.3399/bjgp15X684001 [published Online First: 2015/03/04]  11. Ioannidis JP, Lau J. Completeness of safety reporting in randomized trials: an evaluation of 7 medical areas. *Jama* 2001;285(4):437-43. [published Online First: 2001/03/10]  12. Stevens PE, Lamb EJ, Levin A. Integrating guidelines, CKD, multimorbidity, and older adults. *American journal of kidney diseases : the official journal of the National Kidney Foundation* 2015;65(3):494-501. doi: 10.1053/j.ajkd.2014.09.024 [published Online First: 2014/12/09]  13. Gribbin J, Hubbard R, Gladman JR, et al. Risk of falls associated with antihypertensive medication: population-based case-control study. *Age and ageing* 2010;39(5):592-7. doi: 10.1093/ageing/afq092 [published Online First: 2010/07/24]  14. Butt DA, Mamdani M, Austin PC, et al. The risk of falls on initiation of antihypertensive drugs in the elderly. *Osteoporosis international : a journal established as result of cooperation between the European Foundation for Osteoporosis and the National Osteoporosis Foundation of the USA* 2013;24(10):2649-57. doi: 10.1007/s00198-013-2369-7 [published Online First: 2013/04/25]  15. Mansfield KE, Nitsch D, Smeeth L, et al. Prescription of renin–angiotensin system blockers and risk of acute kidney injury: a population-based cohort study. *BMJ open* 2016;6(12) doi: 10.1136/bmjopen-2016-012690  16. Hippisley-Cox J, Coupland C. Unintended effects of statins in men and women in England and Wales: population based cohort study using the QResearch database. *BMJ (Clinical research ed)* 2010;340:c2197. doi: 10.1136/bmj.c2197 [published Online First: 2010/05/22]  17. Macedo AF, Taylor FC, Casas JP, et al. Unintended effects of statins from observational studies in the general population: systematic review and meta-analysis. *BMC medicine* 2014;12:51. doi: 10.1186/1741-7015-12-51 [published Online First: 2014/03/25]  18. Garcia Rodriguez LA, Martin-Perez M, Hennekens CH, et al. Bleeding Risk with Long-Term Low-Dose Aspirin: A Systematic Review of Observational Studies. *PLoS ONE* 2016;11(8):e0160046. doi: 10.1371/journal.pone.0160046 [published Online First: 2016/08/05]  19. Starfield B. New paradigms for quality in primary care. *The British journal of general practice : the journal of the Royal College of General Practitioners* 2001;51(465):303-9.  20. Lip GY, Nieuwlaat R, Pisters R, et al. Refining clinical risk stratification for predicting stroke and thromboembolism in atrial fibrillation using a novel risk factor-based approach: the euro heart survey on atrial fibrillation. *Chest* 2010;137(2):263-72. doi: 10.1378/chest.09-1584 [published Online First: 2009/09/19]  21. Pisters R, Lane DA, Nieuwlaat R, et al. A novel user-friendly score (HAS-BLED) to assess 1-year risk of major bleeding in patients with atrial fibrillation: the Euro Heart Survey. *Chest* 2010;138(5):1093-100. doi: 10.1378/chest.10-0134 [published Online First: 2010/03/20]  22. Damen JA, Hooft L, Schuit E, et al. Prediction models for cardiovascular disease risk in the general population: systematic review. *BMJ (Clinical research ed)* 2016;353:i2416. doi: 10.1136/bmj.i2416 [published Online First: 2016/05/18]  23. Anderson KM, Odell PM, Wilson PW, et al. Cardiovascular disease risk profiles. *American heart journal* 1991;121(1 Pt 2):293-8. [published Online First: 1991/01/01]  24. D'Agostino RB, Sr., Vasan RS, Pencina MJ, et al. General cardiovascular risk profile for use in primary care: the Framingham Heart Study. *Circulation* 2008;117(6):743-53. doi: 10.1161/circulationaha.107.699579 [published Online First: 2008/01/24]  25. Woodward M, Brindle P, Tunstall-Pedoe H. Adding social deprivation and family history to cardiovascular risk assessment: the ASSIGN score from the Scottish Heart Health Extended Cohort (SHHEC). *Heart (British Cardiac Society)* 2007;93(2):172-6. doi: 10.1136/hrt.2006.108167 [published Online First: 2006/11/09]  26. Hippisley-Cox J, Coupland C, Vinogradova Y, et al. Predicting cardiovascular risk in England and Wales: prospective derivation and validation of QRISK2. *BMJ (Clinical research ed)* 2008;336(7659):1475-82. doi: 10.1136/bmj.39609.449676.25 [published Online First: 2008/06/25]  27. Joint British Societies. Joint British Societies' consensus recommendations for the prevention of cardiovascular disease (JBS3). *Heart (British Cardiac Society)* 2014;100 Suppl 2:ii1-ii67. doi: 10.1136/heartjnl-2014-305693 [published Online First: 2014/03/29]  28. Hippisley-Cox J, Coupland C, Brindle P. Development and validation of QRISK3 risk prediction algorithms to estimate future risk of cardiovascular disease: prospective cohort study. *BMJ (Clinical research ed)* 2017;357:j2099. doi: 10.1136/bmj.j2099 [published Online First: 2017/05/26]  29. Birnie K, Verheyden V, Pagano D, et al. Predictive models for kidney disease: improving global outcomes (KDIGO) defined acute kidney injury in UK cardiac surgery. *Critical care (London, England)* 2014;18(6):606. doi: 10.1186/s13054-014-0606-x [published Online First: 2015/02/13]  30. Englberger L, Suri RM, Li Z, et al. Validation of clinical scores predicting severe acute kidney injury after cardiac surgery. *American journal of kidney diseases : the official journal of the National Kidney Foundation* 2010;56(4):623-31. doi: 10.1053/j.ajkd.2010.04.017 [published Online First: 2010/07/16]  31. Hodgson LE, Dimitrov BD, Roderick PJ, et al. Predicting AKI in emergency admissions: an external validation study of the acute kidney injury prediction score (APS). *BMJ open* 2017;7(3):e013511. doi: 10.1136/bmjopen-2016-013511 [published Online First: 2017/03/10]  32. Wang YN, Cheng H, Yue T, et al. Derivation and validation of a prediction score for acute kidney injury in patients hospitalized with acute heart failure in a Chinese cohort. *Nephrology (Carlton, Vic)* 2013;18(7):489-96. doi: 10.1111/nep.12092 [published Online First: 2013/04/24]  33. Hippisley-Cox J, Coupland C. Derivation and validation of updated QFracture algorithm to predict risk of osteoporotic fracture in primary care in the United Kingdom: prospective open cohort study. *BMJ (Clinical research ed)* 2012;344:e3427. doi: 10.1136/bmj.e3427 [published Online First: 2012/05/24]  34. Kanis JA, Johnell O, Oden A, et al. FRAX™ and the assessment of fracture probability in men and women from the UK. *Osteoporosis international : a journal established as result of cooperation between the European Foundation for Osteoporosis and the National Osteoporosis Foundation of the USA* 2008;19(4):385-97. doi: 10.1007/s00198-007-0543-5  35. Jennings LA, Reuben DB, Kim SB, et al. Targeting a high-risk group for fall prevention: strategies for health plans. *American Journal of Managed Care* 2015;21(9):e519-26.  36. Stalenhoef PA, Diederiks JP, Knottnerus JA, et al. A risk model for the prediction of recurrent falls in community-dwelling elderly: a prospective cohort study. *Journal of Clinical Epidemiology* 2002;55(11):1088-94.  37. Woo J, Leung J, Wong S, et al. Development of a simple scoring tool in the primary care setting for prediction of recurrent falls in men and women aged 65 years and over living in the community. *Journal of Clinical Nursing* 2009;18(7):1038-48.  38. Basu S, Sussman JB, Rigdon J, et al. Benefit and harm of intensive blood pressure treatment: Derivation and validation of risk models using data from the SPRINT and ACCORD trials. *PLoS medicine* 2017;14(10):e1002410. doi: 10.1371/journal.pmed.1002410 [published Online First: 2017/10/19]  39. Patel KK, Arnold SV, Chan PS, et al. Personalizing the Intensity of Blood Pressure Control: Modeling the Heterogeneity of Risks and Benefits From SPRINT (Systolic Blood Pressure Intervention Trial). *Circulation Cardiovascular quality and outcomes* 2017;10(4) doi: 10.1161/circoutcomes.117.003624 [published Online First: 2017/04/05]  40. Riley RD, Snell KIE, Ensor J, et al. Minimum sample size for developing a multivariable prediction model: Part I - Continuous outcomes. *Stat Med* 2018 doi: 10.1002/sim.7993 [published Online First: 2018/10/23]  41. van Asch CJ, Luitse MJ, Rinkel GJ, et al. Incidence, case fatality, and functional outcome of intracerebral haemorrhage over time, according to age, sex, and ethnic origin: a systematic review and meta-analysis. *The Lancet Neurology* 2010;9(2):167-76. doi: 10.1016/s1474-4422(09)70340-0 [published Online First: 2010/01/09]  42. Stevens SL, McManus RJ, Stevens RJ. The utility of long-term blood pressure variability for cardiovascular risk prediction in primary care. *J Hypertension* 2018 doi: 10.1097/hjh.0000000000001923 [published Online First: 2018/09/21]  43. Vergouwe Y, Steyerberg EW, Eijkemans MJ, et al. Substantial effective sample sizes were required for external validation studies of predictive logistic regression models. *J Clin Epidemiol* 2005;58(5):475-83. doi: 10.1016/j.jclinepi.2004.06.017 [published Online First: 2005/04/23]  44. National Guideline Centre. National Institute for Health and Care Excellence: Clinical Guidelines. Falls in older people: assessing risk and prevention [Clinical Guideline 161]. London: National Institute for Health and Care Excellence (UK)  Copyright (c) National Institute for Health and Care Excellence, 2016. 2013.  45. Lambert PC, Wilkes SR, Crowther MJ. Flexible parametric modelling of the cause-specific cumulative incidence function. *Stat Med* 2017;36(9):1429-46. doi: 10.1002/sim.7208 [published Online First: 2016/12/23]  46. Taylor GMJ, Taylor AE, Thomas KH, et al. The effectiveness of varenicline versus nicotine replacement therapy on long-term smoking cessation in primary care: a prospective cohort study of electronic medical records. *International journal of epidemiology* 2017;46(6):1948-57. doi: 10.1093/ije/dyx109 [published Online First: 2017/10/19]  47. Strom BL, Schinnar R, Karlawish J, et al. Statin Therapy and Risk of Acute Memory Impairment. *JAMA internal medicine* 2015;175(8):1399-405. doi: 10.1001/jamainternmed.2015.2092 [published Online First: 2015/06/09]  48. Collins R, Reith C, Emberson J, et al. Interpretation of the evidence for the efficacy and safety of statin therapy. *Lancet* 2016;388(10059):2532-61. doi: 10.1016/s0140-6736(16)31357-5 [published Online First: 2016/09/13]  49. Ali T, Khan I, Simpson W, et al. Incidence and outcomes in acute kidney injury: a comprehensive population-based study. *Journal of the American Society of Nephrology : JASN* 2007;18(4):1292-8. doi: 10.1681/asn.2006070756 [published Online First: 2007/02/23]  50. Barton AL, Mallard AS, Parry RG. One Year's Observational Study of Acute Kidney Injury Incidence in Primary Care; Frequency of Follow-Up Serum Creatinine and Mortality Risk. *Nephron* 2015;130(3):175-81.  51. Stalenhoef PA, Diederiks JP, de Witte LP, et al. Impact of gait problems and falls on functioning in independent living persons of 55 years and over: a community survey. *Patient education and counseling* 1999;36(1):23-31. [published Online First: 1999/02/26]  52. Tinetti ME, Speechley M, Ginter SF. Risk factors for falls among elderly persons living in the community. *The New England journal of medicine* 1988;319(26):1701-7. doi: 10.1056/nejm198812293192604 [published Online First: 1988/12/29]  53. Herrett E, Shah AD, Boggon R, et al. Completeness and diagnostic validity of recording acute myocardial infarction events in primary care, hospital care, disease registry, and national mortality records: cohort study. *BMJ (Clinical research ed)* 2013;346 doi: 10.1136/bmj.f2350 |
| 1. **List of Appendices**   Appendix 1. Code lists for the primary outcomes |
| 1. **Amendments**   **Amendment 1 – 19/05/2019 (approved)**  **We clarify that aims 1 and 2 (prognostic model derivation and validation) of the study should include the whole population independently of whether patients are new or old users of preventative treatment; the causal inference analyses will focus on new users only:**  *L. Definition of the study population*  Patients will be excluded if they meet the following criteria:  Previous prescription of the exposure variable (antihypertensive, statin or antiplatelet; for causal inference analyses only)  **Amendment 2 – 28/06/2021 (approved 05/07/2021)**  **We have made the following revisions aimed at simplifying the analysis plan in light of the very large sample size and computational time required to conduct each analysis:**   1. *C. Technical Summary / E. Objectives, Specific Aims and Rationale*   Aims:  3) Use causal inference methods (multivariable regression, propensity score adjustment/matching, instrumental variable analysis) to examine whether modification of treatment could have an important impact on the risk of adverse events.  *L. Definition of the study population*  Study entry criteria – Patients will enter the cohort on the index date, defined as:   \| **Exposure** \| Antihypertensive therapy \| Statin therapy \| Antiplatelet therapy \| \| --- \| --- \| --- \| --- \| \| **Index date definition** \| 12 months after the first systolic blood pressure reading ≥130 mm Hg \| 12 months after the first coded or calculated CVD risk of ≥5% (equivalent to age ≥50 years for men and ≥60 years for women) \| 3 months after first CVD event \|   *N. Exposures, Outcomes and Covariates*  Positive controls are outcomes know to be affected by treatment (e.g. cardiovascular disease, death)  For causal inference analyses (only), sensitivity analyses will explore outcomes defined more broadly using primary care codes as well.   1. *O. Data/ Statistical Analysis*   We no longer plan to conduct an internal validation of the prediction models. The following text from the protocol is therefore redundant:  Where feasible and appropriate, each model will be internally validated using the bootstrap method, to allow optimism-adjusted estimates of calibration and discrimination performance to be obtained. Adjustment for overfitting will be undertaken using a uniform shrinkage factor identified from bootstrapping, followed by re-estimation of the cumulative baseline sub-distribution hazard function (and thus baseline cumulative incidence function) to maintain overall calibration between observed and predicted risks. Given the large sample size, overfitting is likely to be small (see sample size calculation above). If bootstrapping is computationally infeasible due to the large sample size, then a smaller random sample of the population will be used or alternative internal validation methods employed such as cross validation.  *Aim 3 – causal inference analysis*  For most of the outcomes described in this proposal, the association and magnitude of effect with treatment is not well defined in randomised controlled trials. We will therefore undertake causal inference work to examine whether modification of treatment could have an important impact on the risk of adverse events using two causal inference methods: multivariable adjustment, propensity score adjustment/matching and instrumental variable analysis.  For the analysis using multivariable adjustment, Cox proportional hazards models will be used to compare adverse event rates in each experimental group. Estimates will be adjusted for pre-specified variables thought to confound the relationship between treatment and adverse events.  For the analysis using propensity scores, we will conduct separate analyses adjusting for propensity score and matching via propensity score.  *Subgroup and sensitivity analyses*  Where possible and appropriate, analyses of treatment associations will be examined in subgroups of the population. These will include age, sex, baseline blood pressure and cardiovascular risk, by drug type/dose/intensity and past medical history. Subgroup definitions will be agreed by expert opinion or taken from an accompanying systematic review of previous trials, examining the same topic. For computational reasons (whereby only one imputation model is required for all subgroup analyses of each outcome), these analyses will only be undertaken using propensity score adjustment.   1. *P. Plan for addressing confounding*   Two approaches will be taken to determine whether observed associations are potentially subject to residual confounding.^46^ Where all approaches provide similar results, we can be more confident that the findings are accurate and reliable. Further steps will be taken to examine the validity of treatment effect estimates using positive and negative controls: the impact of treatment on an outcome known to be affected by treatment (e.g. cardiovascular disease and death; positive controls)  **Amendment 3 – 25.11.2021**  **We have added exploratory analyses using supervised machine learning approaches to address missing data and deal with confounding by indication in the causal inference analyses.**   1. **O. Data/ Statistical Analysis**   ***Aim 3 – causal inference analysis***  For most of the outcomes described in this proposal, the association and magnitude of effect with treatment is not well defined in randomised controlled trials. We will therefore undertake causal inference work to examine whether modification of treatment could have an important impact on the risk of adverse events using two causal inference methods: propensity score matching and instrumental variable analysis.  For the propensity score analysis, eligible patients will be matched 1:1 at the index date using propensity scores, which indicate the likelihood a patient will be prescribed treatment on the basis of their known (pre-treatment) characteristics and other known information which might influence the decision to treat. Predictors of preventative drug prescription will be explored in a logistic regression model. Matched patients will be compared using the Cox proportional hazards models, but hazard ratios will only be adjusted by factors unbalanced at baseline which are not already included in the propensity score model. Time since cohort entry will be used as the underlying time function in each model.  For the instrumental variable analysis, GP’s previous antihypertensive prescribing preferences will be used as an instrument to predict the likelihood of actual treatment. Risk of adverse events will be estimated in patients attending a GP considered a high prescriber of preventative medication and compared to those attending a GP considered a low prescriber. If the results from the propensity score matching suffer from unmeasured confounding, they will differ from those of the instrumental analysis. If this is the case, these models will be refined with additional factors to achieve better adjustment and matching. Where all approaches provide similar results, we can be more confident that the findings are accurate and reliable.^46^  Generative Adversarial Networks (GAN) will also be used to impute missing counterfactual data (unobserved effects of untaken treatments) on an individual level. GAN models are a class of machine learning frameworks that involves the simultaneous training of a pair of deep neural networks in competition with each other. The idea is that the first network, the generator, generates synthetic data samples that are similar to the original samples, while the second network, the discriminator, evaluates the authenticity of the generated samples [1]. Data similarity and fidelity are usually evaluated using well-known metrics such as Maximum Mean Discrepancies [2] and Jensen-Shannon Divergence [3]. While GAN’s initial uses were for imaging applications, its generative capabilities make it naturally suitable for generating missing values that can be used to impute the original samples.  In this work, we build on the intuition that treatment effects estimation and causal inference are a missing data problem, where counterfactuals (unobserved effects of untaken treatments) are missing [4]. Specifically, we plan to use GAN to impute missing counterfactual data, for the samples available in CPRD. By modifying the generator’s goal to accurately impute missing data, and the discriminator’s goal to distinguish between observed and imputed counterfactual values for the same patient record. The model can generate the missing data conditional on the observed values and assigned treatment for each patient as seen in [5, 6]. While useful, the work of [5, 6] are not compatible for time-series data nor for estimating multiple treatment effects, which limits its use in real-life treatment applications. Therefore, we plan to extend such previous works to make them compatible for multiple treatment options and for time-series data to allow for long-term treatment (antihypertensives, statins, antiplatelets) estimation effects for multi-morbid populations as initially proposed in this project.  The proposed GAN approach does not generate new patient samples, but rather generates counterfactual (missing component) conditioned on observed factual data, and outputs a complete record with both factual and counterfactual outcomes to allow for treatment effect estimation for each patient. This approach is naturally immune to re-identification attacks since no new samples are generated, but we will run privacy checks and measures within the data to verify that the model is not copying potential outcomes from patients in the training sample. Example privacy measures that we plan to apply include empirical evaluations such as mathematical privacy definitions such as identifiability [7] that quantifies the probability of re-identification to ensure that no combination of attributes could reveal the identity of a patient. Other privacy evaluation methods include testing that the model is robust against membership inference attacks, where an attacker attempts to determine if a specific patient was used in the training set of the model [8]. Lastly, we will experiment with strict theoretical guarantees such as differential privacy which allow for the model to learn almost nothing about an individual while learning useful information about a population [9].  **References**  [1] Ian Goodfellow, Jean Pouget-Abadie, Mehdi Mirza, Bing Xu, David Warde-Farley, Sherjil Ozair, Aaron Courville, and Yoshua Bengio. Generative adversarial nets. *Advances in neural information processing systems*, 27:2672–2680, 2014.  [2] Arthur Gretton, Karsten M Borgwardt, Malte J Rasch, Bernhard Sch¨olkopf, and Alexander Smola. A kernel two-sample test. *The Journal of Machine Learning Research*, 13(1):723–773, 2012.  [3] AP Majtey, PW Lamberti, and DP Prato. Jensen-shannon divergence as a measure of distinguishability between mixed quantum states. *Physical Review A*, 72(5):052310, 2005.  [4] Peng Ding and Fan Li. Causal inference: A missing data perspective. *Statistical Science*, 33(2):214– 237, 2018.  [5] Jinsung Yoon, James Jordon, and Mihaela Schaar. Gain: Missing data imputation using generative adversarial nets. In *International Conference on Machine Learning*, pages 5689–5698. PMLR, 2018.  [6] Jinsung Yoon, James Jordon, and Mihaela van der Schaar. Ganite: Estimation of individualized treatment effects using generative adversarial nets. In *International Conference on Learning Representations*, 2018.  [7] Jinsung Yoon, Lydia N Drumright, and Mihaela Van Der Schaar. Anonymization through data synthesis using generative adversarial networks (ads-gan). *IEEE journal of biomedical and health informatics*, 24(8):2378–2388, 2020.  [8] Reza Shokri, Marco Stronati, Congzheng Song, and Vitaly Shmatikov. Membership inference attacks against machine learning models. In *2017 IEEE Symposium on Security and Privacy (SP)*, pages 3–18. IEEE, 2017.  [9] Cynthia Dwork, Aaron Roth, et al. The algorithmic foundations of differential privacy. *Found. Trends Theor. Comput. Sci.*, 9(3-4):211–407, 2014.  **Amendment 4 – 30.11.2022**  **Added University of Birmingham to the list of data processors.** |
